# Planar Chirality Controls Diastereotopicity in [2.2]Paracyclophanyl Cyclopropenes

**DOI:** 10.1002/chem.70876

**Published:** 2026-03-25

**Authors:** Tilman Köhler, Maximilian Hartmann, Olaf Fuhr, Stefan Bräse

**Affiliations:** ^1^ Institute of Organic Chemistry (IOC) Karlsruhe Institute of Technology (KIT) Karlsruhe Germany; ^2^ Institute of Biological and Chemical Systems – Functional Molecular Systems (IBCS‐FMS) Karlsruhe Institute of Technology (KIT) Karlsruhe Germany; ^3^ Institute of Nanotechnology (INT) and Karlsruhe Nano Micro Facility (KNMFi) Karlsruhe Institute of Technology (KIT) Karlsruhe Germany

**Keywords:** chirality, cyclopropene, cyclopropenium, paracyclophane, small ring systems

## Abstract

The synthesis of [2.2]paracyclophanyl (pCp) cyclopropenes through rhodium‐catalyzed [2+1] cycloaddition of alkynes with a pCp‐derived carbene is reported. The [2.2]paracyclophane topology induces diastereotopic differentiation of the alkyl substituents in the resulting cyclopropenes, which is consistently observed in their ^1^H NMR spectra. Oxidation to the corresponding cyclopropenium ions removes this stereochemical bias and restores substituent equivalence, reflecting an increase in symmetry upon aromatization.

## Graphical abstract

In this work, we report the synthesis of [2.2]paracyclophane‐substituted cyclopropenes via rhodium‐catalyzed cyclopropenation using a pCp‐derived carbene precursor. The planar chiral framework induces diastereotopic differentiation of otherwise equivalent alkyl substituents, which is reflected in their ^1^H NMR spectra. Oxidation to the corresponding cyclopropenium ions restores substituent equivalence by increasing local symmetry.

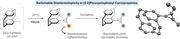

## 1 Introduction

Planar chirality in [2.2]paracyclophane (pCp, **1**) derivatives originates from restricted rotation of the cofacial arene decks, and the resulting enantiomers are configurationally stable under standard reaction conditions [1, 2, 3]. The cyclophane framework tolerates light, oxidative, acidic, and basic environments, and racemization requires markedly elevated temperatures [4]. Consequently, planar chiral pCp constitutes a reliable structural motif in applications where stereochemical integrity must be maintained and has therefore long served as a versatile source of stereochemical information in catalysis and molecular materials [5, 6, 7, 8, 9, 10, 11, 12, 13, 14, 15, 16, 17].

Cyclopropenes, in turn, represent compact, highly strained carbocyclic *π*‐systems whose distinct reactivity and utility as synthetic intermediates have motivated extensive methodological development [18, 19]. Their preparation most commonly follows metal‐catalyzed [2+1] cycloaddition of alkynes with carbene species derived from diazo compounds, a strategy that is largely restricted to electron‐deficient diazo precursors due to the inherent instability of electron‐rich analogues [20, 21, 22, 23, 24, 25]. Recent advances have expanded this methodology through the use of diazo surrogates, enabling access to electron‐donating substrate classes [26, 27]. Notably, a silver triflate‐catalyzed protocol using *N*‐nosylhydrazones has demonstrated the feasibility of donor‐substituted carbene intermediates in cyclopropenation, while *N*‐triftosylhydrazones have proven to be highly versatile carbene precursors across a wide range of reaction manifolds [28, 29].

Cyclopropenium ions represent the smallest Hückel‐aromatic carbocations. In recent years, their utility in catalysis has been increasingly recognized, yet cyclopropenium ions remain comparatively underexplored in organic synthesis [30, 31, 32, 33]. Importantly, fully substituted cyclopropenes can often be readily converted into the corresponding cyclopropenium ions by hydride abstraction using strong hydride acceptors such as triphenylcarbenium salts [34]. This straightforward transformation provides a convenient experimental handle to probe the associated structural and spectroscopic consequences.

**SCHEME 1:**
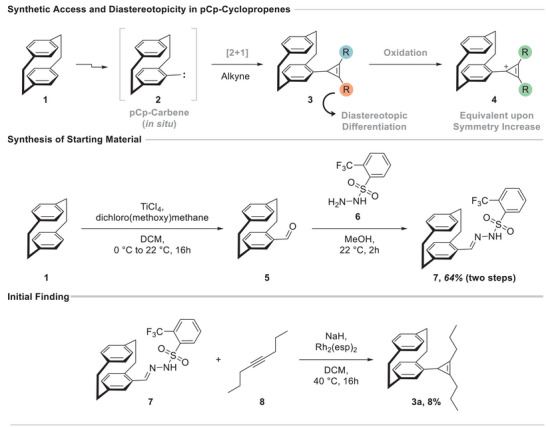
(Top) pCp breaks local symmetry in cyclopropenes and causes diastereotopic substituents, whereas oxidation increases symmetry and restores equivalence. (Middle) Synthesis of triftosylhydrazone **6**. (Bottom) Initial cyclopropenation result.

Despite the widespread use of planar chiral pCp scaffolds as stereochemical information carriers, their capacity to locally break symmetry in small, carbocyclic *π*‐systems has remained largely unexplored. Attaching such a planar chiral unit to a cyclopropene creates a compact model system in which substituent equivalence, or its loss, can be directly assessed by NMR spectroscopy. We envisioned that direct access to pCp‐substituted cyclopropenes **3** could be achieved through transition‐metal‐catalyzed [2+1] cyclopropenation between an alkyne and a pCp‐derived carbene **2**. Realizing this transformation requires accommodating both the pronounced steric demand and the electron‐donating character of the pCp unit. We previously showed that these challenges can be overcome by employing a pCp‐derived *N*‐triftosylhydrazone **7** under rhodium catalysis in the synthesis of pCp‐quinoline derivatives, which provided a foundation for the present approach [35]. Establishing such synthetic access, together with the corresponding oxidation to cyclopropenium ions **4**, enables spectroscopic and structural examination of how planar chirality influences small strained carbocycles (Scheme 1).

## 2 Results and Discussion

### 2.1 Initial Findings

We commenced our study with the synthesis of [2.2]paracyclophane‐substituted triftosylhydrazone **7**, which was prepared by formylation of [2.2]paracyclophane (**1**), followed by condensation of the resulting aldehyde **5** with 2‐(trifluoromethyl)benzenesulfonylhydrazide (**6**). The target compound **7** was readily obtained by simple filtration, rendering the overall sequence chromatography‐free on a gram scale with 64% overall yield. With diazo surrogate **7** in hand, we next explored suitable conditions for the desired cyclopropenation. Using Davies catalyst Rh_2_(esp)_2_ and 3‐octyne (**8**) as the alkyne coupling partner in the presence of NaH in dichloromethane (DCM) at 40°C for 16 h, the corresponding pCp‐substituted cyclopropene **3a** was obtained in 8% isolated yield (Scheme 1).

**FIGURE 1:**
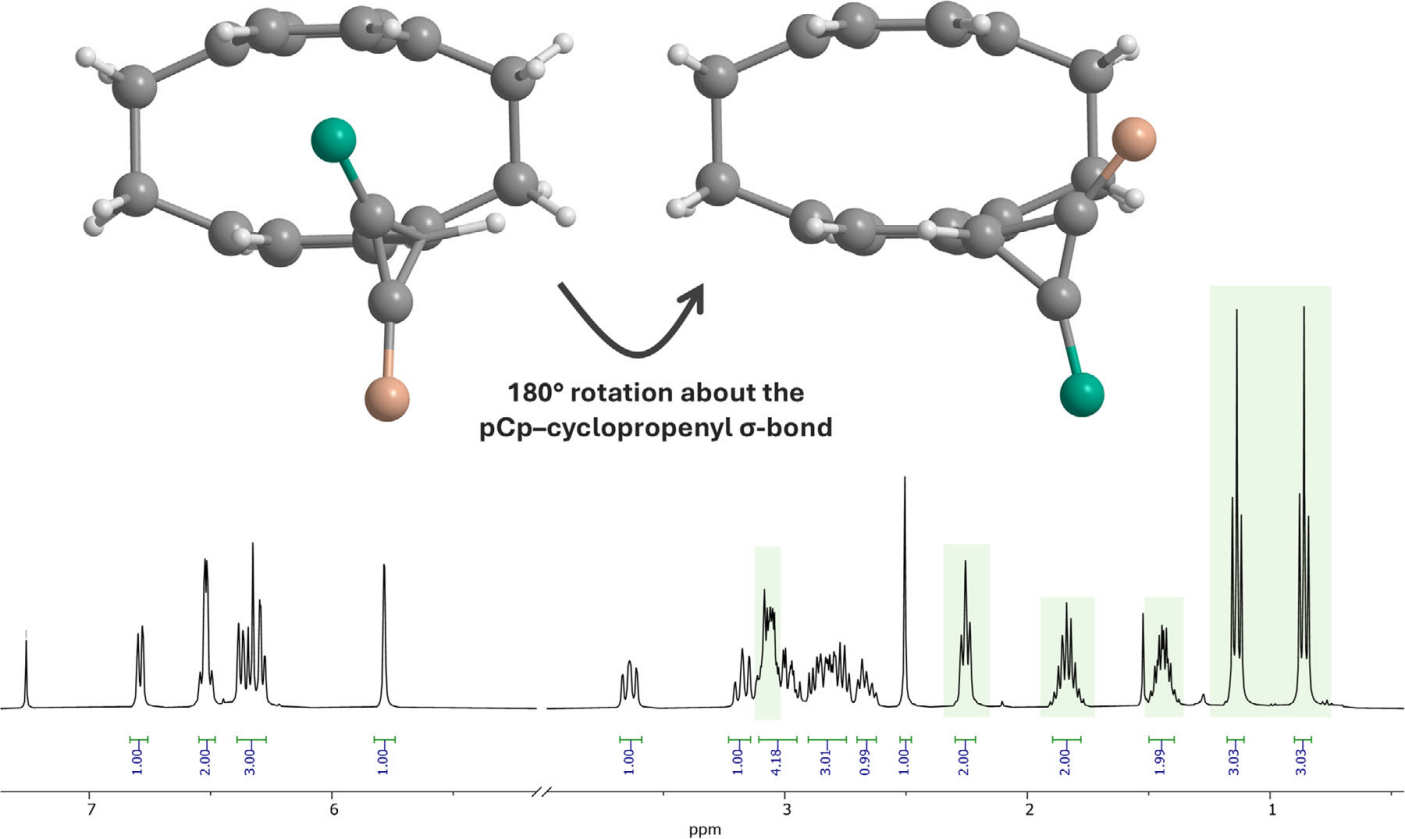
Conformers related by a 180° rotation about the pCp–cyclopropenyl *σ*‐bond and diastereotopic alkyl resonances in the ^1^H NMR spectrum of cyclopropene **3a**. Propyl substituents are shown generically in two different colors for clarity.

Importantly, the propyl groups on cyclopropene **3a** appear anisochronous in the ^1^H NMR spectrum, with each propyl chain giving a distinct set of multiplets. This diastereotopic differentiation originates from the stereochemical bias imposed by the planar chiral [2.2]paracyclophane unit, such that the two substituents occupy inequivalent chemical environments. In an achiral reference system, the two alkyl substituents at the cyclopropene ring would be enantiotopic and thus NMR‐equivalent, while the cyclopropene CH center is prochiral. However, the planar chirality of the pCp scaffold converts these enantiotopic substituents into diastereotopic groups. Illustratively, a 180° rotation about the pCp–cyclopropene *σ*‐bond does not generate a symmetry‐equivalent conformation and therefore does not interconvert the two propyl groups. In this sense, the substituents are chirotopic within the planar chiral environment, as defined by MISLOW and SIEGEL (Figure 1) [36].

While such diastereotopic differentiation is not unique to [2.2]paracyclophane (**1**) and would in principle be induced by any configurationally stable chiral substituent, the use of planar chirality, as provided by the pCp scaffold, to control substituent differentiation in cyclopropenes has, to the best of our knowledge, not been experimentally demonstrated prior to this work.

### 2.2 Reaction Development and Scope

Encouraged by this intriguing stereochemical feature, we next aimed to develop a robust and reproducible reaction protocol (see Table S1 for details). Rh_2_(esp)_2_ proved superior to all alternative catalytic systems examined. Other rhodium(II) and various silver(I) catalysts resulted in diminished yields or complex reaction mixtures, while Fe‐based systems as well as rhodium(III) catalysts showed no reactivity. Subsequent optimization identified the alkyne loading and the addition of NaBARF as a weakly coordinating anion as key parameters for improving reaction efficiency. Variations in solvent and temperature did not lead to further enhancement.

**SCHEME 2:**
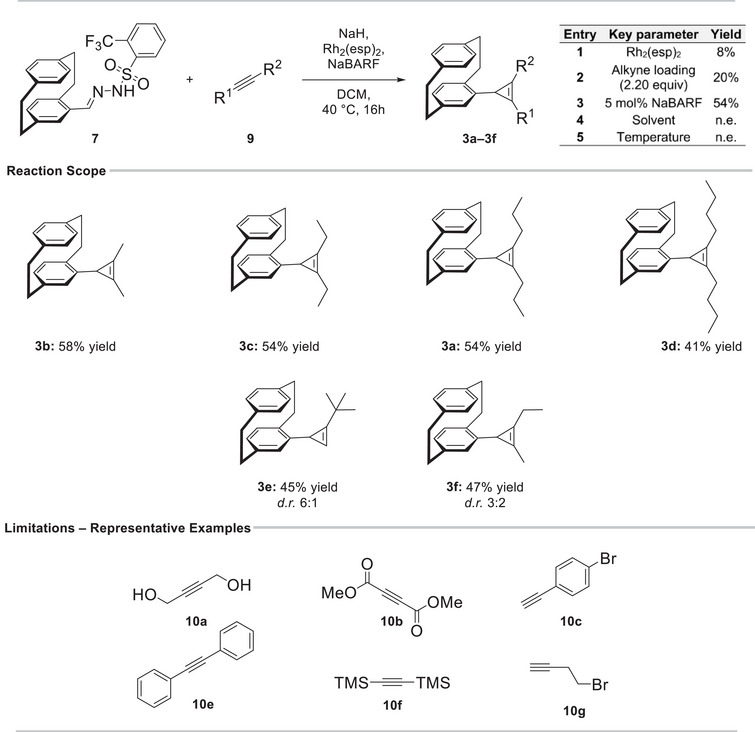
Scope and limitations of the Rh‐catalyzed cyclopropenation. Yields refer to isolated products. n.e. = no enhancement. d.r. = diastereomeric ratio.

The optimized conditions thus were defined as Rh_2_(esp)_2_ (5 mol%), 2.20 equivalents of the alkyne, and NaBARF as additive in DCM at 40°C for 16 h, affording the desired product **3a** in 54% isolated yield (Scheme 2).

With the optimized conditions established, we next investigated the substrate scope of the transformation. Linear aliphatic internal alkynes were examined first and afforded the corresponding aliphatic cyclopropenes **3a**–**3d** in good yields. The terminal alkyne 3,3‐dimethylbut‐1‐yne was likewise well tolerated despite its sterically demanding *tert*‐butyl group, giving a diastereomeric product mixture in a 6:1 ratio. This outcome is consistent with the fact that unsymmetrical substitution renders the cyclopropene CH center stereogenic. In addition, 2‐pentyne underwent efficient conversion to the expected unsymmetrical cyclopropene **3f** in good yield, forming a 3:2 mixture of diastereomers (Scheme 2, see Figures S9–S11 for details).

**FIGURE 2:**
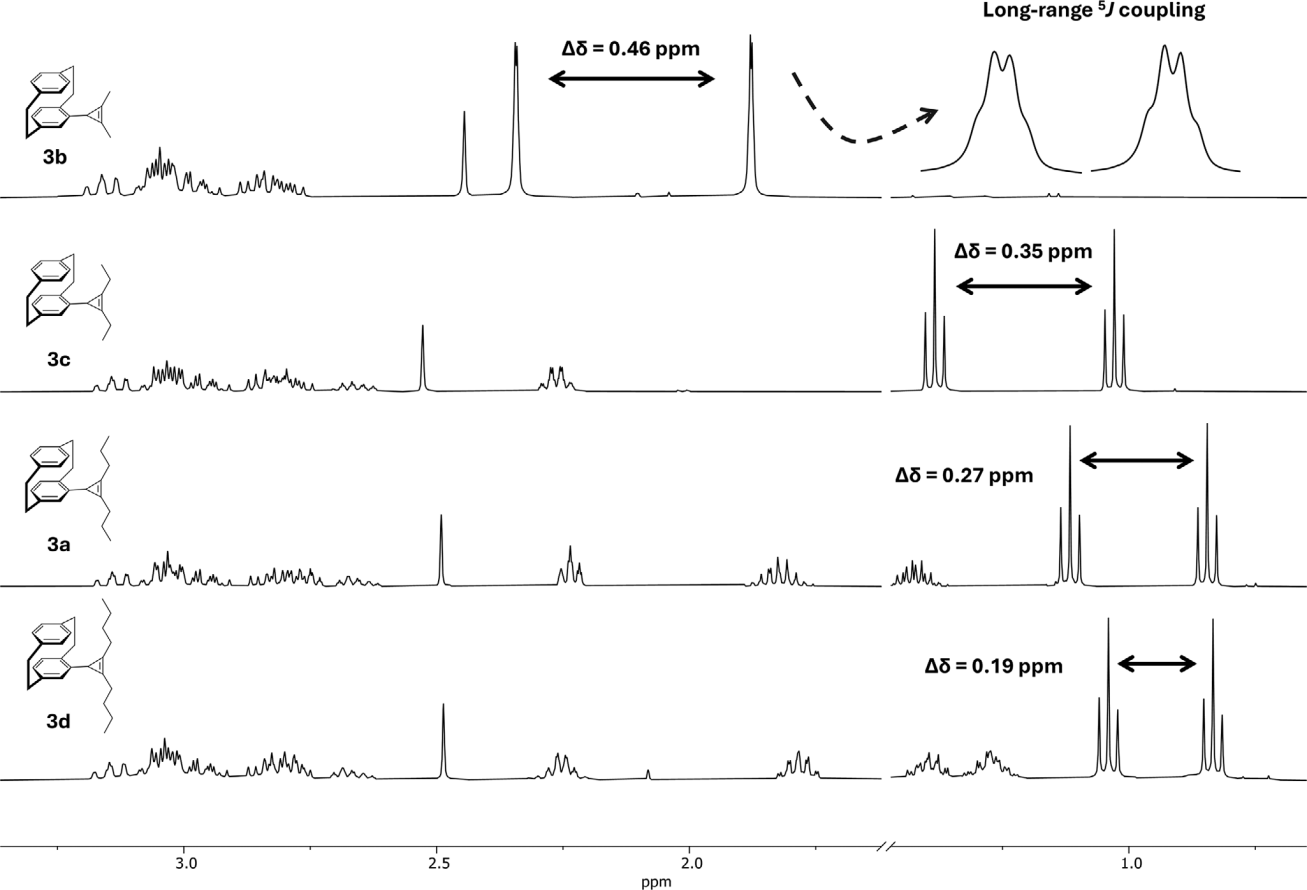
^1^H NMR spectra of cyclopropenes **3a–3d**. Only the aliphatic region is shown, and parts of the remaining spectrum were omitted for clarity.

However, all attempts to use functionalized alkynes in this transformation were unsuccessful, resulting exclusively in recovery of the parent pCp‐aldehyde precursor of triftosylhydrazone **5** (see Scheme S1 for full list of substrates). A cross‐substrate experiment revealed that aliphatic alkynes still delivered the cyclopropene product **3a** in the presence of a functionalized alkyne, ruling out catalyst deactivation by the functionalized substrates. Furthermore, we examined the use of alternative bases such as DBU and Cs_2_CO_3_, but these did not result in any improvement. One possible explanation for this limitation is that functionalized alkynes engage in competing pathways such as oligomerization under the reaction conditions. In these cases, the reaction mixtures turned into viscous, resin‐like material, although this has not been investigated further. Interestingly, diphenylacetylene (**10e**) also proved incompatible, most likely due to steric hindrance. Diastereotopicity of the alkyl groups was observed for all symmetrical cyclopropenes **3a**–**3d**, with substantial chemical shift separations in each case. This effect is particularly evident for cyclopropene **3b**, where the methyl resonances appear at *δ* = 2.34 and 1.88 ppm (Δ*δ* = 0.46 ppm). For compounds **3b** and **3f**, the methyl group attached to the cyclopropene ring notably exhibited a long‐range ^5^ *J* coupling to the protons of the opposing alkyl substituent, with coupling constants of 1.4 and 1.6 Hz, respectively (Scheme 2 and Figure 2).

### 2.3 Cyclopropenium Synthesis

**SCHEME 3:**
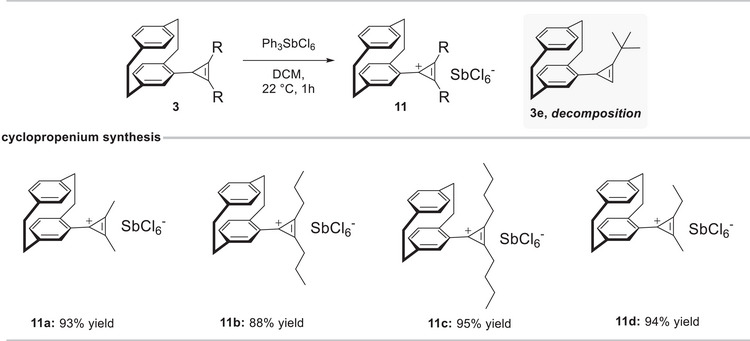
Cyclopropenium salt synthesis. Yields refer to isolated products.

**FIGURE 3:**
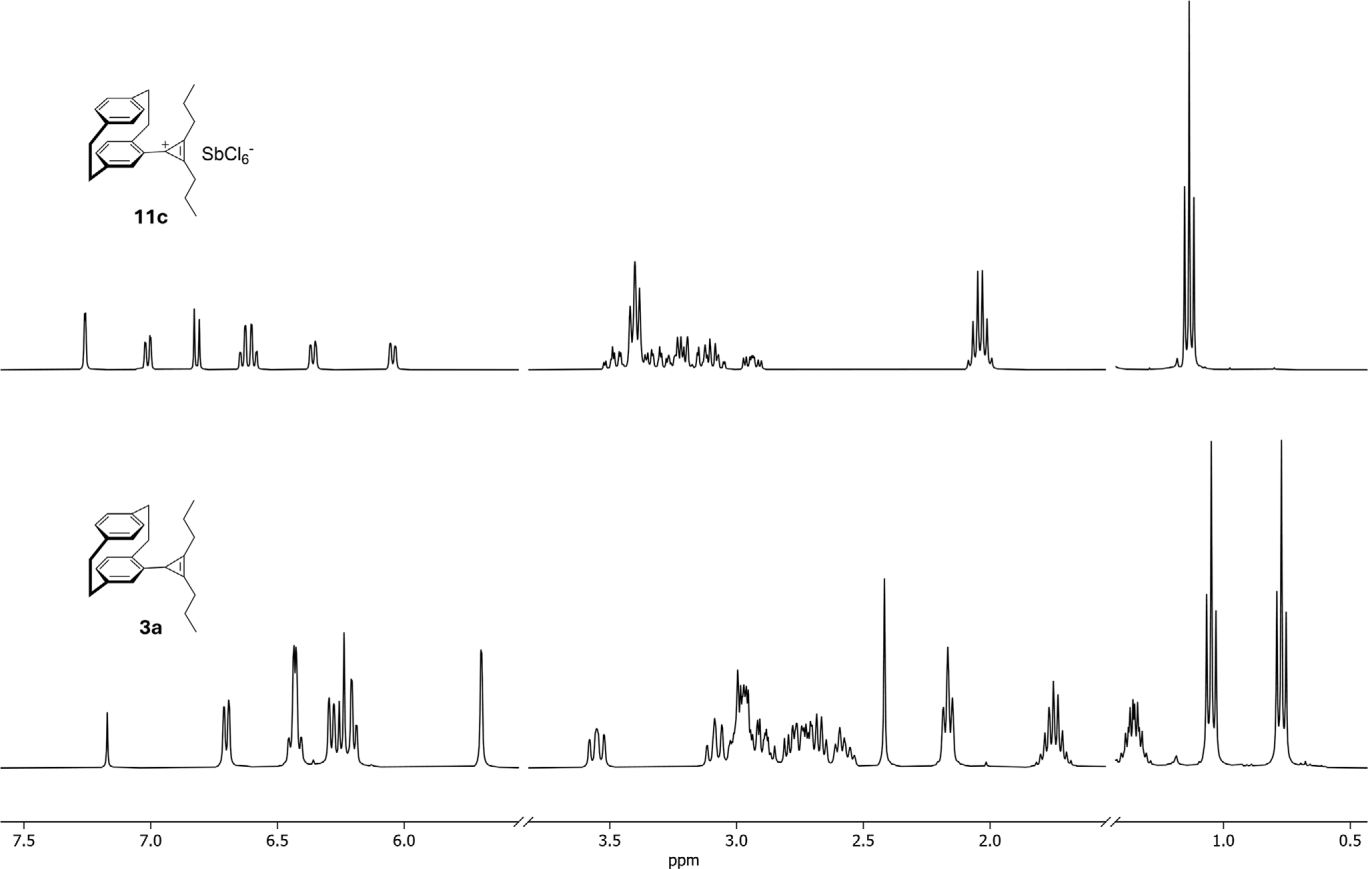
Comparison of the ^1^H NMR spectra of representative pCp‐substituted cyclopropenium salt **11c** (top) and the corresponding cyclopropene **3a** (bottom). Parts of the spectra were omitted for clarity.

**FIGURE 4:**
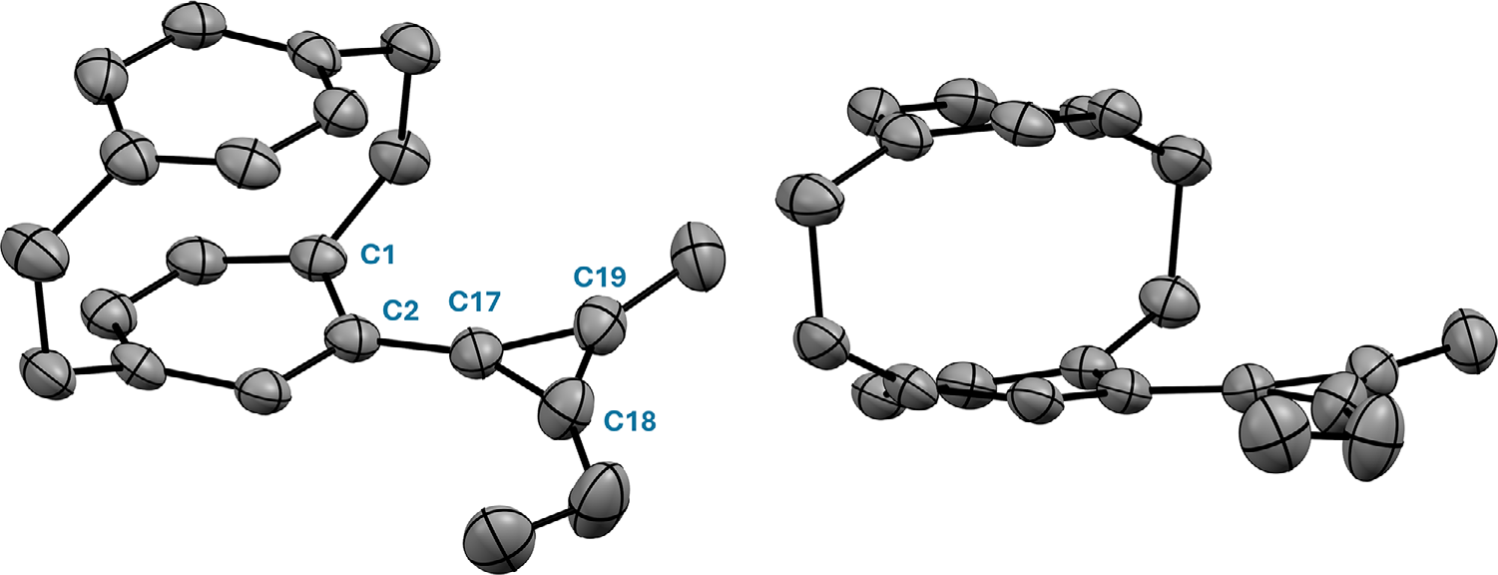
Molecular structure of the pCp‐substituted cyclopropenium cation **11d** (50 % probability ellipsoids). Hydrogen atoms and the hexachloroantimonate anion are omitted for clarity (see Figure S2 and Table S2 for details).

With these results in hand, we reasoned that oxidation of the cyclopropenes to the corresponding cyclopropenium ions provides a direct experimental probe of the observed diastereotopicity. Conversion to the aromatic cyclopropenium cation establishes a local rotational symmetry of the pCp–cyclopropenyl fragment and is therefore expected to restore substituent equivalence. Accordingly, several pCp–cyclopropenes (**3a**, **3b**, **3d**, **3f**) were treated with triphenylcarbenium hexachloroantimonate (Ph_3_SbCl_6_) in DCM at 22°C. Shortly after addition of the triphenylcarbenium salt, the reaction mixtures developed a greenish tint. After 1 h of reaction time, the corresponding cyclopropenium salts **11a**–**11d** were readily isolated by simple precipitation with diethyl ether, affording the products as yellow solids in high yield (88%–95%). As expected, the bis‐substituted cyclopropene **3e** did not furnish the corresponding cyclopropenium salt but instead underwent decomposition under the reaction conditions. Indeed, conversion to the cyclopropenium cation removes the diastereotopic differentiation of the alkyl substituents in the ^1^H NMR spectrum. Furthermore, the aromatic ring current changes significantly, in line with aromatic delocalization between the pCp aromatic deck and the cyclopropenium ring (Scheme 3 and Figure 3).

x‐Ray analysis of single crystals of cyclopropenium salt **11d** obtained through slow‐diffusion techniques confirmed a well‐defined pCp‐substituted cyclopropenium cation. The cyclopropenium ring is essentially planar, with C─C bond lengths of 1.338(15) –1.403(14) Å and highly acute internal ring angles at C17, C18, and C19 of 57.6(7)°, 62.1(7)°, and 60.3(7)°, respectively. The cation is attached to the [2.2]paracyclophane framework at C2 with a C2─C17 bond length of 1.420(13) Å. Notably, the cyclopropenium plane is oriented nearly coplanar with the substituted pCp arene deck, as reflected by the near‐linear torsion angles C1–C2–C17–C18 = −176.0(17)° and C1–C2–C17–C19 = 1(2)°. This geometry maximizes π‐conjugation between the cyclopropenium unit and the adjacent aryl deck, consistent with delocalization across the cyclopropenium–pCp aryl fragment (Figure 4).

Although the crystallographically characterized derivative **11d** is unsymmetrical, the structural features revealed are expected to extend to the symmetrically substituted cyclopropenium salts **11a**–**11c**. In contrast to the corresponding cyclopropenes **3a**, **3b**, and **3d** discussed above, a 180° rotation about the pCp–cyclopropenium bond constitutes a symmetry operation that maps the two alkyl substituents onto one another within the planar chiral pCp framework. Consequently, the two alkyl substituents are chemically equivalent. The crystal structure of **11d** is compatible with this symmetry description.

**FIGURE 5:**
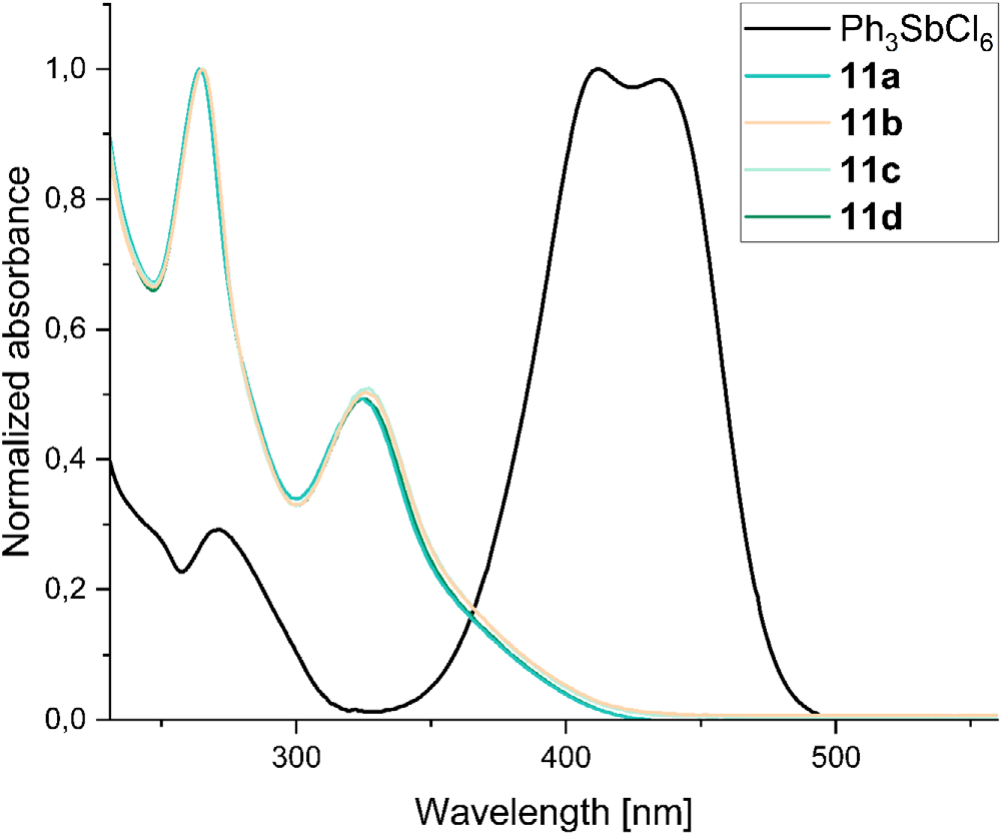
UV/Vis absorption spectra of triphenylcarbenium hexachloroantimonate (Ph_3_SbCl_6_) and pCp‐substituted cyclopropenium salts **11a**–**11d** recorded in DCM at 22°C. All spectra were normalized to their respective absorption maxima for comparison.

Building on this structural insight, we examined the electronic properties of the cyclopropenium cations and recorded UV/Vis spectra. All pCp‐substituted cyclopropenium salts **11a**–**11d** display nearly identical absorption profiles, despite the variation of the alkyl substituents at the cyclopropenium ring, indicating that the dominant electronic transitions are only weakly perturbed by alkyl substitution. They exhibit two characteristic absorption bands with maxima at approximately 265 and 325 nm, which can be assigned to *π*–π* transitions of the conjugated cyclopropenium–arene system. Although these transitions are confined to the ultraviolet region, the salts appear yellow due to weak absorption tails extending toward the visible range. In clear contrast, the triphenylcarbenium salt shows an intense long‐wavelength absorption in the visible region with maxima at 412 and 435 nm, which is absent for all cyclopropenium derivatives (Figures 5, see Figures S5–S8 for details).

### 2.4 Rotational Preferences of Cyclopropene Versus Cyclopropenium

To further understand the conformational preferences of the pCp–cyclopropene and pCp–cyclopropenium systems, and to exclude hindered bond rotation as the origin of the observed diastereotopicity, rotation about the pCp–C(cyclopropenyl) *σ*‐bond in cyclopropene **3b** was evaluated by DFT (PBE0/def2‐TZVP). The calculated rotational energy profile is essentially flat, with barriers of only 5–6 kcal mol^−^^1^, consistent with rapid rotation on the NMR time scale at ambient temperature, as further supported by a high‐temperature NMR experiment (see Figure S1). Importantly, the two conformations **A** and **B** connected by a 180° rotation differ in energy by approximately 2.6 kcal mol^−^^1^, demonstrating that they are indeed not symmetry equivalent. These results support the conclusion that the observed diastereotopicity arises from symmetry inequivalence rather than restricted rotation.

**FIGURE 6:**
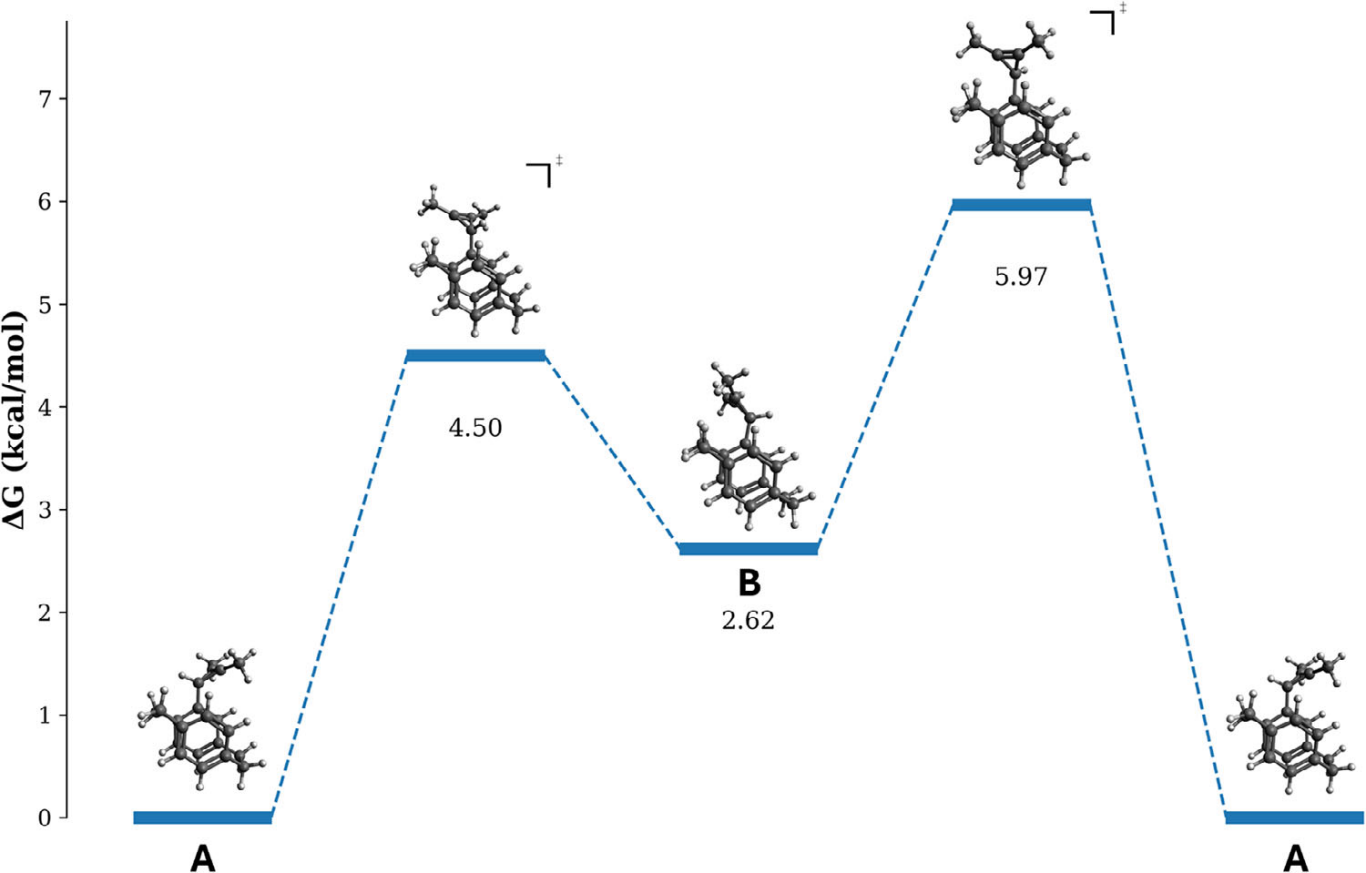
DFT‐calculated rotational free‐energy profile for cyclopropene **3b**. Conformer **A** and conformer **B** denote the two minima connected by a 180° rotation about the pCp–C(cyclopropenyl) *σ*‐bond.

In contrast, the corresponding cyclopropenium cation **11a** exhibits a more structured rotational profile, with a barrier of approximately 10 kcal mol^−^^1^. This behavior is chemically intuitive, as rotation away from coplanarity diminishes *π*‐conjugative interactions between the cyclopropenium unit and the adjacent pCp aromatic deck. Notably, this calculated conformational preference is in good agreement with the crystal structure of cyclopropenium **11d** (Figure 6, see Figures S3 and S4 for details).

## 3 Conclusion

We present a synthetic approach to pCp‐substituted cyclopropenes based on rhodium‐catalyzed cyclopropenation using a pCp‐derived carbene precursor. The resulting cyclopropenes show diastereotopic differentiation of their cyclopropene‐ring substituents in their ^1^H NMR spectra. Oxidation to the corresponding cyclopropenium ions removes this feature, reflecting an increase in the local symmetry of the pCp–cyclopropenium fragment. This is further supported by single‐crystal x‐ray diffraction, which reveals a geometry compatible with rotational equivalence. In addition, calculated rotational energy profiles show that the observed effect does not originate from restricted rotation.

Taken together, these results demonstrate that planar chirality can modulate local symmetry in this compact model system to the extent that otherwise equivalent substituents become spectroscopically distinguishable, and that oxidation reverses this effect. The study thus links a controllable change in symmetry to directly observable spectroscopic signatures and provides a minimal structural framework in which this relationship can be examined experimentally.

## Conflicts of Interest

The authors declare no conflicts of interest.

## Supporting information




**Supplementary File 1**: The authors have cited additional references within the Supporting Information [1–14].

## Data Availability

The reaction descriptions and analytical data for this Supporting Information were generated automatically by the Chemotion electronic laboratory notebook (ELN, version 3.0). This section covers detailed material on the experiments and their results, including the characterization of the compounds obtained. The data that support the findings of this publication are available in the repository Chemotion (www.chemotion‐repository.net). All DOIs minted for the data are linked to the specific experiments in this section, and a summary of all new data obtained in this thesis can be gained with the collection DOI https://dx.doi.org/10.14272/collection/TIK_2024‐12‐16. Crystallographic data for compound 11d reported in this paper have been deposited with the Cambridge Crystallographic Data Centre as Supporting Information no. CCDC‐2524363. Copies of the data can be obtained free of charge from https://www.ccdc.cam.ac.uk/structures/.
