# Supplementary material for: Planar Chirality Controls Diastereotopicity in [2.2]Paracyclophanyl Cyclopropenes
Source: Chemistry. 2026 Mar 25;32(16):e70876. doi: 10.1002/chem.70876 (PMC13109690; doi:10.1002/chem.70876)
Supplement: Supplementary file 1 — The authors have cited additional references within the Supporting Information [1–14]. [file CHEM-32-e70876-s001.pdf]

Planar Chirality Controls Diastereotopicity in [2.2]Paracyclophanyl Cyclopropenes

Tilman Köhler,[a] Maximilian Hartmann,[b] Olaf Fuhr,[c] and Stefan Bräse[a,b]*

[a]Institute of Organic Chemistry (IOC), Karlsruhe Institute of Technology (KIT), Kaiserstraße 12, 76131 Karlsruhe, Germany. Phone: (+49)-721-6084-2903; E-mail: braese@kit.edu.

[b]Institute of Biological and Chemical Systems – Functional Molecular Systems (IBCS-FMS), Karlsruhe Institute of Technology (KIT), Hermann-von-Helmholtz-Platz 1, 76344 Eggenstein-Leopoldshafen, Germany.

[c]Institute of Nanotechnology (INT) and Karlsruhe Nano Micro Facility (KNMFi), Karlsruhe Institute of Technology (KIT), Hermann-von-Helmholtz-Platz 1, 76344 Eggenstein-Leopoldshafen, Germany.

* Corresponding author

Table of Contents

1. Data Availability Statement 1

2. General Remarks 1

3. Synthetic Procedures 4

3.1. Reaction Optimization 16

3.2. Limitations: Functionalized Alkynes 18

3.3. High-Temperature NMR Experiment 18

4. X-ray Diffraction Data 19

5. Computational methods 21

6. Spectra 23

6.1. NMR Spectra 23

6.2. UV/VIS spectra 36

6.3. NOESY Experiment 38

7. References 40

1. Data Availability Statement

The reaction descriptions and analytical data for this supplementary information were generated automatically by the Chemotion electronic laboratory notebook (ELN, version 1.90).

This section covers detailed material on the experiments and their results, including the characterization of the compounds obtained. The data that support the findings of this publication are available in the repository Chemotion (https://www.chemotion-repository.net). All DOIs minted for the data are linked to the specific experiments in this section, and a summary of all new data obtained in this thesis can be gained with the collection DOI https://dx.doi.org/10.14272/collection/TIK_2024-12-16.[1–3]

2. General Remarks

Materials and Methods

The starting materials, solvents, and reagents were purchased from ABCR, ACROS, ALFA AESAR, APOLLO SCIENTIFIC, CARBOLUTION, CHEMPUR, FLUKA, FLUOROCHEM, MERCK, RIEDEL-DE HAËN, SIGMA ALDRICH, STREM, TCI, or THERMO FISHER SCIENTIFIC and used without further purification unless stated otherwise.

Solvents of technical quality were purified by distillation or with the solvent purification system MB SPS5 (acetonitrile, dichloromethane, diethyl ether, tetrahydrofuran, toluene) from MBRAUN. Solvents of *p.a.* quality were purchased from ACROS, FISHER SCIENTIFIC, SIGMA ALDRICH, Roth, or RIEDEL-DE HAËN and were used without further purification.

Abbreviations: cHex = cyclohexane, EtOAc = ethyl acetate, THF = tetrahydrofuran, DCM = dichloromethane, MeOH = methanol.

Oxygen-free solvents were obtained by freeze-pump-thaw technique (three cycles).

Air- and moisture-sensitive reactions were carried out under an argon atmosphere in oven-dried glassware using standard Schlenk techniques. Liquids were added via cannula, and solids were added in powder form.

Reactions at low temperatures were cooled using flat dewars produced by ISOTHERM (Karlsruhe) with water/ice mixtures.

Solvents were evaporated under reduced pressure at 40 °C using a rotary evaporator. For solvent mixtures, each solvent was measured volumetrically.

Crude products were purified by automated flash chromatography using a Büchi PURE C-815 Flash system equipped with UV–Vis (200–800 nm, halogen/deuterium lamp, DAD scan) and ELSD detectors. FlashPure EcoFlex silica gel cartridges (4–24 g, 15–60 µm particle size; Büchi) served as the stationary phase. Alternatively, purifications were performed on a Biotage Isolera One system using puriFlash F0025 columns.

Reaction Monitoring

All reactions were monitored by thin-layer chromatography (TLC) using silica-coated aluminum plates (MERCK, silica 60, F254). UV active compounds were detected with a UV lamp at 254 nm and 366 nm excitation.

GC-MS (gas chromatography-mass spectrometry) measurements were performed on an AGILENT TECHNOLOGIES model 6890N (electron impact ionization), equipped with an AGILENT 19091S-433 column (5% phenyl methyl siloxane, 30 m, 0.25 µm) and a 5975B VL MSD detector with a turbopump. Helium was used as a carrier gas.

Melting Point

Melting points were detected on an OptiMelt MPA100 device from the STANFORD RESEARCH SYSTEM.

Nuclear Magnetic Resonance Spectroscopy (NMR)

NMR spectra were recorded on a BRUKER Avance 400 NMR instrument at 400 MHz for ^{1}H NMR, 101 MHz for ^{13}C NMR, 376 MHz for ^{19}F NMR, or a BRUKER Avance 500 NMR instrument at 500 MHz for ^{1}H NMR, 126 MHz for ^{13}C NMR and 470 MHz for ^{19}F NMR.

The NMR spectra were recorded at room temperature in deuterated solvents acquired from EURISOTOP, SIGMA ALDRICH, or DEUTERO. The chemical shift δ is displayed in parts per million [ppm], and the references used were the ^{1}H and ^{13}C peaks of the solvents themselves:

d_1-chloroform ($CDCl_3$): 7.26 ppm for ^{1}H and 77.16 ppm for ^{13}C

d_3-acetonitrile (MeCN): 1.94 ppm for ^{1}H and 118.26 ppm for ^{13}C

d_2-dichloromethane (DCM): 5.32 ppm for ^{1}H and 53.8 ppm for ^{13}C

For the characterization of centrosymmetric signals, the signal's median point is given, for multiplets, the signal range. The following abbreviations are used to describe the proton splitting pattern: d = doublet, t = triplet, m = multiplet, dd = doublet of a doublet, ddd = doublet of doublet of a doublet, dddd = doublet of doublet of doublet of a doublet, dt = doublet of a triplet. Absolute values of the coupling constants "*J*" are given in Hertz [Hz] and decreasing order. Signals of the ^{13}C spectrum are assigned by distortionless enhancement by polarization transfer (DEPT) spectra DEPT90 and DEPT135 or phase-edited heteronuclear single quantum coherence (HSQC). They are specified in the following way: C_q = quaternary carbon atoms, *C*H = tertiary carbon atoms, CH_2 = secondary carbon atoms, and CH_3 = primary carbon atoms.

Infrared Spectroscopy (IR)

The infrared spectra were recorded with a BRUKER Alpha P instrument. All samples were measured by attenuated total reflection (ATR). The positions of the absorption bands are given in wavenumbers $\tilde{\nu}$ in cm^{-1} and were measured in the range from 3600 cm^{-1} to 350 cm^{-1}.

The absorption bands were characterized according to their absorption strength, using the following abbreviations: vs (very strong, 0−9%), s (strong, 10−39%), m (medium, 40−69%), w (weak, 70−89%), and vw (very weak, 90−100%).

Mass Spectrometry (MS)

APCI (atmospheric pressure chemical ionization) and ESI (electrospray ionization) experiments were recorded on a Q-Exactive (Orbitrap) mass spectrometer (THERMO FISHER SCIENTIFIC, San Jose, CA, USA) equipped with a HESI II probe to record high resolution. The tolerated error is ±5 ppm of the molecular mass. The spectra were interpreted by molecular peaks $[M]^+$ or peaks of protonated molecules $[M+H]^+$ and characteristic fragment peaks and indicated with their mass-to-charge ratio (m/z) and intensity in percent relative to the base peak (100%).

Liquid Chromatography − Mass Spectrometry (LC−MS)

Liquid Chromatography − Mass Spectrometry (LC−MS) was performed using a THERMOFISHER UltiMate 3000 system containing a degasser, pump, autosampler, column compartment, and diode array detector coupled with an ISQ^{TM} EM Single Quadrupole Mass Spectrometer system with ESI-source. The flow rate was 0.45 mL/min on a stationary KINETEX XB-C18 column (2.1 mm × 100 mm, 2.6 µm particle size).

3. Synthetic Procedures

(*rac*)-4-Formyl[2.2]paracyclophane (5)

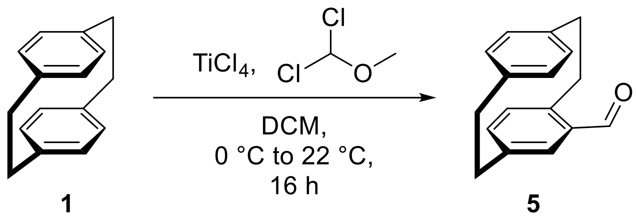

A 1 L round-bottom flask equipped with a stirring bar was charged with [2.2]paracyclophane (6.50 g, 31.2 mmol, 1.00 equiv) (**1**) and DCM (200 mL). The mixture was cooled to 0 °C and titanium tetrachloride (11.8 g, 6.84 mL, 62.4 mmol, 2.00 equiv) was added, followed by the slow addition of dichloro(methoxy)methane (3.77 g, 2.96 mL, 32.8 mmol, 1.05 equiv). After stirring for 15 min at 0 °C, the reaction was stirred for 16 h at 22 °C. The mixture was then poured onto ice (200 mL) and stirred for 2 h. The organic layer was separated, and the aqueous layer was extracted with DCM (3 × 50 mL). The combined organic layers were washed with aqueous NH_4Cl (3 × 50 mL) and brine (50 mL), dried over $MgSO_4$ and the solvent was removed under reduced pressure. The crude product was recrystallized from *n*-hexane to afford the target compound (*rac*)-4-formyl[2.2]paracyclophane (**5**) as a colorless solid in 87% yield (6.43 g, 27.2 mmol).

Melting point range: 159–161 °C

R_f-value: 0.54 (cHex/EtOAc 5:1)

^{1}H NMR (400 MHz, Chloroform-*d* [7.26 ppm], ppm) δ = 9.95 (s, 1H), 7.02 (d, *J* = 1.0 Hz, 1H), 6.73 (dd, *J* = 7.6 Hz, *J* = 1.3 Hz, 1H), 6.60–6.55 (m, 2H), 6.51–6.49 (m, 1H), 6.44–6.37 (m, 2H), 4.13–4.08 (m, 1H), 3.29–2.91 (m, 7H).

^{13}C NMR (100 MHz, Chloroform-*d* [77.2 ppm], ppm) δ = 192.0 (*C*H), 143.3 (C_q), 140.8 (C_q), 139.6 (C_q), 139.6 (C_q), 138.2 (*C*H), 136.7 (C_q), 136.5 (*C*H), 136.2 (*C*H), 133.4 (*C*H), 133.0 (*C*H), 132.5 (*C*H), 132.3 (*C*H), 35.4 (CH_2), 35.3 (CH_2), 35.1 (CH_2), 33.8 (CH_2).

MS (ESI+, DCM) m/z (%): 237 (100) $[M + H]^+$.

HRMS (ESI+, DCM) m/z (%) calcd for $C_{17}H_{18}O$ $[M + H]^+$: 237.1273; found 237.1273.

IR (ATR, ṽ) = 2951 (w), 2924 (w), 2888 (w), 2850 (w), 2748 (w), 1497 (w), 1486 (w), 1409 (w), 1401 (w), 1282 (w), 1225 (m), 1204 (w), 1180 (w), 1142 (m), 1115 (w), 1101 (w), 976 (w), 942 (w), 936 (w), 905 (m), 874 (s), 795 (s), 772 (m), 744 (w), 718 (vs), 657 (w), 635 (vs), 623 (vs), 574 (m), 516 (vs), 494 (m), 462 (w), 450 (w), 438 (w), 428 (w), 387 (w) cm^{-1}.

The analytical data are consistent with the literature.[4]

Additional information on the experimental procedure is available via the repository Chemotion: https://dx.doi.org/10.14272/reaction/SA-FUHFF-UHFFFADPSC-BAIBHOHKSY-UHFFFADPSC-NUHFF-NUHFF-NUHFF-ZZZ.4

Additional information on the characterization of the target compound is available via the repository Chemotion: https://dx.doi.org/10.14272/BAIBHOHKSYVVCM-UHFFFAOYSA-N.7

2-(Trifluoromethyl)benzenesulfonohydrazide (6)

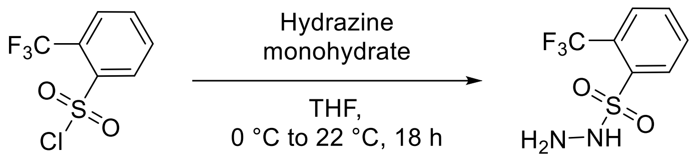

6

A 250 mL round-bottom flask equipped with a stirring bar was charged with 2-(trifluoromethyl)benzenesulfonyl chloride (6.44 g, 4.06 mL, 25.0 mmol, 1.00 equiv) and THF (40 mL) and the mixture was cooled to 0 °C. Hydrazine monohydrate (2.50 g, 2.43 mL, 50.0 mmol, 2.00 equiv) was added dropwise and the reaction was stirred at 22 °C for 18 h. Then, the reaction was diluted with EtOAc (50 mL) and water (50 mL) and the layers were separated. The organic layer was washed with brine (20 mL), dried over $MgSO_4$, filtered, and the solvent was removed under reduced pressure. The residue was dissolved in EtOAc (30 mL) under heating and was then added to *n*-hexane (300 mL) under vigorous stirring. The precipitate was collected and dried under high vacuum for 1 h to obtain the target compound 2-(trifluoromethyl)benzenesulfonohydrazide (**6**) as a colorless solid in 84% yield (5.05 g, 21.0 mmol).

Melting point range: 119–120 °C

R_f-value: 0.75 (DCM)

^{1}H NMR (400 MHz, Acetonitrile-d_3 [1.94 ppm], ppm) δ = 8.19–8.15 (m, 1H), 7.99–7.94 (m, 1H), 7.85–7.79 (m, 2H), 6.75 (s, 1H), 3.80 (br.s, 2H).

^{13}C NMR (100 MHz, Acetonitrile-d_3 [1.3 ppm], ppm) δ = 137.0 (C_q), 134.3 (*C*H), 133.9 (*C*H), 133.7 (*C*H), 129.5 (*C*H, q, J = 6.5 Hz), 128.5 (C_q, q, J = 32.7 Hz), 124.1 (C_q, q, J = 273.2 Hz).

^{19}F NMR (376 MHz, ppm) δ = – 58.3.

MS (ESI+, DCM) m/z (%): 241 (25) $[M + H]^+$, 225 (63), 213 (100), 211 (93), 189 (49), 147 (46). Unknown adducts: 323 (19), 314 (16), 286 (16), 281 (15), 274 (20).

HRMS (ESI+, DCM) m/z (%) calcd for $C_7H_8F_3N_2O_2S$ $[M + H]^+$: 241.0253; found 241.0251.

IR (ATR, ṽ) = 3373 (w), 3274 (w), 3216 (w), 3108 (vw), 3087 (vw), 1618 (w), 1594 (vw), 1443 (w), 1340 (vs), 1305 (vs), 1295 (s), 1266 (vs), 1159 (vs), 1136 (vs), 1115 (vs), 1095 (vs), 1035 (vs), 1007 (w), 966 (w), 936 (m), 888 (m), 775 (vs), 744 (w), 713 (vs), 654 (m), 643 (s), 588 (vs), 562 (vs), 507 (s), 483 (s), 456 (s), 429 (vs), 390 (vs) cm^{-1}.

The analytical data are consistent with the literature.[5]

Additional information on the experimental procedure is available via the repository Chemotion: https://dx.doi.org/10.14272/reaction/SA-FUHFF-UHFFFADPSC-ISSXDMNIXW-UHFFFADPSC-NUHFF-NUHFF-NUHFF-ZZZ

Additional information on the characterization of the target compound is available via the repository Chemotion: https://dx.doi.org/10.14272/ISSXDMNIXWMHRB-UHFFFAOYSA-N.1

(*rac*)-*N*'-([2.2]Paracyclophanyl-1²-ylmethylene)-2-(trifluoromethyl)benzohydrazide (7)

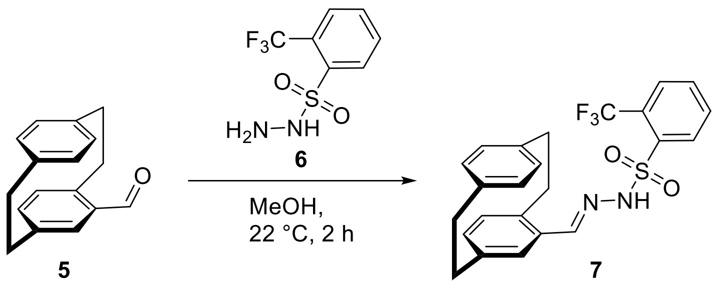

A 100 mL round-bottom flask equipped with a stirring bar was charged with (*rac*)-4-formyl[2.2]paracyclophane (2.36 g, 10.0 mmol, 1.00 equiv) (**5**), 2-(trifluoromethyl)benzenesulfonylhydrazide (2.40 g, 10.0 mmol, 1.00 equiv) (**6**), and MeOH (35 mL) and the mixture was stirred vigorously for 2 h at 22 °C. The precipitate was collected, washed with petroleum ether (5 × 20 mL), dried under suction for 1 h and then dried under high vacuum to obtain the target compound (*rac*)-*N*'-([2.2]paracyclophanyl-1²-ylmethylene)-2-(trifluoromethyl)benzohydrazide (**7**) as a pale yellow solid in 73% yield (3.33 g, 7.26 mmol).

Decomp. at 120–125 °C

R_f-value: 0.22 (DCM)

^{1}H NMR (400 MHz, Chloroform-*d* [7.26 ppm], ppm) δ = 8.53 (d, *J* = 7.8 Hz, 1H), 7.94–7.90 (m, 2H), 7.83–7.79 (m, 1H), 7.74–7.70 (m, 2H), 6.68 (d, *J* = 1.8 Hz, 1H), 6.55–6.52 (m, 1H), 6.45–6.42 (m, 3H), 6.09 (dd, *J* = 7.9 Hz, *J* = 1.4 Hz, 1H), 5.88 (dd, *J* = 7.8 Hz, *J* = 1.4 Hz, 1H), 3.49 (ddd, *J* = 11.5 Hz, *J* = 10.0 Hz, *J* = 1.3 Hz, 1H), 3.10–2.93 (m, 5H), 2.82–2.75 (m, 1H), 2.58 (ddd, *J* = 13.1 Hz, *J* = 10.0 Hz, *J* = 6.9 Hz, 1H).

^{13}C NMR (100 MHz, Chloroform-*d* [77.2 ppm], ppm) δ = 148.4 (*C*H), 140.4 (C_q), 140.0 (C_q), 139.5 (C_q), 139.1 (C_q), 137.1 (C_q), 135.6 (*C*H), 134.7 (*C*H), 133.9 (*C*H), 133.7 (*C*H), 133.3 (*C*H), 133.0 (*C*H), 132.7 (*C*H, 2C), 132.6 (*C*H), 131.9 (*C*H), 130.5 (*C*H), 128.6 (q, *J* = 5.8 Hz, *C*H), 127.9 (q, *J* = 30.5 Hz, C_q), 123.1 (q, *J* = 273.9 Hz, C_q), 35.4 (CH_2), 35.1 (CH_2), 34.8 (CH_2), 34.1 (CH_2).

^{19}F NMR (376 MHz, ppm) δ = – 58.09.

MS (ESI+, DCM) m/z (%): 459 (100) $[M + H]^+$.

HRMS (ESI+, DCM) m/z (%) calcd for $C_{24}H_{22}F_3N_2O_2S$ $[M + H]^+$: 459.1348; found 459.1343.

IR (ATR, ṽ) = 3262 (vw), 2929 (vw), 2854 (vw), 1684 (vw), 1499 (vw), 1408 (vw), 1397 (w), 1374 (m), 1330 (w), 1310 (s), 1272 (m), 1180 (vs), 1169 (vs), 1142 (s), 1116 (s), 1096 (w), 1055 (w), 1035 (s), 955 (w), 918 (w), 902 (w), 891 (w), 874 (w), 799 (w), 786 (w), 769 (s), 722 (m), 713 (m), 645 (w), 598 (s), 579 (vs), 497 (m), 449 (w), 384 (w) cm^{-1}.

The analytical data are consistent with the literature.[4]

Additional information on the experimental procedure is available via the repository Chemotion: https://dx.doi.org/10.14272/reaction/SA-FUHFF-UHFFFADPSC-XUUMNWRCVW-UHFFFADPSC-NUHFF-NJATD-NUHFF-ZZZ

Additional information on the characterization of the target compound is available via the repository Chemotion: https://dx.doi.org/10.14272/XUUMNWRCVWJMHB-LQKURTRISA-N.1

General procedure A for cyclopropenation using pCp-carbene precursor 7

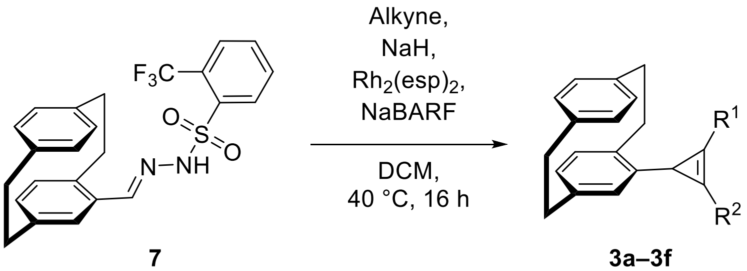

A 30 mL vial equipped with a stirring bar was charged with (*rac*)-*N*'-([2.2]paracyclophanyl-1^2-ylmethylene)-2-(trifluoromethyl)benzohydrazide (275 mg, 600 µmol, 1.00 equiv) (**7**), alkyne (2.2 equiv), sodium hydride (96.0 mg, 2.40 mmol, 4.00 equiv, 60% wt in mineral oil), $Rh_2(esp)_2$ (22.8 mg, 30.0 µmol, 5 mol%), sodium tetrakis[3,5-bis(trifluoromethyl)phenyl]borate (26.6 mg, 30.0 µmol, 5 mol%) and anhydrous DCM (12 mL) under an argon atmosphere. The reaction was stirred at 40 °C for 16 h. Then, the solvent was removed under reduced pressure, the residue was adsorbed on a small amount of Celite and was purified *via* flash-chromatography as indicated for each compound.

(*rac*)-1^2-(2,3-Dimethylcycloprop-2-en-1-yl)-[2.2]paracyclophane (3b)

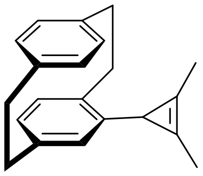

Following the general procedure with a slight modification employing (*rac*)-*N*'-([2.2]paracyclophanyl-1^2-ylmethylene)-2-(trifluoromethyl)benzohydrazide (275 mg, 600 µmol, 1.00 equiv) (**7**), but-2-yne (286 mg, 413 µL, 5.28 mmol, 8.80 equiv), sodium hydride (96.0 mg, 2.40 mmol, 4.00 equiv, 60% wt in mineral oil), $Rh_2(esp)_2$ (22.8 mg, 30.0 µmol, 5 mol%) and sodium tetrakis[3,5-bis(trifluoromethyl)phenyl]borate (26.6 mg, 30.0 µmol, 5 mol%). The crude was purified *via* flash-chromatography (Biotage Isolera One) on silica gel (puriFlash F0025) using *n*-hexane (7 column volumes; 1 column volume = 30 mL; flow: 25 mL/min). The target compound (*rac*)-1^2-(2,3-dimethylcycloprop-2-en-1-yl)-[2.2]paracyclophane (**3b**) was isolated as a colorless solid in 58% yield (94.7 mg, 345 µmol).

Melting point range: 85–86 °C

R_f-value: 0.16 (cHex)

^{1}H NMR (400 MHz, Dichloromethane-d_2 [5.32 ppm], ppm) δ = 6.79 (dd, *J* = 7.8 Hz, *J* = 1.3 Hz, 1H), 6.53–6.48 (m, 2H), 6.38 (dd, *J* = 7.6 Hz, *J* = 1.7 Hz, 1H), 6.34–6.31 (m, 1H), 6.28 (dd, *J* = 7.6 Hz, *J* = 1.6 Hz, 1H), 5.74 (d, *J* = 1.6 Hz, 1H), 3.62 (ddd, *J* = 13.1 Hz, *J* = 9.9 Hz, *J* = 1.6 Hz, 1H), 3.19–3.13 (m, 1H), 3.09–2.93 (m, 4H), 2.89–2.76 (m, 2H), 2.44 (s, 1H), 2.34 (q, *J* = 1.4 Hz, 3H), 1.88 (q, *J* = 1.4 Hz, 3H).

^{13}C NMR (100 MHz, Dichloromethane-d_2 [53.8 ppm], ppm) δ = 147.1 (C_q), 140.3 (C_q), 139.9 (C_q), 139.6 (C_q), 138.2 (C_q), 134.9 (CH), 133.7 (CH), 133.1 (CH), 132.2 (CH), 130.5 (CH), 129.9 (CH), 128.9 (CH), 110.0 (C_q), 104.1 (C_q), 35.8 (CH_2), 35.6 (CH_2), 35.2 (CH_2), 33.9 (CH_2), 22.7 (CH), 9.8 (CH_3), 9.2 (CH_3).

MS (ESI+, DCM) m/z (%): 275 (31) $[M + H]^+$, 221 (100), 102 (99).

HRMS (ESI+, DCM) m/z (%) calcd for $C_{21}H_{23}$ $[M + H]^+$: 275.1795; found 275.1785.

IR (ATR, ṽ) = 2951 (m), 2924 (s), 2849 (m), 1881 (w), 1592 (w), 1502 (w), 1482 (w), 1432 (s), 1414 (m), 1366 (w), 1347 (w), 1239 (w), 1203 (w), 1180 (w), 1153 (m), 1136 (m), 1098 (m), 1068 (w), 1031 (w), 984 (m), 933 (w), 901 (m), 868 (m), 795 (vs), 762 (w), 742 (m), 714 (vs), 677 (s), 642 (m), 596 (vs), 557 (w), 509 (vs), 477 (w), 466 (w), 426 (w), 385 (w) cm^{-1}.

Additional information on the experimental procedure is available via the repository Chemotion: https://dx.doi.org/10.14272/reaction/SA-FUHFF-UHFFFADPSC-PWDVDRMYMF-UHFFFADPSC-NUHFF-NUHFF-NUHFF-ZZZ

Additional information on the characterization of the target compound is available via the repository Chemotion: https://dx.doi.org/10.14272/PWDVDRMYMFXQOH-UHFFFAOYSA-N.1

(*rac*)-1^2-(2,3-Diethylcycloprop-2-en-1-yl)-[2.2]paracyclophane (3c)

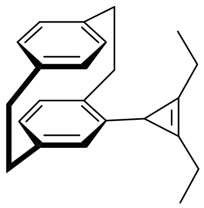

Following the general procedure **A** employing (*rac*)-*N*'-([2.2]paracyclophanyl-1^2-ylmethylene)-2-(trifluoromethyl)benzohydrazide (275 mg, 600 µmol, 1.00 equiv) (**7**), hex-3-yne (108 mg, 150 µL, 1.32 mmol, 2.20 equiv), sodium hydride (96.0 mg, 2.40 mmol, 4.00 equiv, 60% wt in mineral oil), $Rh_2(esp)_2$ (22.8 mg, 30.0 µmol, 5 mol%) and sodium tetrakis[3,5-bis(trifluoromethyl)phenyl]borate (26.6 mg, 30.0 µmol, 5 mol%). The crude was purified *via* flash-chromatography (Büchi Pure C-815 Flash) on silica gel (Büchi 140000023) using *n*-hexane (5 column volumes; 1 column volume = 30 mL; flow: 32 mL/min). The target compound (*rac*)-1^2-(2,3-diethylcycloprop-2-en-1-yl)-[2.2]paracyclophane (**3c**) was isolated as a colorless solid in 54% yield (98.5 mg, 326 µmol).

Melting point range: 130–131 °C

R_f-value: 0.19 (cHex)

^{1}H NMR (400 MHz, Dichloromethane-d_2 [5.32 ppm], ppm) δ = 6.77 (dd, J = 7.8 Hz, J = 1.6 Hz, 1H), 6.52–6.47 (m, 2H), 6.36 (dd, J = 7.8 Hz, J = 1.5 Hz, 1H), 6.31–6.29 (m, 1H), 6.25 (dd, J = 7.6 Hz, J = 1.8 Hz, 1H), 5.76 (d, J = 1.8 Hz, 1H), 3.61 (ddd, J = 13.2 Hz, J = 9.8 Hz, J = 1.7 Hz, 1H), 3.17–3.08 (m, 1H), 3.03 (ddd, J = 9.8 Hz, J = 6.4 Hz, J = 3.1 Hz, 3H), 2.99–2.91 (m, 1H), 2.87–2.75 (m, 3H), 2.71–2.60 (m, 1H), 2.53 (s, 1H), 2.30–2.23 (m, 2H), 1.38 (t, J = 7.4 Hz, 3H), 1.03 (t, J = 7.4 Hz, 3H).

^{13}C NMR (100 MHz, Dichloromethane-d_2 [53.8 ppm], ppm) δ = 147.4 (C_q), 140.3 (C_q), 139.8 (C_q), 139.7 (C_q), 138.0 (C_q), 134.8 (CH), 133.7 (CH), 133.1 (CH), 132.2 (CH), 130.3 (CH), 130.1 (CH), 128.8 (CH), 115.2 (C_q), 108.8 (C_q), 35.8 (CH_2), 35.6 (CH_2), 35.2 (CH_2), 33.8 (CH_2), 22.7 (CH), 19.0 (CH_2), 18.4 (CH_2), 13.1 (CH_3), 12.6 (CH_3).

MS (ESI+, DCM) m/z (%): 303 (100) [M + H]$^+$, 120 (62). Unknown adduct: 392 (57).

HRMS (ESI+, DCM) m/z (%) calcd for $C_{23}H_{27}$ [M + H]$^+$: 303.2108; found 303.2106.

IR (ATR, ṽ) = 2962 (s), 2953 (s), 2928 (vs), 2894 (m), 2868 (m), 2850 (m), 1870 (m), 1591 (m), 1500 (w), 1483 (w), 1451 (m), 1429 (m), 1414 (s), 1371 (w), 1322 (w), 1146 (w), 1119 (w), 1096 (m), 1078 (w), 993 (vs), 933 (w), 899 (m), 868 (s), 796 (vs), 752 (m), 737 (m), 714 (vs), 662 (vs), 608 (vs), 507 (vs), 467 (w) cm^{-1}.

Additional information on the experimental procedure is available via the repository Chemotion: https://dx.doi.org/10.14272/reaction/SA-FUHFF-UHFFFADPSC-NQMGUYIZDF-UHFFFADPSC-NUHFF-NUHFF-NUHFF-ZZZ

Additional information on the characterization of the target compound is available via the repository Chemotion: https://dx.doi.org/10.14272/NQMGUYIZDFQTKU-UHFFFAOYSA-N.1

(*rac*)-1^2-(2,3-Dipropylcycloprop-2-en-1-yl)-[2.2]paracyclophane (3a)

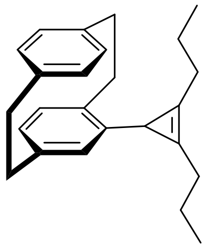

Following the general procedure **A** employing (*rac*)-*N*'-([2.2]paracyclophanyl-1^2-ylmethylene)-2-(trifluoromethyl)benzohydrazide (275 mg, 600 µmol, 1.00 equiv) (**7**), oct-3-yne (145 mg, 193 µL, 1.32 mmol, 2.20 equiv), sodium hydride (96.0 mg, 2.40 mmol, 4.00 equiv, 60% wt in mineral oil), $Rh_2(esp)_2$ (22.8 mg, 30.0 µmol, 5 mol%) and sodium tetrakis[3,5-bis(trifluoromethyl)phenyl]borate (26.6 mg, 30.0 µmol, 5 mol%). The crude was purified *via* flash-chromatography (Büchi Pure C-815 Flash) on silica gel (Büchi 140000023) using *n*-hexane (5 column volumes; 1 column volume = 30 mL; flow: 32 mL/min). The target compound (*rac*)-1^2-(2,3-dipropylcycloprop-2-en-1-yl)-[2.2]paracyclophane (**3a**) was isolated as a colorless solid in 54% yield (108 mg, 327 µmol).

Melting point range: 80–82 °C

R_f-value: 0.32 (cHex)

^{1}H NMR (400 MHz, Dichloromethane-d_2 [5.32 ppm], ppm) δ = 6.77 (dd, *J* = 7.8 Hz, *J* = 1.6 Hz, 1H), 6.52–6.46 (m, 2H), 6.36 (dd, *J* = 7.8 Hz, *J* = 1.5 Hz, 1H), 6.31–6.24 (m, 2H), 5.77 (d, *J* = 1.8 Hz, 1H), 3.61 (ddd, *J* = 13.3 Hz, *J* = 9.8 Hz, *J* = 1.8 Hz, 1H), 3.17–3.08 (m, 1H), 3.06–2.91 (m, 4H), 2.87–2.73 (m, 3H), 2.70–2.61 (m, 1H), 2.49 (s, 1H), 2.26–2.21 (m, 2H), 1.91–1.74 (m, 2H), 1.48–1.36 (m, 2H), 1.12 (t, *J* = 7.4 Hz, 3H), 0.85 (t, *J* = 7.4 Hz, 3H).

^{13}C NMR (100 MHz, Dichloromethane-d_2 [53.8 ppm], ppm) δ = 147.5 (C_q), 140.3 (C_q), 139.8 (C_q), 139.7 (C_q), 138.0 (C_q), 134.8 (CH), 133.7 (CH), 133.1 (CH), 132.3 (CH), 130.4 (CH), 130.1 (CH), 128.8 (CH), 114.2 (C_q), 107.6 (C_q), 35.8 (CH$_2$), 35.7 (CH$_2$), 35.2 (CH$_2$), 33.8 (CH$_2$), 27.6 (CH$_2$), 26.9 (CH$_2$), 22.3 (CH), 21.8 (CH$_2$), 21.2 (CH$_2$), 14.5 (CH$_3$), 14.2 (CH$_3$).

MS (ESI+, DCM) m/z (%): 331 (42) [M + H]$^+$, 237 (100).

HRMS (ESI+, DCM) m/z (%) calcd for $C_{25}H_{31}$ [M + H]$^+$: 331.2421; found 331.2412.

IR (ATR, ṽ) = 2949 (vs), 2924 (vs), 2894 (s), 2864 (s), 2850 (s), 1871 (w), 1591 (w), 1482 (w), 1453 (m), 1438 (w), 1422 (w), 1412 (s), 1375 (w), 1146 (w), 1129 (w), 1101 (w), 1086 (w), 994 (s), 932 (w), 899 (m), 868 (s), 795 (vs), 756 (w), 738 (m), 714 (vs), 659 (vs), 606 (vs), 506 (vs) cm^{-1}.

Additional information on the experimental procedure is available via the repository Chemotion: https://dx.doi.org/10.14272/reaction/SA-FUHFF-UHFFFADPSC-NQMGUYIZDF-UHFFFADPSC-NUHFF-NUHFF-NUHFF-ZZZ

Additional information on the characterization of the target compound is available via the repository Chemotion: https://dx.doi.org/10.14272/NQMGUYIZDFQTKU-UHFFFAOYSA-N.1

(*rac*)-1^2-(2,3-Dibutylcycloprop-2-en-1-yl)-[2.2]paracyclophane (3d)

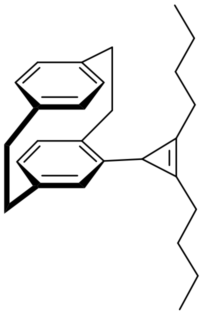

Following the general procedure **A** employing (*rac*)-*N*'-([2.2]paracyclophanyl-1^2-ylmethylene)-2-(trifluoromethyl)benzohydrazide (275 mg, 600 µmol, 1.00 equiv) (**7**), 5-decyne (182 mg, 237 µL, 1.32 mmol, 2.20 equiv), sodium hydride (96.0 mg, 2.40 mmol, 4.00 equiv, 60% wt in mineral oil), $Rh_2(esp)_2$ (22.8 mg, 30.0 µmol, 5 mol%) and sodium tetrakis[3,5-bis(trifluoromethyl)phenyl]borate (26.6 mg, 30.0 µmol, 5 mol%). The crude was purified *via* flash-chromatography (Büchi Pure C-815 Flash) on silica gel (Büchi 140000024) using pentane(5 column volumes; 1 column volume = 50 mL; flow: 50 mL/min). The target compound (*rac*)-1^2-(2,3-dibutylcycloprop-2-en-1-yl)-[2.2]paracyclophane (**3d**) was isolated as a yellow oil in 41% yield (88.0 mg, 245 µmol).

R_f-value: 0.87 (cHex)

^{1}H NMR (400 MHz, Chloroform-*d* [7.26 ppm], ppm) δ = 6.78 (dd, *J* = 7.8 Hz, *J* = 1.4 Hz, 1H), 6.56–6.48 (m, 2H), 6.38–6.27 (m, 3H), 5.77 (d, *J* = 1.6 Hz, 1H), 3.63 (dd, *J* = 9.9 Hz, *J* = 1.5 Hz, 1H), 3.20–3.14 (m, 1H), 3.10–2.93 (m, 4H), 2.89–2.75 (m, 3H), 2.70–2.62 (m, 1H), 2.49 (s, 1H), 2.33–2.19 (m, 2H), 1.82–1.74 (m, 2H), 1.60–1.49 (m, 2H), 1.44–1.36 (m, 2H), 1.33–1.22 (m, 2H), 1.05 (t, *J* = 7.3 Hz, 3H), 0.84 (t, *J* = 7.3 Hz, 3H).

^{13}C NMR (100 MHz, Chloroform-*d* [77.2 ppm], ppm) δ = 147.3 (C_q), 139.9 (C_q), 139.4 (C_q), 139.3 (C_q), 137.7 (C_q), 134.6 (*C*H), 133.4 (*C*H), 132.8 (*C*H), 132.0 (*C*H), 130.2 (*C*H), 129.8 (*C*H), 128.5 (*C*H), 114.0 (C_q), 107.1 (C_q), 35.7 (CH_2), 35.5 (CH_2), 35.1 (CH_2), 33.7 (CH_2), 30.3 (CH_2), 29.7 (CH_2), 25.0 (CH_2), 24.3 (CH_2), 22.8 (CH_2), 22.6 (CH_2), 22.1 (*C*H), 14.1 (CH_3), 13.9 (CH_3).

MS (ESI+, DCM), m/z (%): 358 (8) [M]$^+$, 357 (46). Unknown adducts: 407 (5), 405 (16), 372 (24), 371 (100).

HRMS (ESI+, DCM), m/z (%) calcd for $C_{27}H_{34}^+$ [M]$^+$: 358.2655, found 358.2603.

IR (ATR, ṽ) = 2952 (vs), 2925 (vs), 2870 (s), 2853 (s), 1871 (w), 1592 (m), 1557 (w), 1500 (m), 1483 (m), 1455 (s), 1435 (s), 1412 (s), 1377 (m), 1319 (m), 1239 (m), 1203 (m), 1180 (m), 1153 (m), 1129 (m), 1088 (m), 1044 (m), 999 (s), 929 (s), 898 (s), 866 (s), 795 (vs), 762 (s), 738 (s), 715 (vs), 680 (m), 656 (s), 605 (s), 575 (m), 557 (m), 540 (m), 509 (s), 467 (m), 442 (w), 429 (w), 401 (w), 375 (w) cm^{-1}.

Additional information on the experimental procedure is available via the repository Chemotion: https://dx.doi.org/10.14272/reaction/SA-FUHFF-UHFFFADPSC-YVPBHOCIEH-UHFFFADPSC-NUHFF-NUHFF-NUHFF-ZZZ

Additional information on the characterization of the target compound is available via the repository Chemotion: https://dx.doi.org/10.14272/YVPBHOCIEHQVGE-UHFFFAOYSA-N.1

(*rac*)-1^2-(2-(*tert*-Butyl)cycloprop-2-en-1-yl)-[2.2]paracyclophane (3e)

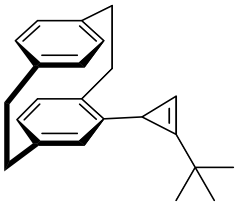

Following the general procedure **A** employing (*rac*)-*N*'-([2.2]paracyclophanyl-1^2-ylmethylene)-2-(trifluoromethyl)benzohydrazide (275 mg, 600 µmol, 1.00 equiv) (**7**), 3,3-dimethylbut-1-yne (108 mg, 163 µL, 1.32 mmol, 2.20 equiv), sodium hydride (96.0 mg, 2.40 mmol, 4.00 equiv, 60% wt in mineral oil), $Rh_2(esp)_2$ (22.8 mg, 30.0 µmol, 5 mol%) and sodium tetrakis[3,5-bis(trifluoromethyl)phenyl]borate (26.6 mg, 30.0 µmol, 5 mol%). The crude was purified *via* flash-chromatography (Büchi Pure C-815 Flash) on silica gel (Büchi 140000024) using pentane (5 column volumes; 1 column volume = 50 mL; flow: 50 mL/min). The target compound (*rac*)-1^2-(2-(*tert*-butyl)cycloprop-2-en-1-yl)-[2.2]paracyclophane (**3e**) was isolated as a colorless solid in 45% yield (81.8 mg, 270 µmol) with a diastereomeric ratio of 6:1.

R_f-value: 0.32 (cHex)

^{1}H NMR (400 MHz, Dichloromethane-d_2 [5.32 ppm], ppm) δ = 6.83 (dd, *J* = 7.8 Hz, *J* = 1.6 Hz, 0.86H, isomer1), 6.79 (dd, *J* = 8.0 Hz, *J* = 1.1 Hz, 0.14H, isomer2), 6.63 (d, *J* = 1.5 Hz, 0.14H, isomer2), 6.55 (d, *J* = 1.8 Hz, 0.86H, isomer1), 6.50–6.45 (m, 1.72H, isomer1), 6.44 (m, 0.28H, isomer2), 6.33–6.31 (m, 1.72H, isomer1), 6.30–6.29 (m, 0.28H, isomer2), 6.13 (d, *J* = 0.9 Hz, 0.14H, isomer2), 5.83 (d, *J* = 0.8 Hz, 0.86H, isomer1), 3.65 (ddd, *J* = 13.3 Hz, *J* = 9.9 Hz, *J* = 1.8 Hz, 0.86H, isomer1), 3.47 (ddd, *J* = 13.4 Hz, *J* = 9.9 Hz, *J* = 1.4 Hz, 0.14H, isomer2), 3.18–3.12 (m, 1H, isomer1 + isomer2), 3.07–2.76 (m, 7H, isomer1 + isomer2), 2.65 (d, *J* = 1.8 Hz, 0.86H, isomer1), 2.63 (d, *J* = 1.3 Hz, 0.14H, isomer2), 1.21 (s, 1.3H, isomer2), 1.01 (s, 7.7H, isomer1).

Due to low peak intensities, only the signals of the diastereomer 1 could be analyzed:

^{13}C NMR (100 MHz, Dichloromethane-d_2 [53.8 ppm], ppm) δ = 146.5 (C_q), 140.1 (C_q), 139.7 (C_q), 139.7 (C_q), 138.1 (C_q), 134.6 (*C*H), 133.5 (*C*H), 133.2 (*C*H), 132.4 (*C*H), 130.2 (*C*H), 130.2 (*C*H), 129.2 (*C*H), 93.6 (*C*H), 35.7 (CH_2), 35.6 (CH_2), 35.0 (CH_2), 33.8 (CH_2), 31.8 (C_q), 28.8 (C_q), 28.4 (s, 3C), 20.8 (*C*H).

MS (ESI+, DCM) m/z (%): 303 (100) [M + H]$^+$.

HRMS (ESI+, DCM) m/z (%) calcd for $C_{23}H_{27}^+$ [M + H]$^+$: 303.2108, found 303.2108.

IR (ATR, ṽ) = 3010 (vw), 2955 (s), 2924 (s), 2894 (m), 2851 (m), 1768 (w), 1592 (w), 1499 (w), 1485 (w), 1472 (w), 1455 (m), 1438 (w), 1412 (w), 1388 (w), 1360 (m), 1334 (vw), 1242 (w), 1215 (w), 1203 (w), 1181 (w), 1154 (w), 1119 (w), 1095 (w), 1068 (vw), 1024 (vw), 1009 (m), 976 (vw), 956 (m), 933 (w), 901 (m), 868 (m), 796 (vs), 759 (w), 737 (w), 711 (vs), 662 (m), 623 (vs), 589 (w), 558 (w), 510 (vs), 494 (s), 449 (w), 428 (w), 419 (w), 388 (w), 381 (w) cm^{-1}.

Additional information on the experimental procedure is available via the repository Chemotion: https://dx.doi.org/10.14272/reaction/SA-FUHFF-UHFFFADPSC-FKSBFEVPBX-UHFFFADPSC-NUHFF-NUHFF-NUHFF-ZZZ

Additional information on the characterization of the target compound is available via the repository Chemotion: https://dx.doi.org/10.14272/FKSBFEVPBXWWSQ-UHFFFAOYSA-N.1

(*rac*)-1^2-(2-Ethyl-3-methylcycloprop-2-en-1-yl)-[2.2]paracyclophane (3f)

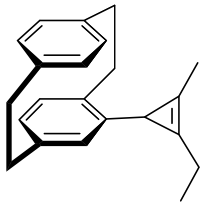

Following the general procedure **A** employing (*rac*)-*N*'-([2.2]paracyclophanyl-1^2-ylmethylene)-2-(trifluoromethyl)benzohydrazide (275 mg, 600 µmol, 1.00 equiv) (**7**), 2-pentyne (89.9 mg, 127 µL, 1.32 mmol, 2.20 equiv), sodium hydride (96.0 mg, 2.40 mmol, 4.00 equiv, 60% wt in mineral oil), $Rh_2(esp)_2$ (22.8 mg, 30.0 µmol, 5 mol%) and sodium tetrakis[3,5-bis(trifluoromethyl)phenyl]borate (26.6 mg, 30.0 µmol, 5 mol%). The crude was purified *via* flash-chromatography (Biotage Isolera One) on silica gel (puriFlash F0025) using *n*-hexane (7 column volumes; 1 column volume = 30 mL; flow: 25 mL/min). The target compound (*rac*)-1^2-(2-ethyl-3-methylcycloprop-2-en-1-yl)-[2.2]paracyclophane (**3f**) was isolated as a colorless solid in 47% yield (80.5 mg, 279 µmol) with a diastereomeric ratio of 3:2.

R_f-value: 0.75 (cHex/EtOAc 10:1)

^{1}H NMR (400 MHz, Chloroform-*d* [7.26 ppm], ppm) δ = 6.82–6.77 (m, 1H, isomer1 + isomer2), 6.55–6.50 (m, 2H, isomer1 + isomer2), 6.41–6.34 (m, 2H, isomer1 + isomer2), 6.31–6.29 (m, 1H, isomer1 + isomer2), 5.77–5.76 (m, 1H, isomer1 + isomer2), 3.65 (ddd, *J* = 11.6 Hz, *J* = 9.8 Hz, *J* = 1.2 Hz, 1H, isomer1 + isomer2), 3.22–3.15 (m, 1H, isomer1 + isomer2), 3.12–2.96 (m, 4H, isomer1 + isomer2), 2.91–2.77 (m, 2H, isomer1 + isomer2), 2.71–2.61 (m, 0.8H, isomer2), 2.52 (s, 0.6H, isomer1), 2.50 (s, 0.4H, isomer2), 2.37 (t, *J* = 1.5 Hz, 1.2H, isomer2), 2.34–2.22 (m, 1.2H, isomer1), 1.92 (t, *J* = 1.6 Hz, 1.8H, isomer1), 1.41 (t, *J* = 7.4 Hz, 1.8H, isomer1), 1.05 (t, *J* = 7.4 Hz, 1.2H, isomer2).

^{13}C NMR (100 MHz, Chloroform-*d* [77.2 ppm], ppm) δ = 147.2 (C_q, 0.4C, isomer2), 146.8 (C_q, 0.6C, isomer1), 139.9 (C_q, 1C, isomer1 + isomer2), 139.5 (C_q, 0.6C, isomer1), 139.5 (C_q, 0.4C, isomer2), 139.3 (C_q, 1C, isomer1 + isomer2), 137.8 (C_q, 0.6C, isomer1), 137.7 (C_q, 0.4C, isomer2), 134.7 (*C*H, 0.6C, isomer1), 134.6 (*C*H, 0.4C, isomer2), 133.4 (*C*H, 0.4C, isomer2), 133.4 (*C*H, 0.6C, isomer1), 132.9 (*C*H, 0.4C, isomer2), 132.8 (*C*H, 0.6C, isomer1), 132.0 (*C*H, 0.6C, isomer1), 131.9 (*C*H, 0.4C, isomer2), 130.2 (*C*H, 0.4C, isomer2), 130.1 (*C*H, 0.6C, isomer1), 129.8 (*C*H, 0.6C, isomer1), 129.7 (*C*H, 0.4C, isomer2), 128.6 (*C*H, 0.6C, isomer1), 128.5 (*C*H, 0.4C, isomer2), 115.3 (C_q, 0.4C, isomer2), 109.7 (C_q, 0.6C, isomer1), 109.3 (C_q, 0.6C, isomer1), 102.6 (C_q, 0.4C, isomer2), 35.6 (CH_2, 1C, isomer1 + isomer2), 35.5 (CH_2, 1C, isomer1 + isomer2), 35.1 (CH_2, 1C, isomer1 + isomer2), 33.7 (CH_2, 0.4C, isomer2), 33.7 (CH_2, 0.6C, isomer1), 22.8 (*C*H, 0.6C, isomer1), 22.4 (*C*H, 0.4C, isomer2), 18.9 (CH_2, 0.6C, isomer1), 18.2 (CH_2, 0.4C, isomer2), 12.7 (CH_3, 0.6C, isomer1), 12.1 (CH_3, 0.4C, isomer2), 10.0 (CH_3, 0.4C, isomer2), 9.5 (CH_3, 0.6C, isomer1).

MS (ESI+, DCM) m/z (%): 289 (100) [M + H]$^+$, 221 (32), 193 (17), 147 (11). HRMS (ESI+, DCM) m/z (%) calcd for $C_{22}H_{25}^+$ [M + H]$^+$: 289.1951, found 289.1950.

IR (ATR, ṽ) = 3010 (w), 2948 (s), 2924 (vs), 2894 (s), 2871 (m), 2849 (s), 1880 (w), 1592 (m), 1557 (w), 1500 (w), 1483 (m), 1449 (m), 1435 (s), 1414 (s), 1370 (w), 1349 (w), 1319 (w), 1310 (w), 1238 (w), 1203 (w), 1180 (w), 1154 (m), 1132 (w), 1098 (m), 1058 (w), 1017 (w), 990 (s), 958 (w), 933 (w), 899 (s), 868 (s), 830 (w), 795 (vs), 756 (m), 741 (s), 714 (vs), 670 (m), 659 (s), 642 (w), 635 (w), 603 (vs), 591 (s), 560 (w), 510 (vs), 466 (w), 426 (w), 416 (w), 404 (w), 387 (w) cm^{-1}.

Additional information on the experimental procedure is available via the repository Chemotion: https://dx.doi.org/10.14272/reaction/SA-FUHFF-UHFFFADPSC-LPWYCCDKPW-UHFFFADPSC-NUHFF-NUHFF-NUHFF-ZZZ

Additional information on the characterization of the target compound is available via the repository Chemotion: https://dx.doi.org/10.14272/LPWYCCDKPWMPND-UHFFFAOYSA-N.1

General procedure B for cyclopropenium salt synthesis

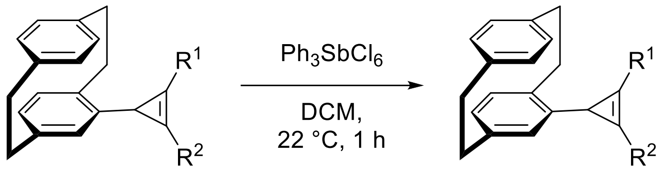

3a–3f **11a–11d**

A 20 mL Schlenk tube equipped with a stirring bar was charged with cyclopropene (1.00 equiv) and anhydrous DCM (10 mL). Triphenylcarbenium hexachloroantimonate (1.00 equiv) was added in one portion to give an orange solution. The mixture was stirred for 1 h, during which time the color gradually changed to green. Then, the volume of the solvent was reduced to about 4 mL under reduced pressure and diethyl ether (20 mL) was added. The precipitate was collected and dried under suction for 30 min.

(*rac*)-1^2-(2,3-Dimethylcycloprop-2-en-1-yl)-[2.2]paracyclophane hexachloroantimonate (11a)

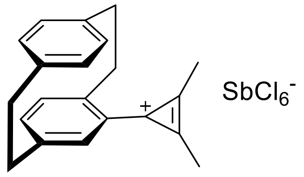

Following the general procedure **B** employing (*rac*)-1^2-(2,3-dimethylcycloprop-2-en-1-yl)-[2.2]paracyclophane (110 mg, 401 µmol, 1.00 equiv) (**3b**) and triphenylcarbenium hexachloroantimonate (232 mg, 401 µmol, 1.00 equiv). The target compound *(rac*)-1^2-(2,3-Dimethylcycloprop-2-en-1-yl)-[2.2]paracyclophane hexachloroantimonate (**11a**) was isolated as a yellow solid in 93% yield (227 mg, 373 µmol).

Decomp. at 100–103 °C

^{1}H NMR (400 MHz, Acetonitrile-*d*$_3$ [1.94 ppm], ppm) δ = 7.47 (d, *J* = 1.8 Hz, 1H), 7.10 (dd, *J* = 7.9 Hz, *J* = 1.8 Hz, 1H), 6.92 (d, *J* = 7.9 Hz, 1H), 6.67 (qd, *J* = 7.9 Hz, *J* = 1.8 Hz, 2H), 6.46 (dd, *J* = 7.9 Hz, *J* = 1.6 Hz, 1H), 6.18 (dd, *J* = 7.9 Hz, *J* = 1.8 Hz, 1H), 3.66–3.59 (m, 1H), 3.43–3.11 (m, 6H), 3.04 (s, 6H), 3.00–2.93 (m, 1H).

^{13}C NMR (100 MHz, Acetonitrile-*d*$_3$ [118.3 ppm], ppm) δ = 170.2 (C_q, 2C), 165.8 (C_q), 149.5 (C_q), 144.5 (*C*H), 143.8 (C_q), 141.5 (*C*H), 141.2 (C_q), 139.7 (C_q), 137.3 (*C*H), 134.4 (*C*H), 134.0 (*C*H), 133.6 (*C*H), 133.4 (*C*H), 121.5 (C_q), 36.0 (CH_2), 35.6 (CH_2), 35.1 (CH_2), 34.8 (CH_2), 13.7 (CH_3, 2C).

MS (ESI+, DCM) m/z (%): 273 (100) $[M]^+$. HRMS (ESI+, DCM) m/z (%) calcd for $C_{21}H_{21}^+$ [M $]^+$: 273.1638, found 273.1639.

IR (ATR, ṽ) = 2962 (w), 2938 (w), 2921 (w), 2897 (w), 2857 (w), 1853 (w), 1591 (w), 1548 (w), 1489 (m), 1451 (vs), 1411 (vs), 1381 (vs), 1353 (w), 1320 (w), 1302 (w), 1203 (w), 1184 (w), 1160 (w), 1136 (w), 1028 (w), 983 (w), 962 (w), 948 (w), 916 (w), 905 (w), 881 (w), 866 (w), 853 (w), 796 (m), 724 (s), 676 (w), 647 (w), 608 (s), 510 (s) cm^{-1}.

UV/VIS (absorption, DCM, 22 °C), λ_{max} = 327, 263 nm.

Additional information on the experimental procedure is available via the repository Chemotion: https://dx.doi.org/10.14272/reaction/SA-FUHFF-UHFFFADPSC-VMJWTLFNGA-UHFFFADPSC-NUHFF-HUHFF-NUHFF-ZZZ

Additional information on the characterization of the target compound is available via the repository Chemotion: https://dx.doi.org/10.14272/VMJWTLFNGANXEL-UHFFFAOYSA-H.1

(rac)-1²-(2,3-Dipropylcycloprop-2-en-1-yl)ium-[2.2]paracyclophane hexachloroantimonate (11b)

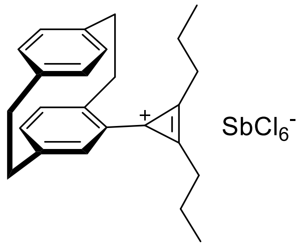

Following the general procedure **B** employing (*rac*)-1²-(2,3-dipropylcycloprop-2-en-1-yl)-[2.2]paracyclophane (65.0 mg, 197 µmol, 1.00 equiv) (**3a**) and triphenylcarbenium hexachloroantimonate (114 mg, 197, 1.00 equiv). The target compound (*rac*)-1²-(2,3-dipropylcycloprop-2-en-1-yl)ium-[2.2]paracyclophane hexachloroantimonate (**11b**) was isolated as a yellow solid in 88% yield (115 mg, 173 µmol).

Melting point range: 138–140 °C

^{1}H NMR (400 MHz, Dichloromethane-d_2 [5.32 ppm], ppm) δ = 7.34 (d, *J* = 1.6 Hz, 1H), 7.09 (dd, *J* = 7.9 Hz, *J* = 1.6 Hz, 1H), 6.90 (d, *J* = 7.9 Hz, 1H), 6.70 (qd, *J* = 7.9 Hz, *J* = 1.5 Hz, 2H), 6.44 (dd, *J* = 7.9 Hz, *J* = 1.3 Hz, 1H), 6.13 (dd, *J* = 7.9 Hz, *J* = 1.4 Hz, 1H), 3.57 (ddd, *J* = 13.1 Hz, *J* = 10.5 Hz, *J* = 2.9 Hz, 1H), 3.48 (t, *J* = 7.4 Hz, 4H), 3.44–3.13 (m, 6H), 3.02 (ddd, *J* = 13.2 Hz, *J* = 10.5 Hz, *J* = 4.4 Hz, 1H), 2.12 (h, *J* = 7.4 Hz, 4H), 1.22 (t, *J* = 7.4 Hz, 6H).

^{13}C NMR (100 MHz, Dichloromethane-d_2 [53.8 ppm], ppm) δ = 171.1 (C_q, 2C), 165.6 (C_q), 149.7 (C_q), 145.3 (*C*H), 143.8 (C_q), 140.8 (*C*H), 140.7 (C_q), 138.5 (C_q), 137.3 (*C*H), 133.8 (*C*H), 133.3 (*C*H), 133.1 (*C*H), 132.8 (*C*H), 120.0 (C_q), 36.2 (CH_2), 35.4 (CH_2), 35.2 (CH_2), 34.8 (CH_2), 30.3 (CH_2, 2C), 20.5 (CH_2, 2C), 14.3 (CH_3, 2C).

MS (ESI+, DCM) m/z (%): 329 (3) $[M - SbCl_6]^+$, 147 (100), 124 (5), 122 (33), 100 (4). Unknown adducts: 372 (4), 370 (4).

HRMS (ESI+, DCM) m/z (%) calcd for $C_{25}H_{29}^+$ $[M - SbCl_6]^+$: 329.2264; found 329.2237.

IR (ATR, ṽ) = 2963 (m), 2928 (w), 2895 (w), 2873 (w), 2856 (w), 1837 (w), 1592 (w), 1550 (w), 1490 (w), 1442 (vs), 1419 (s), 1378 (vs), 1319 (w), 1307 (w), 1283 (w), 1248 (w), 1224 (w), 1208 (w), 1187 (w), 1159 (w), 1111 (w), 1075 (w), 946 (w), 905 (w), 881 (w), 860 (w), 837 (w), 798 (w), 758 (w), 732 (m), 722 (s), 700 (w), 688 (w), 643 (w), 609 (m), 509 (m) cm^{-1}.

UV/VIS (absorption, DCM, 22 °C), λ_{max} = 325, 265 nm.

Additional information on the experimental procedure is available via the repository Chemotion: https://dx.doi.org/10.14272/reaction/SA-FUHFF-UHFFFADPSC-ZSEJEZICWY-UHFFFADPSC-NUHFF-HUHFF-NUHFF-ZZZ

Additional information on the characterization of the target compound is available via the repository Chemotion: https://dx.doi.org/10.14272/ZSEJEZICWYCUBO-UHFFFAOYSA-H.1

(rac)- 1²-(2,3-Dibutylcycloprop-2-en-1-yl)-[2.2]paracyclophane hexachloroantimonate (11c)

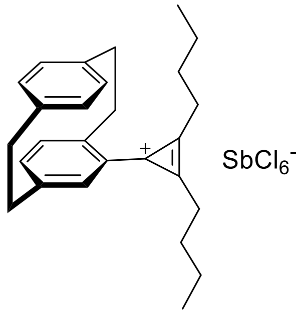

Following the general procedure **B** employing (*rac*)-1²-(2,3-dibutylcycloprop-2-en-1-yl)-[2.2]paracyclophane (22.0 mg, 61.4 µmol, 1.00 equiv) (**3d**) and triphenylcarbenium hexachloroantimonate (35.5 mg, 61.4 µmol, 1.00 equiv). The target compound (*rac*)-1²-(2,3-dibutylcycloprop-2-en-1-yl)-[2.2]paracyclophane hexachloroantimonate (**11c**) was isolated as a yellow solid in 95% yield (40.2 mg, 58.1 µmol).

Decomp. at 135–139 °C

^{1}H NMR (400 MHz, Dichloromethane-d_2 [5.32 ppm], ppm) δ = 7.33 (s, 1H), 7.10–7.08 (m, 1H), 6.91–6.89 (m, 1H), 6.73–6.67 (m, 2H), 6.44–6.43 (m, 1H), 6.13–6.11 (m, 1H), 3.56–3.17 (m, 11H), 3.04–3.00 (m, 1H), 2.06–2.03 (m, 4H), 1.60–1.57 (m, 4H), 1.09–1.07 (m, 6H).

^{13}C NMR (100 MHz, Dichloromethane-d_2 [53.8 ppm], ppm) δ = 171.2 (C_q, 2C), 165.4 (C_q), 149.7 (C_q), 145.3 (CH), 143.8 (C_q), 140.9 (CH), 140.7 (C_q), 138.5 (C_q), 137.3 (CH), 133.9 (CH), 133.3 (CH), 133.3 (CH), 132.9 (CH), 120.1 (C_q), 36.3 (CH$_2$), 35.5 (CH$_2$), 35.2 (CH$_2$), 34.9 (CH$_2$), 28.7 (CH$_2$, 2C), 28.5 (CH$_2$, 2C), 23.2 (CH$_2$, 2C), 13.8 (CH$_3$, 2C).

MS (ESI+, DCM) m/z (%): 357 (100) [M – $SbCl_6$]$^+$.

HRMS (ESI+, DCM) m/z (%) calcd for $C_{27}H_{33}^+$ [M – $SbCl_6$]$^+$: 357.2577, found 357.2568.

IR (ATR, ṽ) = 2961 (m), 2931 (m), 2897 (w), 2870 (w), 1837 (w), 1591 (w), 1550 (w), 1489 (w), 1445 (vs), 1422 (vs), 1374 (vs), 1303 (w), 1234 (w), 1204 (w), 1187 (m), 1159 (w), 1111 (w), 1095 (m), 1078 (m), 986 (w), 972 (w), 963 (w), 946 (w), 905 (m), 882 (w), 860 (m), 796 (m), 721 (s), 687 (w), 645 (w), 608 (m), 506 (vs), 490 (m), 479 (m), 467 (m), 459 (m), 448 (m), 438 (m), 408 (w), 399 (m), 387 (m) cm^{-1}.

UV/VIS (absorption, DCM, 22 °C), λ_{max} = 327, 265 nm.

Additional information on the experimental procedure is available via the repository Chemotion: https://dx.doi.org/10.14272/reaction/SA-FUHFF-UHFFFADPSC-NTBFMJQLMA-UHFFFADPSC-NUHFF-HUHFF-NUHFF-ZZZ

Additional information on the characterization of the target compound is available via the repository Chemotion: https://dx.doi.org/10.14272/NTBFMJQLMALANJ-UHFFFAOYSA-H.1

(*rac*)-1²-(2-Ethyl-3-methylcycloprop-2-en-1-yl)-[2.2]paracyclophane hexachloroantimonate (11d)

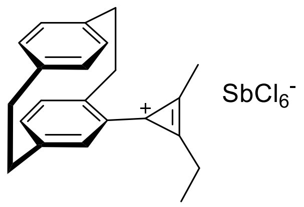

Following the general procedure **B** employing (*rac*)-1²-(2-ethyl-3-methylcycloprop-2-en-1-yl)-[2.2]paracyclophane (114 mg, 395 µmol, 1.00 equiv) (**3f**) and triphenylcarbenium hexachloroantimonate (228 mg, 395 µmol, 1.00 equiv). The target compound (*rac*)-1²-(2-ethyl-3-methylcycloprop-2-en-1-yl)-[2.2]paracyclophane hexachloroantimonate (**11d**) was isolated as a yellow solid in 94% yield (232 mg, 373 µmol).

Decomp. at 158–160 °C

^{1}H NMR (400 MHz, Dichloromethane-d_2 [5.32 ppm], ppm) δ = 7.34 (d, *J* = 1.9 Hz, 1H), 7.09 (dd, *J* = 7.9 Hz, *J* = 1.8 Hz, 1H), 6.89 (d, *J* = 8.0 Hz, 1H), 6.69 (qd, *J* = 7.8 Hz, *J* = 1.8 Hz, 2H), 6.44 (dd, *J* = 8.0 Hz, *J* = 1.8 Hz, 1H), 6.15 (dd, *J* = 8.0 Hz, *J* = 1.9 Hz, 1H), 3.60–3.27 (m, 8H), 3.23 (s, 3H), 3.21–3.12 (m, 2H), 1.71–1.67 (m, 3H).

^{13}C NMR (100 MHz, Dichloromethane-d_2 [53.8 ppm], ppm) δ = 172.3 (C_q), 167.9 (C_q), 165.4 (C_q), 149.6 (C_q), 145.2 (*C*H), 143.7 (C_q), 140.8 (*C*H), 140.7 (C_q), 138.5 (C_q), 137.3 (*C*H), 133.8 (*C*H), 133.3 (*C*H), 133.2 (*C*H), 132.9 (*C*H), 120.1 (C_q), 36.2 (CH_2), 35.4 (CH_2), 35.1 (CH_2), 34.8 (CH_2), 22.4 (CH_2), 14.3 (CH_3), 10.7 (CH_3).

MS (ESI+, DCM) m/z (%): 287 (100) $[M]^+$.

HRMS (ESI+, DCM) m/z (%) calcd for $C_{22}H_{23}^+$ $[M]^+$: 287.1794, found 287.1796.

IR (ATR, ṽ) = 2990 (w), 2941 (m), 2927 (m), 2895 (m), 2873 (w), 2856 (m), 1839 (w), 1588 (m), 1548 (m), 1487 (m), 1459 (vs), 1442 (vs), 1408 (vs), 1381 (vs), 1302 (vs), 1247 (m), 1204 (m), 1184 (m), 1160 (m), 1136 (w), 1106 (m), 1092 (m), 1082 (m), 1040 (m), 1000 (w), 983 (w), 963 (w), 950 (m), 924 (w), 907 (m), 882 (m), 867 (s), 820 (w), 799 (s), 768 (w), 724 (vs), 700 (w), 676 (m), 645 (m), 609 (s), 511 (s) cm^{-1}.

UV/VIS (absorption, DCM, 22 °C), λ_{max} = 325, 265 nm.

Additional information on the experimental procedure is available via the repository Chemotion: https://dx.doi.org/10.14272/reaction/SA-FUHFF-UHFFFADPSC-JHPCEAUKLJ-UHFFFADPSC-NUHFF-HUHFF-NUHFF-ZZZ

Additional information on the characterization of the target compound is available via the repository Chemotion: https://dx.doi.org/10.14272/JHPCEAUKLJCAFF-UHFFFAOYSA-H.1

3.1. Reaction Optimization

We first evaluated different rhodium- and silver-based catalysts. Several Ag(I) salts gave slightly higher NMR yields compared to $Rh_2(esp)_2$, but the reactions produced complex mixtures that hindered product isolation. An exception was Ag(I) tetrafluoroborate, which afforded a significantly higher NMR yield (50%), yet the product could not be purified to analytical quality under standard chromatographic conditions. Among the screened rhodium catalysts, Rh_2(*S*-BTPCP)$_4$ gave the highest NMR yield (19%), whereas rhodium(II) acetate and rhodium(II) tetracaprolactamate afforded lower yields (4% and 5% respectively). No product formation was

observed with Fe(TPP)Cl or [Cp*Rh(III)Cl_2]$_2$. $Rh_2(esp)_2$ was selected for further optimization due to its commercial availability.

Increasing the alkyne loading from 1.00 to 2.20 equivalents improved the isolated yield to 20%, and addition of NaBARF as a weakly coordinating anion additive further enhanced the reaction performance, providing 54% isolated yield. Examination of different solvents showed that DCE gave comparable results to DCM, while THF did not provide any product. $PhCF_3$ afforded higher NMR yields (63%), but purification was complicated by increased byproduct formation. Variation of the reaction temperature resulted in lower yields.

These results define the optimized conditions as $Rh_2(esp)_2$ (5 mol%), 2.20 equivalents of the alkyne, and NaBARF as additive in DCM at 40 °C for 16 h (Table S1).

Table S1: Reaction optimization.

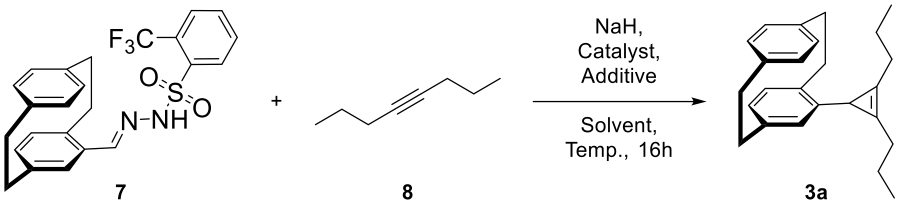

Entry	Deviation from standard conditions[a]	NMR yield[b]
1	$Rh_2(esp)_2$	8(6)
2	Ag(I) hexafluoroantimonate	trace
3	Ag(I) trifluoroacetate	12
4	Ag(I) carbonate	10
5	Ag(I) triflate	11
6	Ag(I) tetrafluoroborate	50
7	$AgTp(CF_3)_2$	12
8	Rh(II) acetate	4
9	Rh(II) tetracaprolactamat	5
10	Rh_2(*S*-BTPCP)$_4$	19
11	[Cp*Rh(III)Cl_2]$_2$	n.d.
12	Fe(TPP)Cl	n.d.
13	$Rh_2(esp)_2$, 2.20 equiv. 3-octyne	25(20)
14	5 mol% NaBARF additive	58(54)
15	DCE	56
16	THF	n.d.
17	$PhCF_3$	63
18	30 °C	33
19	60 °C (pressure tube)	35

[a]Standard conditions: **7** (1.00 equiv.), 3-octyne (**8**) (1.00) equiv, catalyst (5 mol% for Rh-catalysts or 10 mol% for Ag-catalysts), DCM, 50 °C, 16 h. Isolated yields in parantheses. [b]NMR yields were determined using 1,3,5-trimethoxybenzene as internal standard.

3.2. Limitations: Functionalized Alkynes

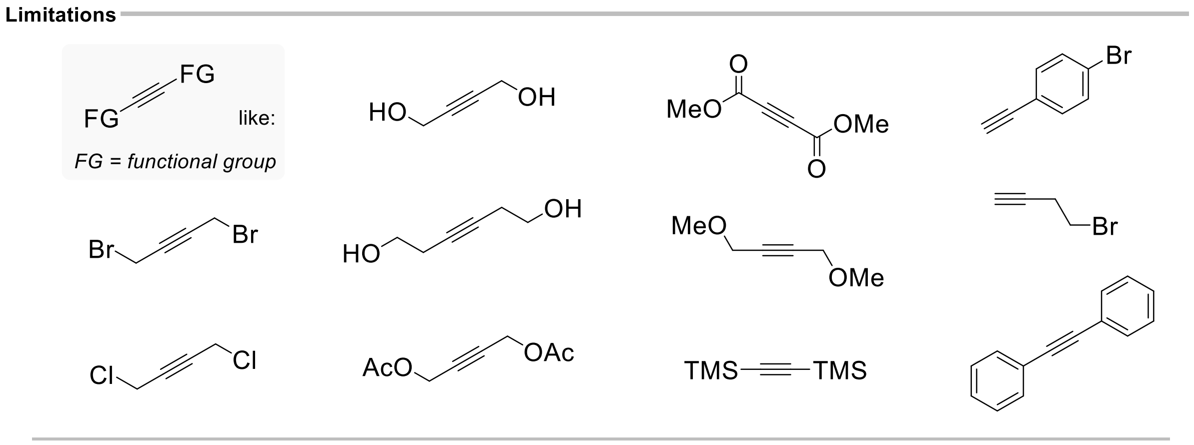

Scheme S1: Limitations of functionalized alkynes.

3.3. High-Temperature NMR Experiment

A high-temperature NMR experiment was performed on cyclopropene **3a** using DMSO-d_6 as solvent. The diastereotopic differentiation of the alkyl group remains observable even at 120 °C. This rules out hindered rotation as the origin of the diastereotopicity, since rotational averaging would be expected at elevated temperature. The finding is consistent with the DFT calculations.

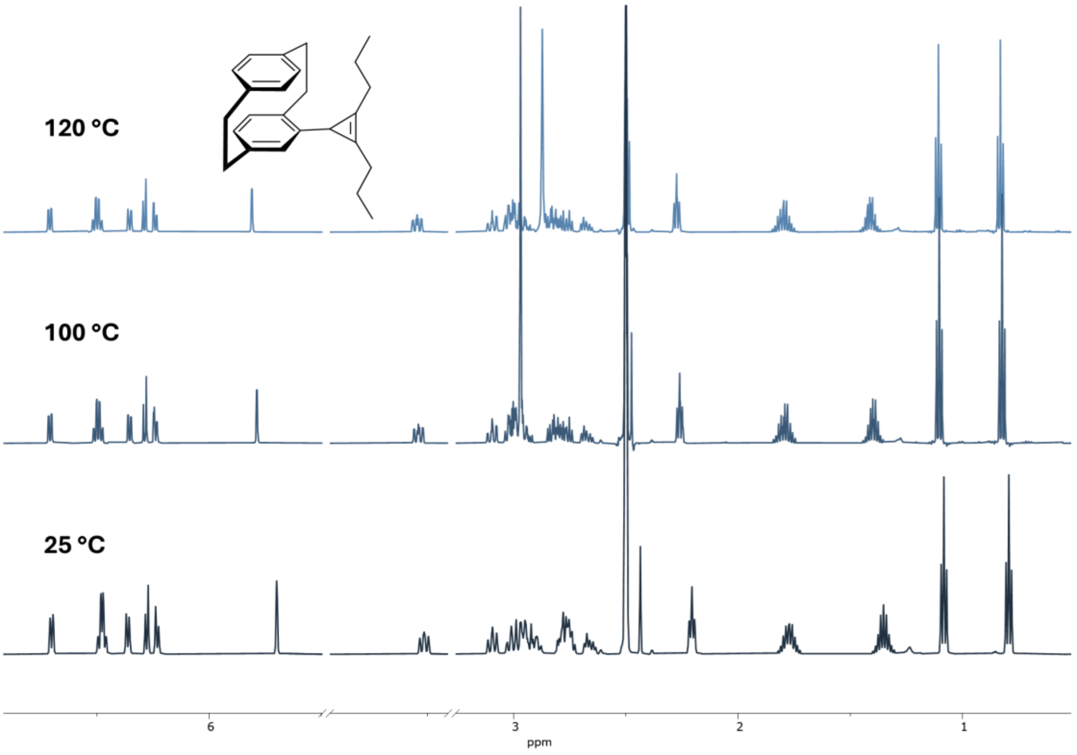

Figure S1: High-temperature NMR experiment of compound **3a**. Parts of the spectrum have been omitted for clarity.

4. X-ray Diffraction Data

Single crystal X-ray diffraction data of compound **11d** were collected on a STOE STADI VARI diffractometer with a DECTRIS EIGER 4M detector with monochromated Ga Kα (1.34143 Å) radiation generated by an EXCILLIUM MetalJet D2+ at low temperature. Using Olex2[6], the structures were solved with the ShelXT[7] structure solution program using Intrinsic Phasing and refined with the ShelXL[8] refinement package using Least Squares minimization. Refinement was performed with anisotropic temperature factors for all non-hydrogen atoms; hydrogen atoms were calculated on idealized positions. Crystallographic data and refinement details are summarized in Table S2.

The crystallographic data for compound **11d** reported in this paper has been deposited with the Cambridge Crystallographic Data Centre as supplementary information no. CCDC-2524363. Copies of the data can be obtained free of charge from https://www.ccdc.cam.ac.uk/structures/.

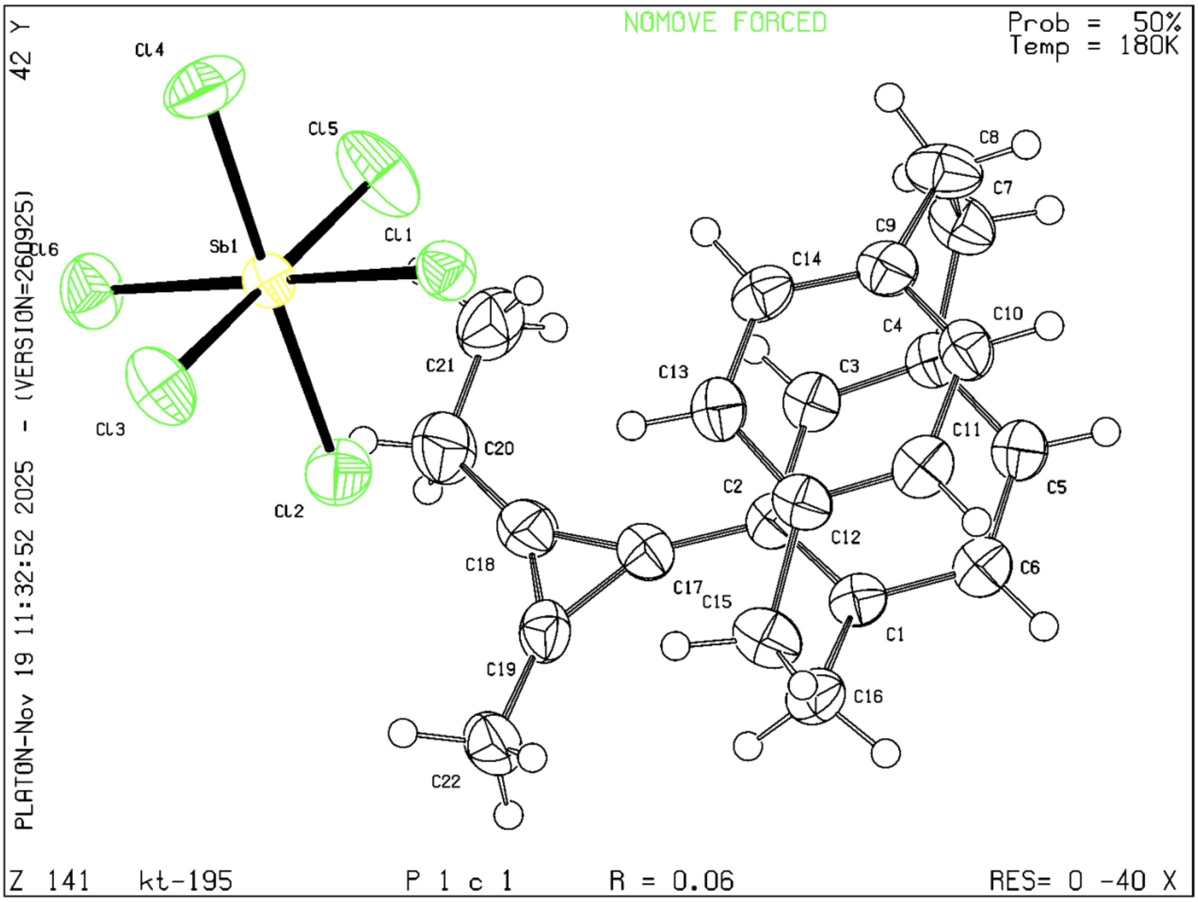

Figure S2: Ellipsoid plot for compound **11d**.

Table S2: Crystal data and structure refinement for compound **11d**.

CCDC Number	2524363
Empirical formula	$C_{22}H_{23}Cl_6Sb$
Formula weight	621.85
Temperature/K	180
Crystal system	monoclinic
Space group	*Pc*
a/Å	8.2374(2)
b/Å	10.0598(3)
c/Å	14.6407(4)
α/°	90
β/°	95.580(2)
γ/°	90
Volume/Å^3	1207.48(6)
Z	2
ρ_{calc}g/cm^3	1.710
μ/mm^{-1}	10.445
F(000)	616.0
Crystal size/mm^3	0.28 × 0.14 × 0.02
Radiation	Ga Kα (λ = 1.34143)
2Θ range for data collection/°	9.386 to 128.622
Index ranges	-11 ≤ h ≤ 10, -13 ≤ k ≤ 11, -10 ≤ l ≤ 18
Reflections collected	10939
Independent reflections	4136 [R_{int} = 0.0245, R_{sigma} = 0.0198]
Independent reflections with I≥2σ (I)	4055
Data/restraints/parameters	4136/2/265
Goodness-of-fit on F^2	1.106
Final R indexes [I≥2σ (I)]	R_1 = 0.0588, wR_2 = 0.1612
Final R indexes [all data]	R_1 = 0.0591, wR_2 = 0.1617

5. Computational methods

All calculations were performed with the ORCA software package.[9] A global conformational search was carried out using the GOAT algorithm in combination with the GFN2-xTB method.[10,11] Subsequent NEB-TS searches were performed to investigate rotation about the pCp–C(cyclopropenyl) *σ*-bond for the relevant conformers of compound **3b** and **11a**.[12] All DFT calculations employed the PBE0 functional with the def2-TZVP basis set and D4 dispersion corrections.[13] The RIJCOSX approximation in combination with the def2/J auxiliary basis set was used. Solvent effects were included using the CPCM model with DCM as solvent.[14] Transition states were verified by the presence of a single imaginary frequency corresponding to the rotational mode, while minima were confirmed by the absence of imaginary frequencies.

Compound **3b**:

The global conformational search identified five conformers. Three of these differed primarily by rotation of the cyclopropene unit and were therefore selected for subsequent NEB-TS analysis of rotation about the pCp–C(cyclopropenyl) σ-bond. Upon reoptimization at the DFT level, one of these conformers relaxed to conformer 1, indicating that it does not correspond to a true local minimum at this level of theory. Accordingly, this conformer, which was identified in the initial GFN2-xTB-based global optimization, is not stable upon DFT reoptimization.

Ultimately, two distinct conformers were located at the DFT level, connected by two inequivalent transition states corresponding to clockwise and counterclockwise rotation. The calculated rotational barriers are 5.97 kcal mol^{-1} and 4.50 kcal mol^{-1}.

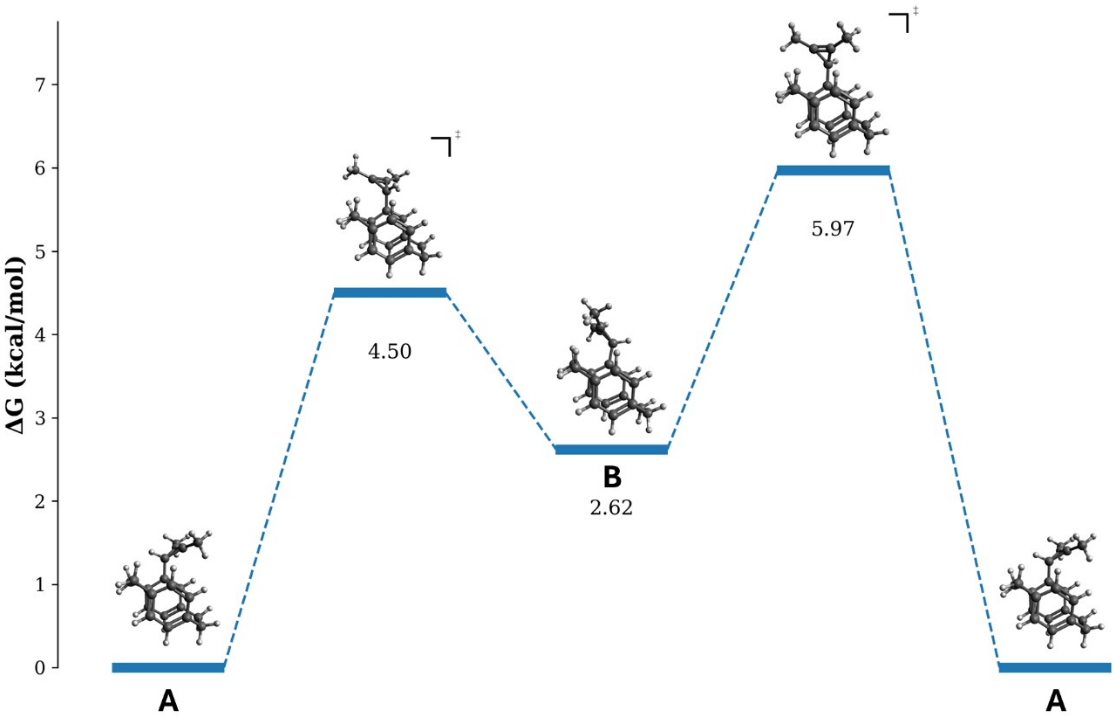

Figure S3: Rotational free-energy profile of compound **3b** calculated by DFT.

Compound **11a**:

The global conformational search identified three conformers, which mainly differ in the orientation of the methyl group. The lowest-energy conformer was subsequently used for NEB-TS calculations of rotation about the pCp–C(cyclopropenyl) σ-bond. Clockwise and counterclockwise rotations are energetically equivalent and connect the same conformer. The calculated rotational barrier is 10.30 kcal mol^{-1}.

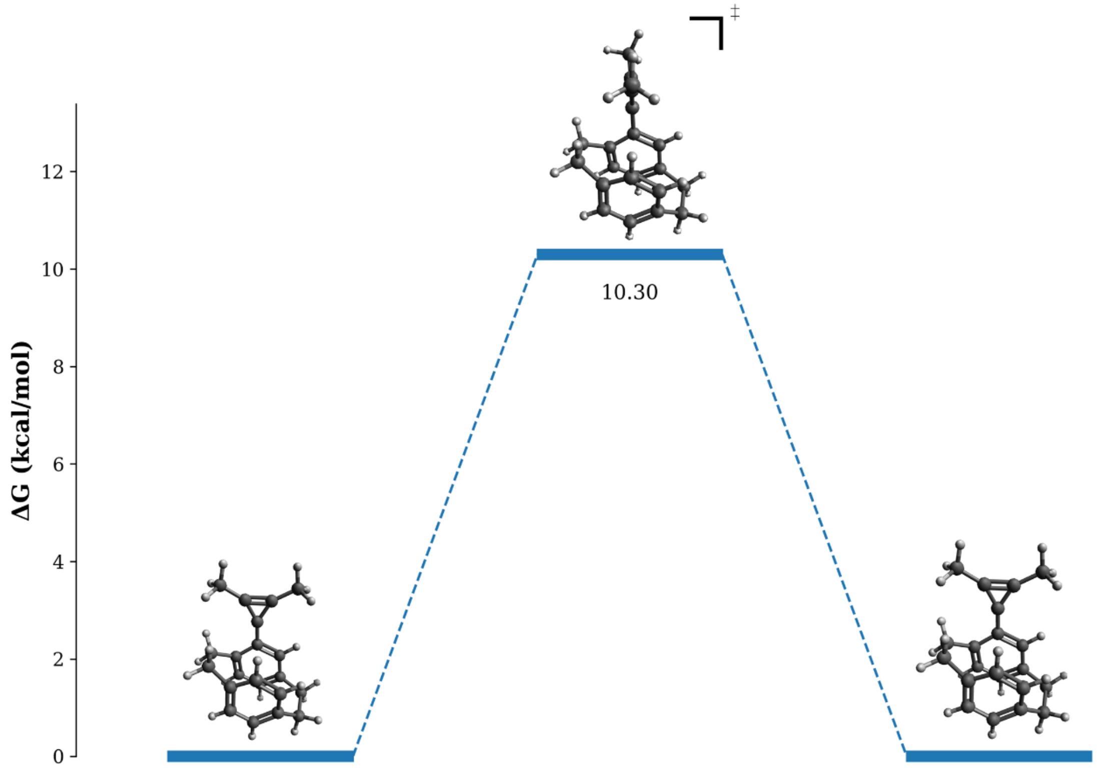

Figure S4: Rotational free-energy profile of compound **11a** calculated by DFT.

6. Spectra

6.1. NMR Spectra

(*rac*)-4-Formyl[2.2]paracyclophane (5)

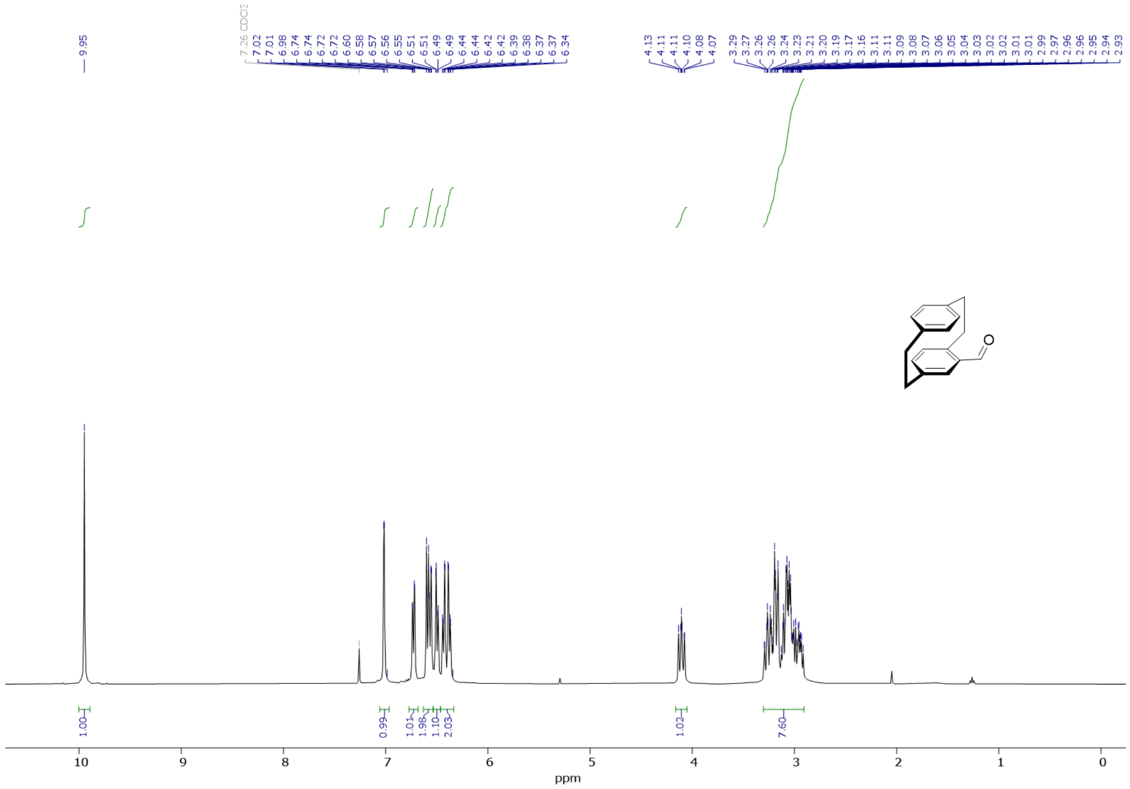

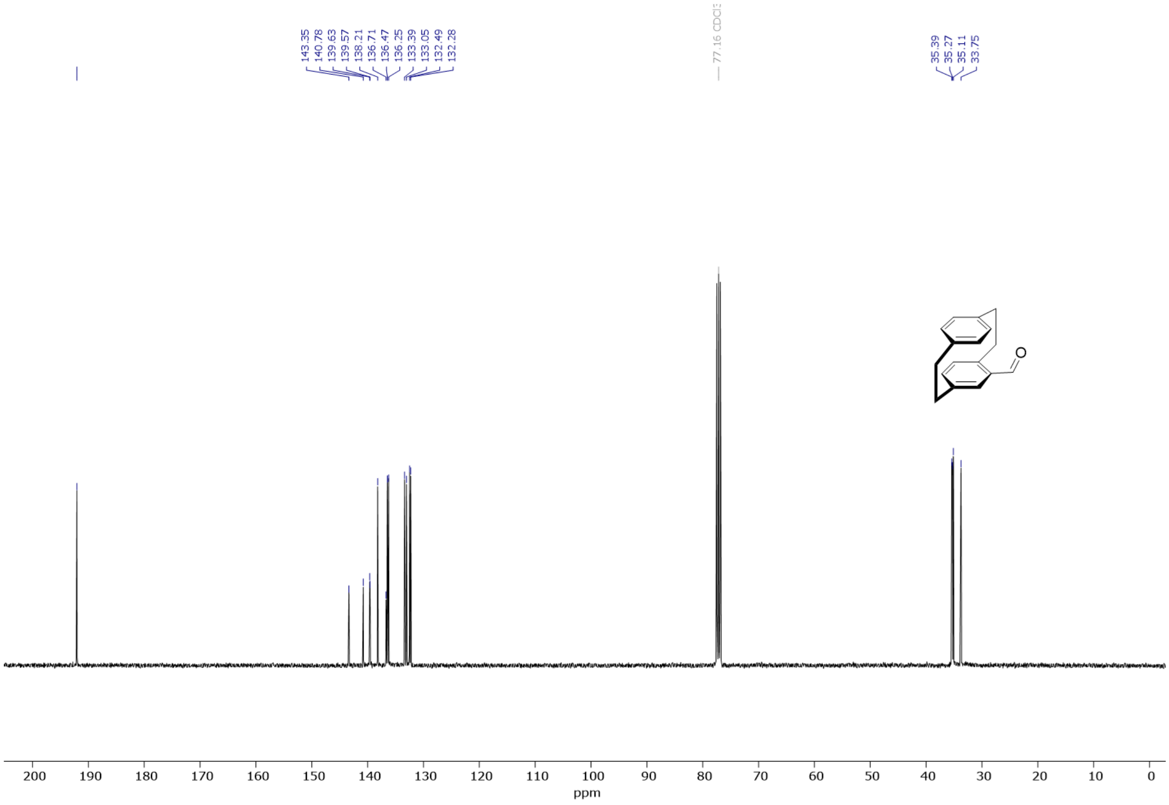

2-(Trifluoromethyl)benzenesulfonohydrazide (6)

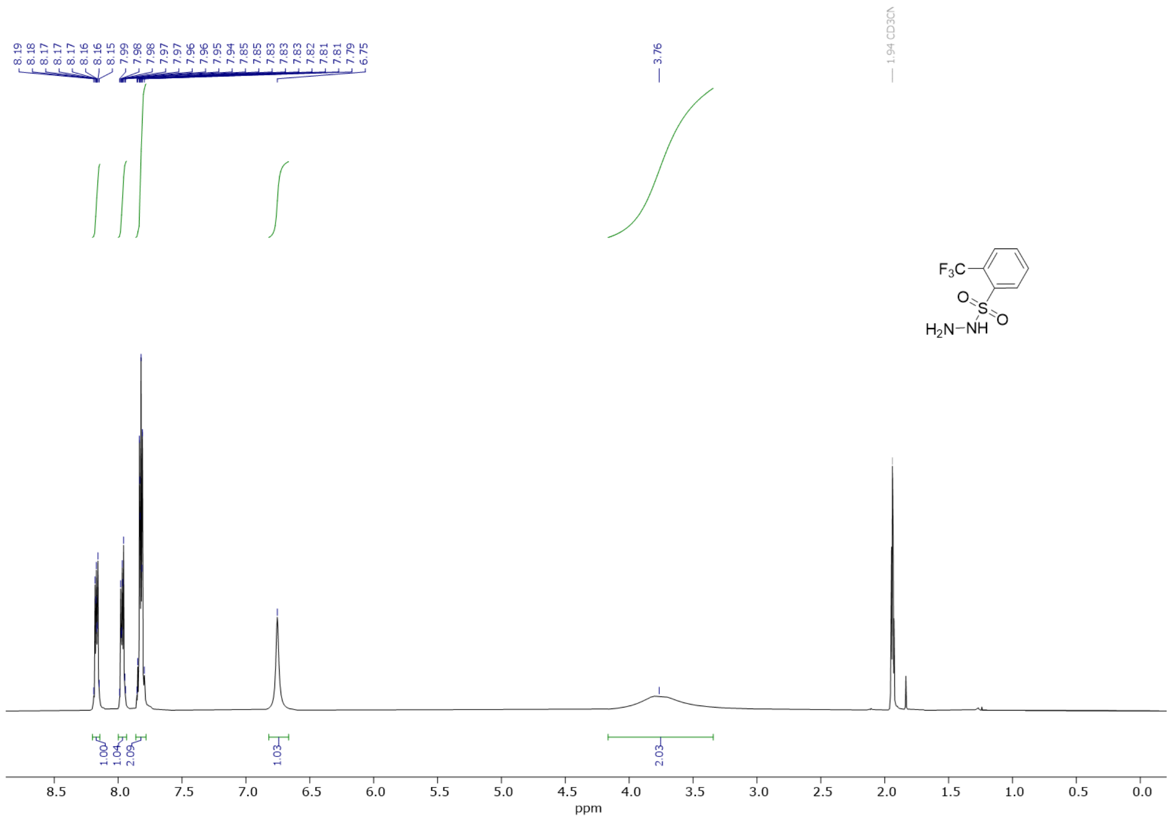

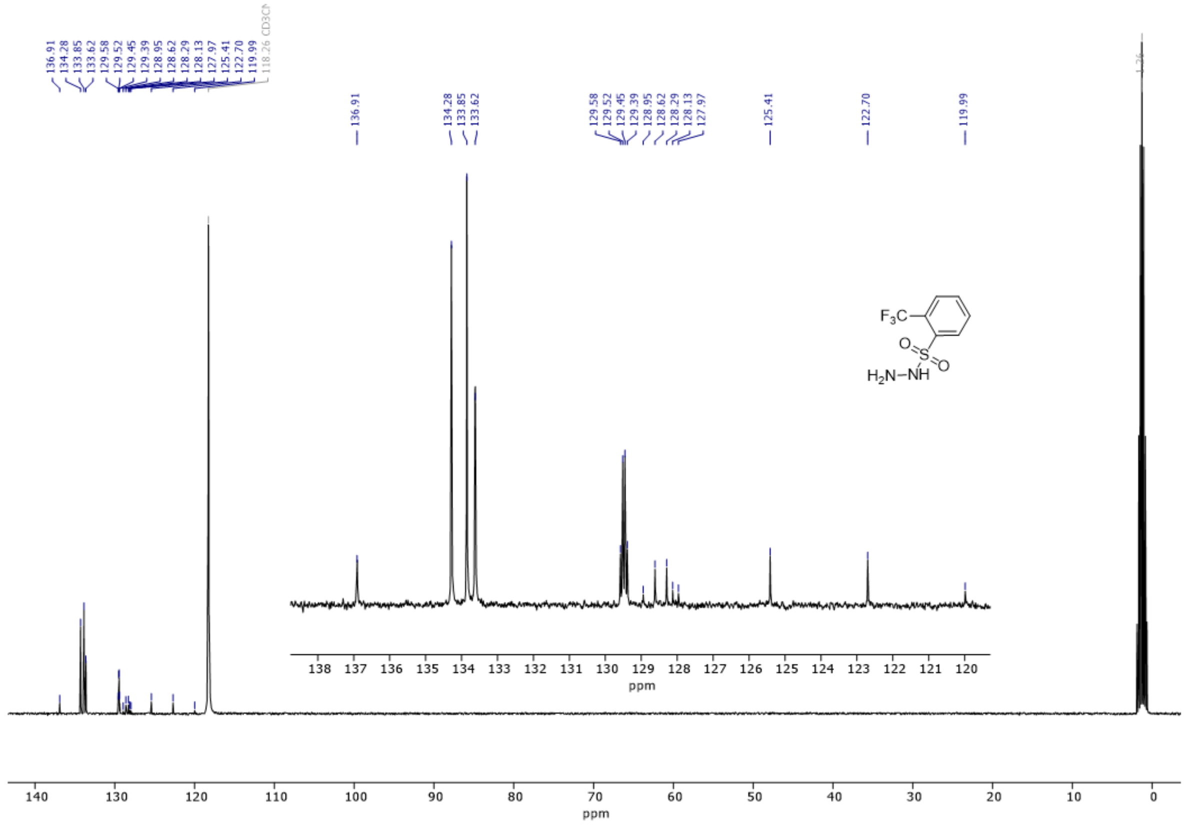

(*rac*)-*N*'-([2.2]Paracyclophanyl-1^2-ylmethylene)-2-(trifluoromethyl)benzohydrazide (7)

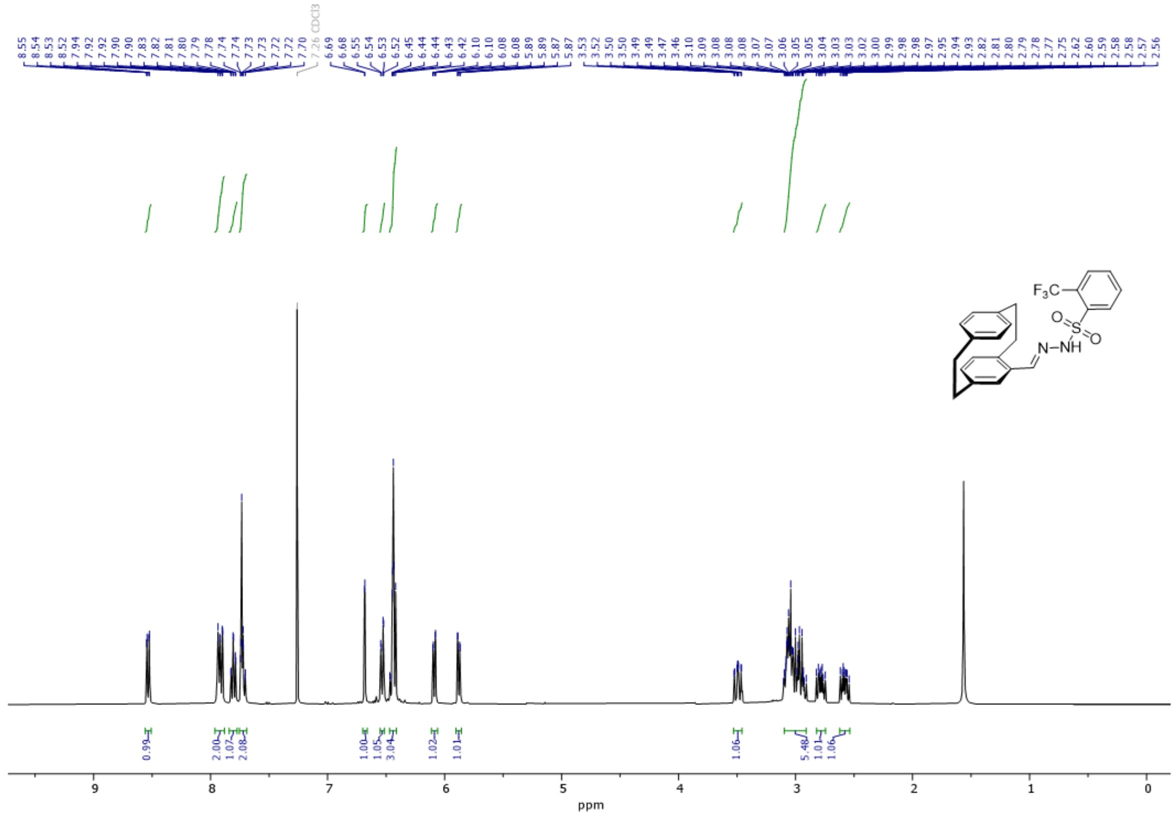

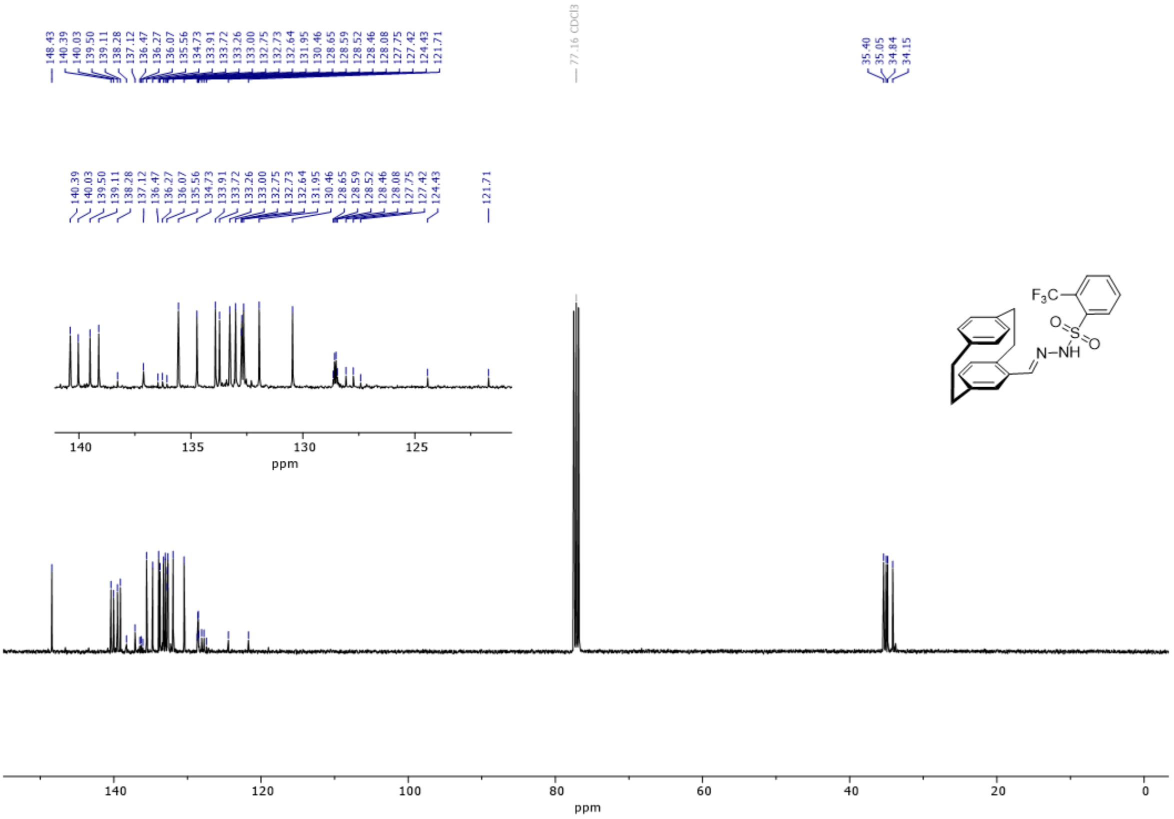

(*rac*)-1²-(2,3-Dimethylcycloprop-2-en-1-yl)-[2.2]paracyclophane (3b)

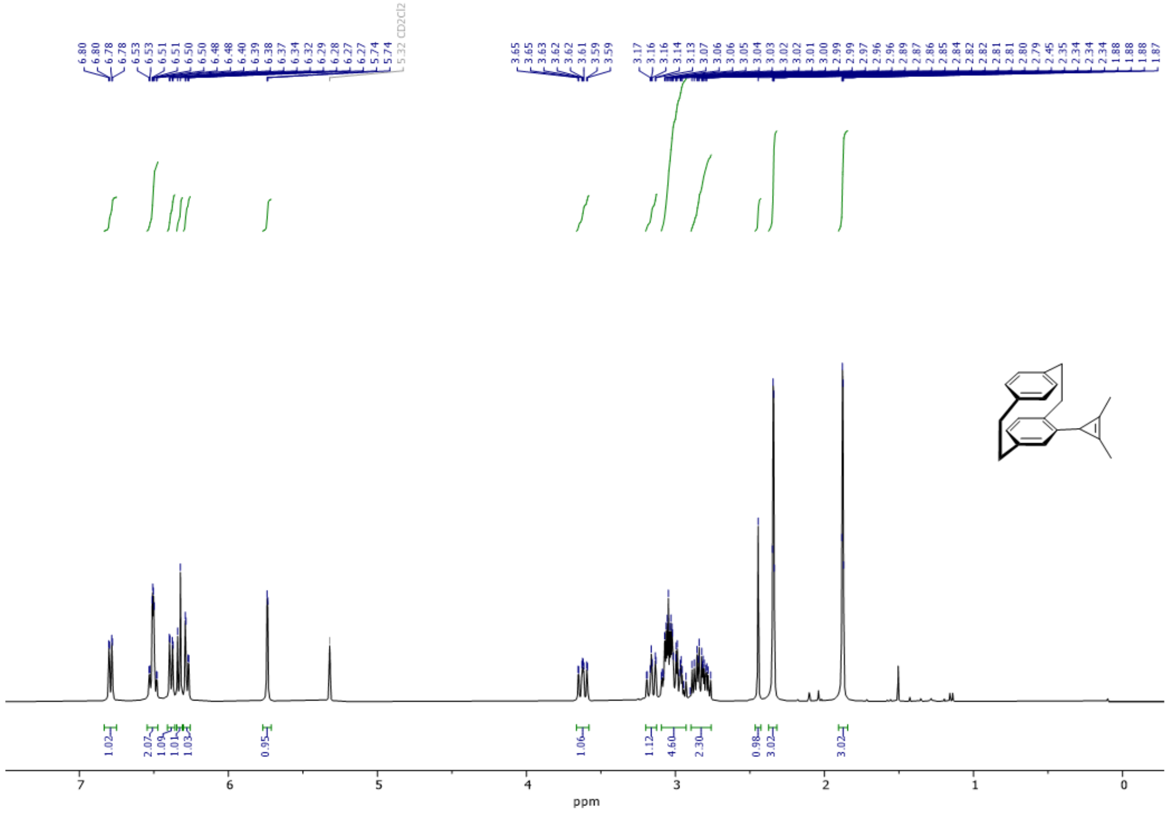

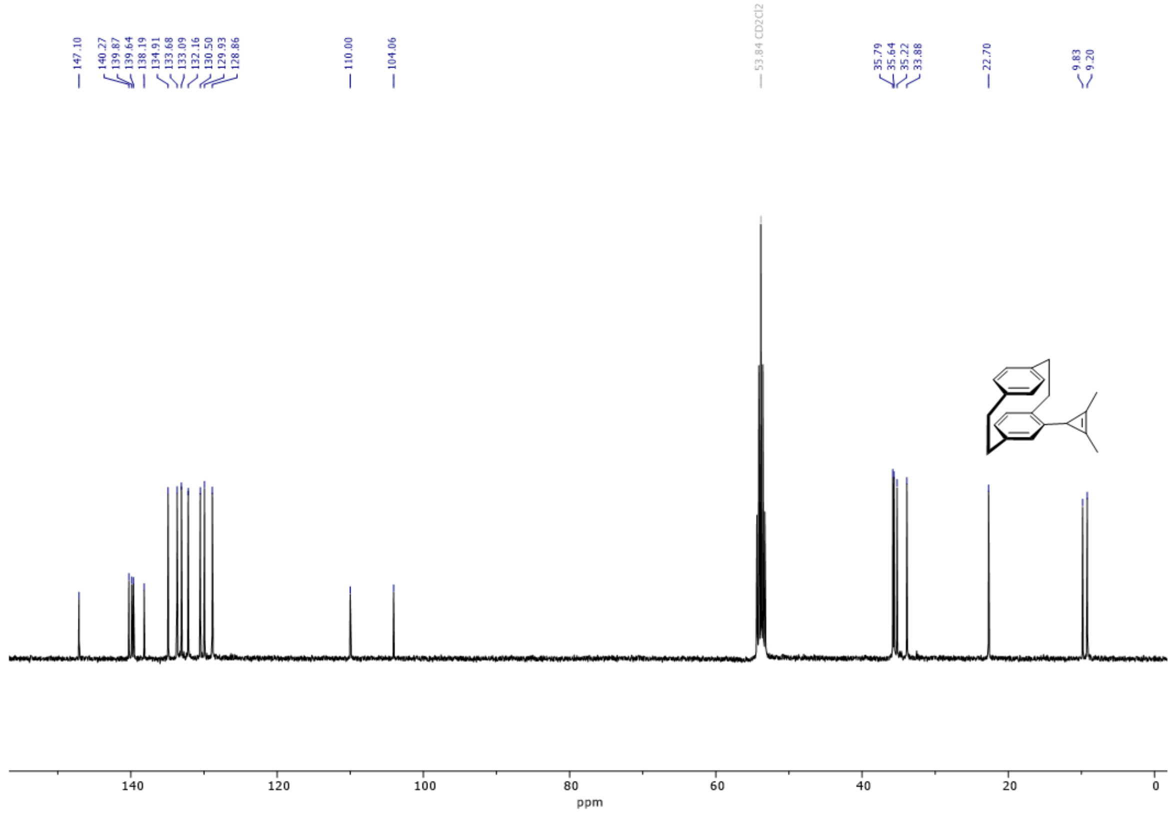

(*rac*)-1^2-(2,3-Diethylcycloprop-2-en-1-yl)-[2.2]paracyclophane (3c)

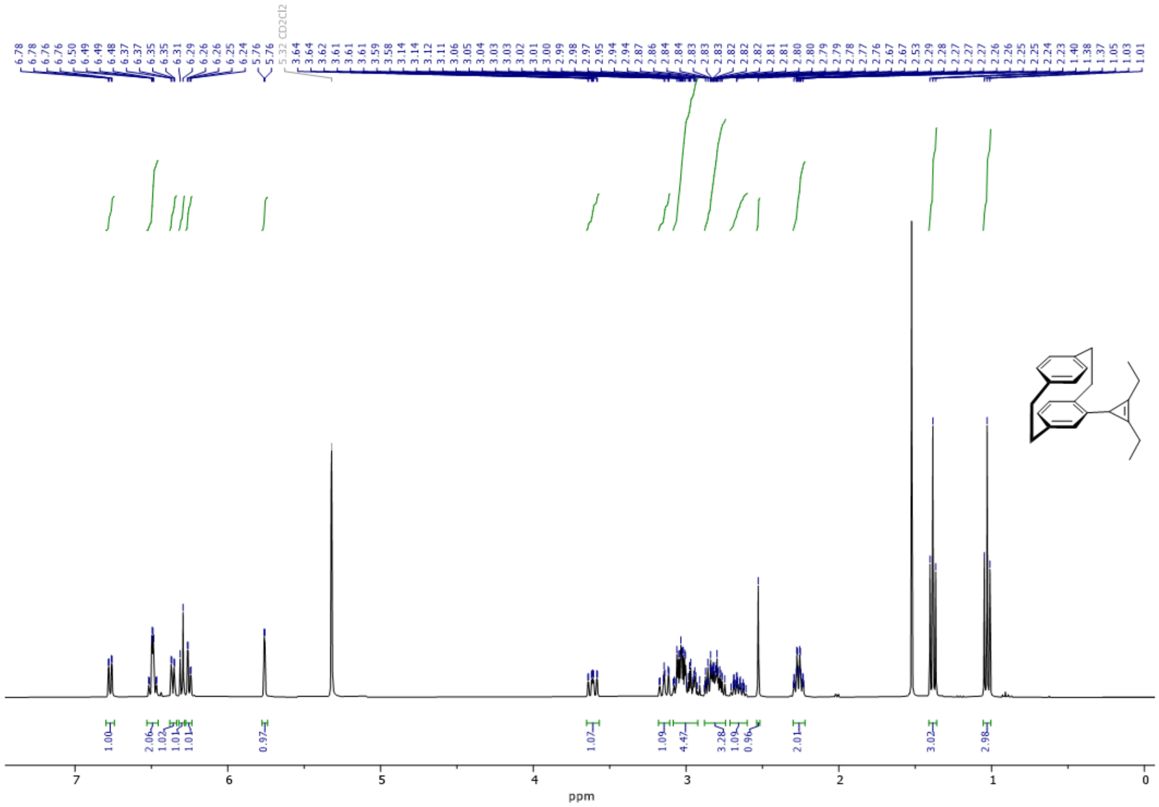

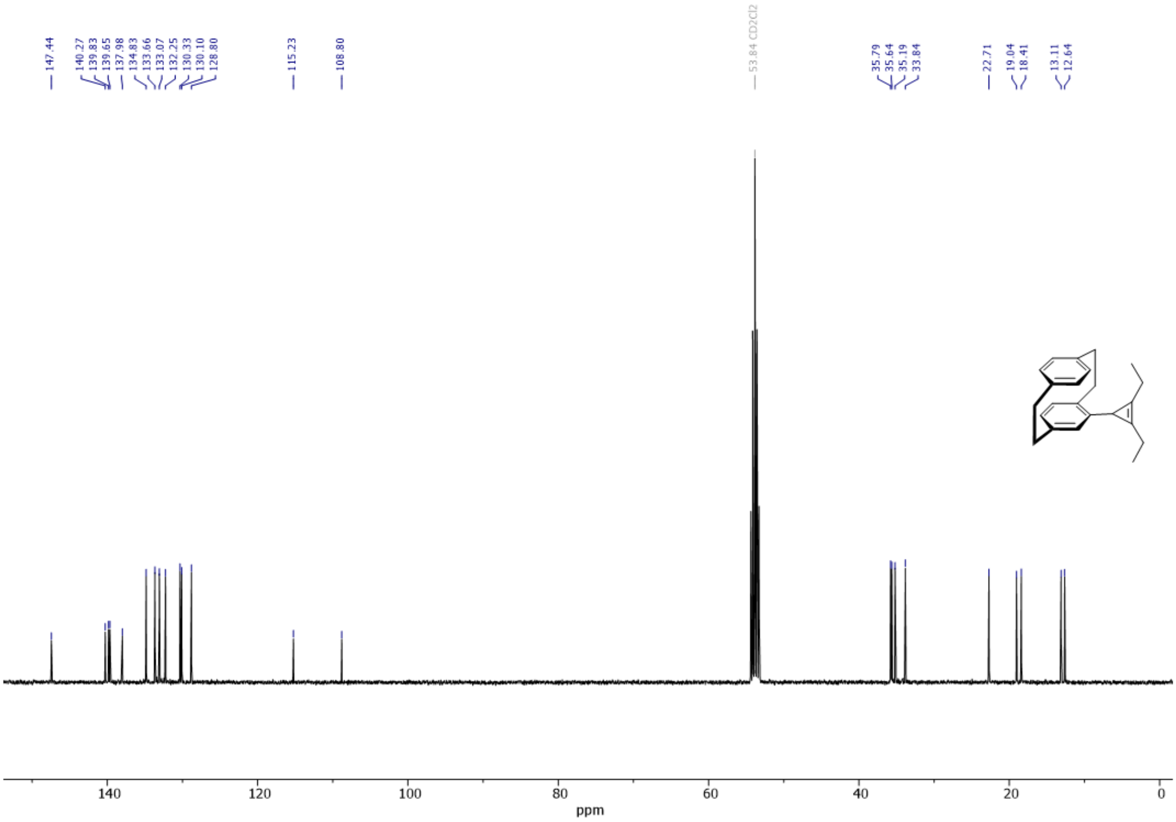

(*rac*)-1²-(2,3-Dipropylcycloprop-2-en-1-yl)-[2.2]paracyclophane (3a)

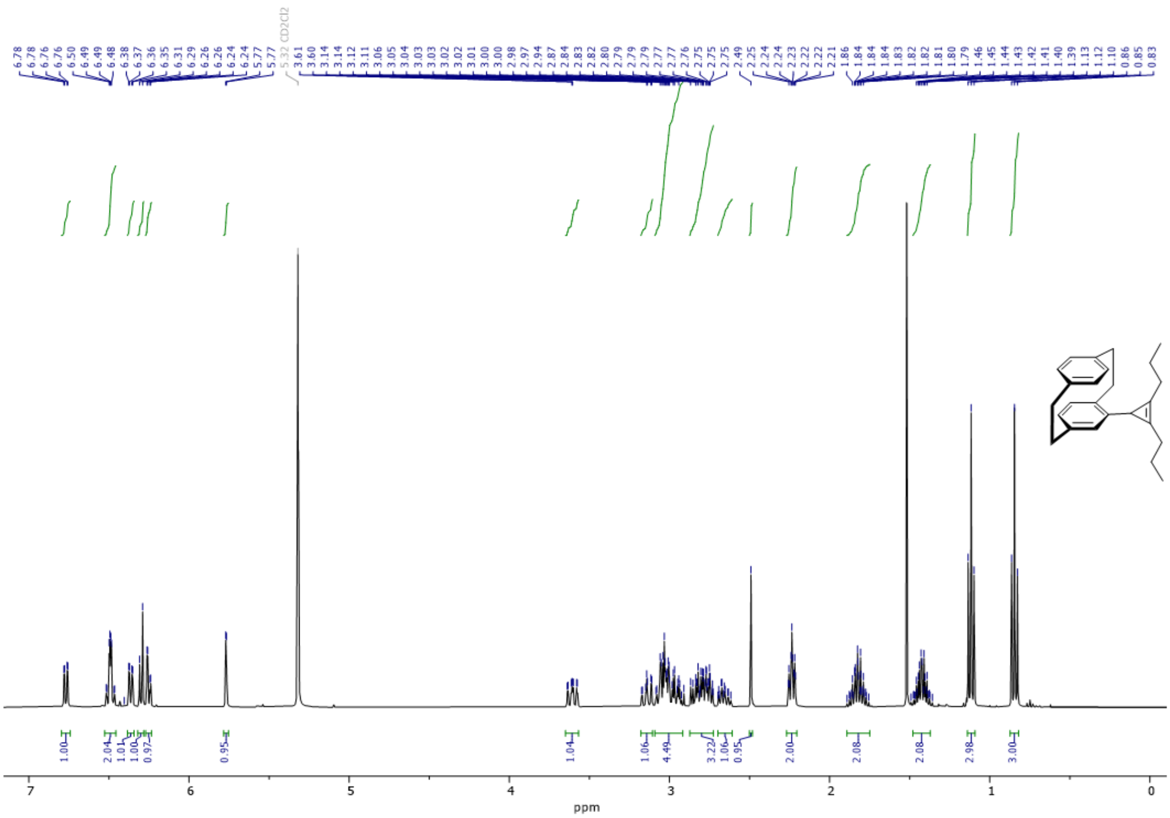

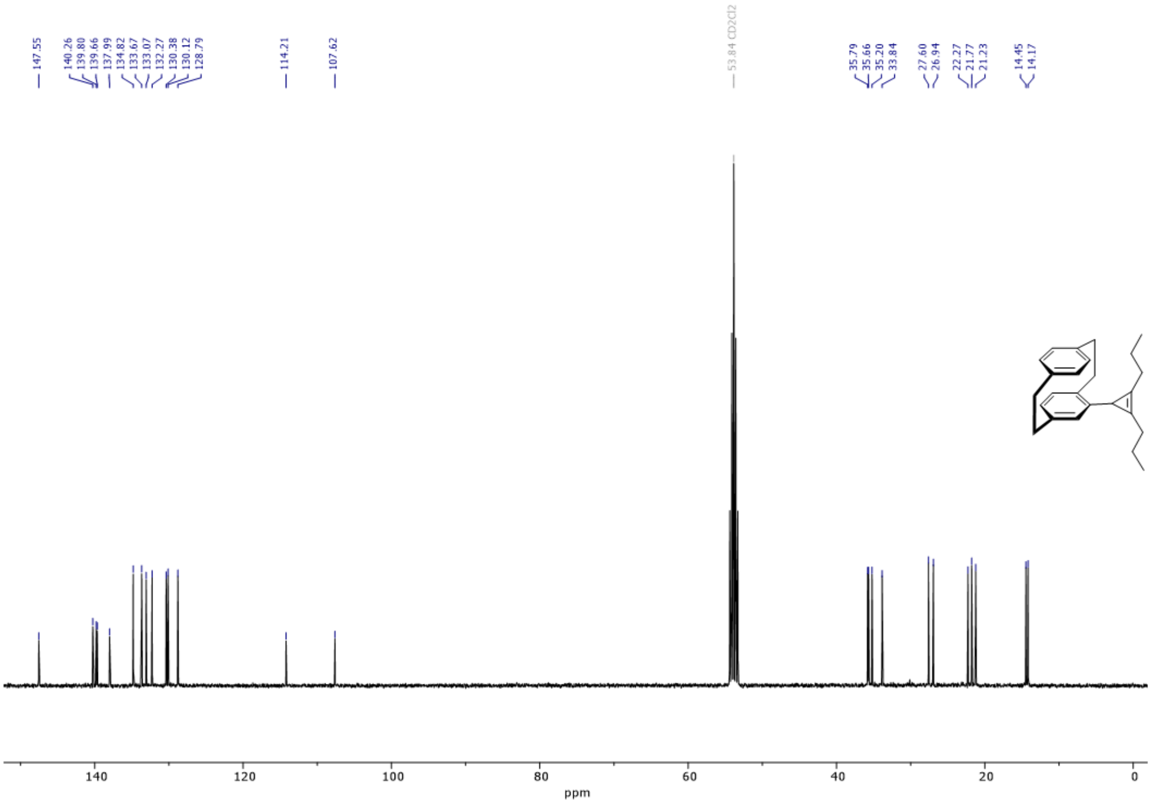

(*rac*)-1²-(2,3-Dibutylcycloprop-2-en-1-yl)-[2.2]paracyclophane (3d)

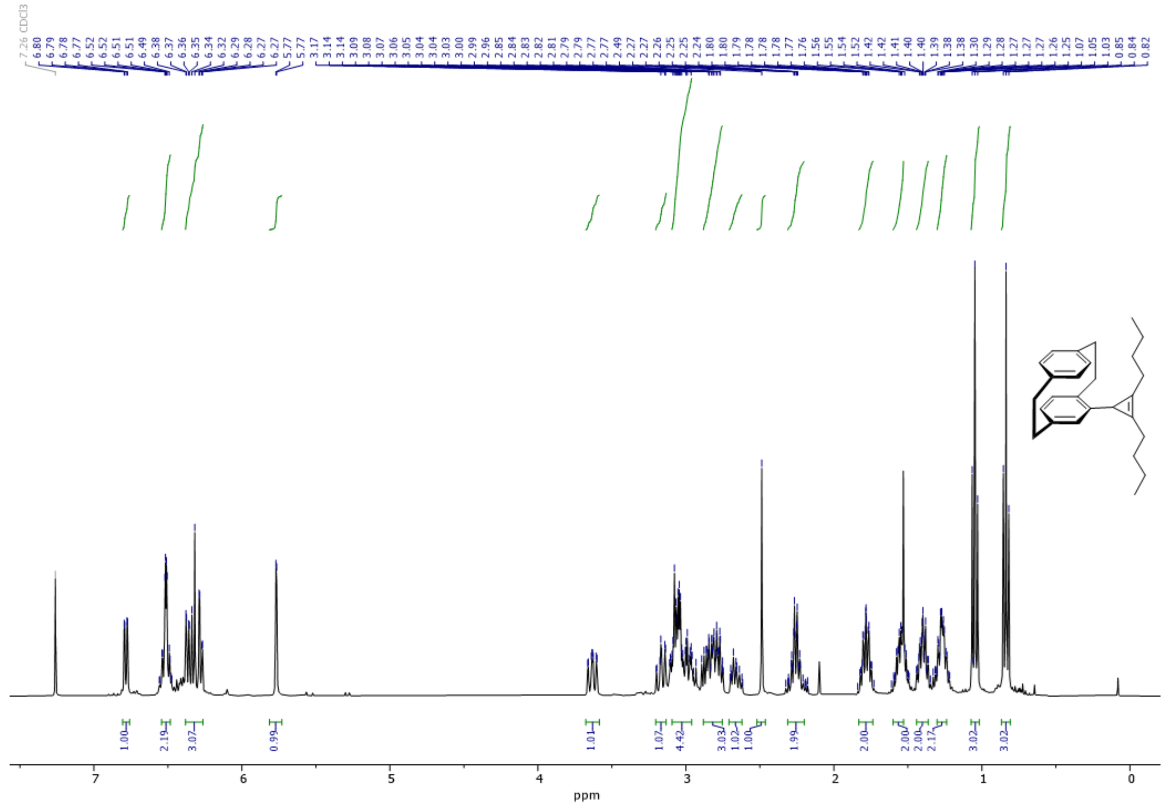

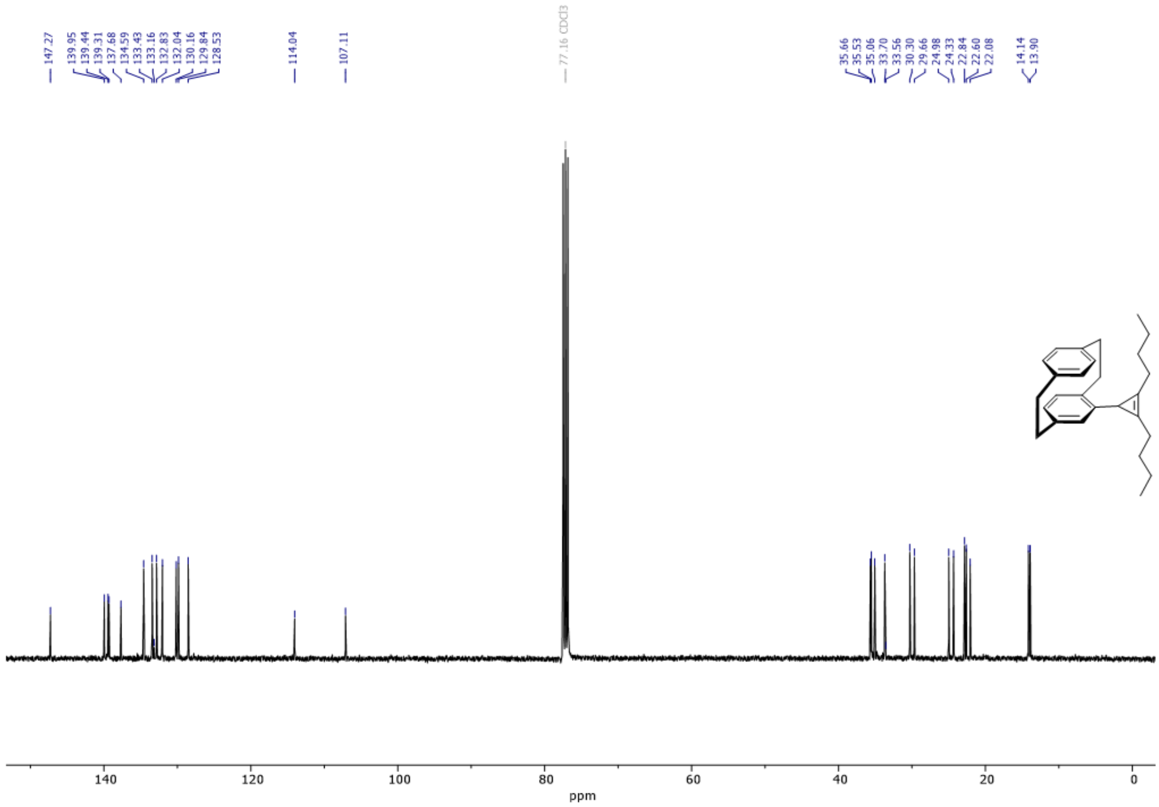

(*rac*)-1²-(2-(*tert*-Butyl)cycloprop-2-en-1-yl)-[2.2]paracyclophane (3e)

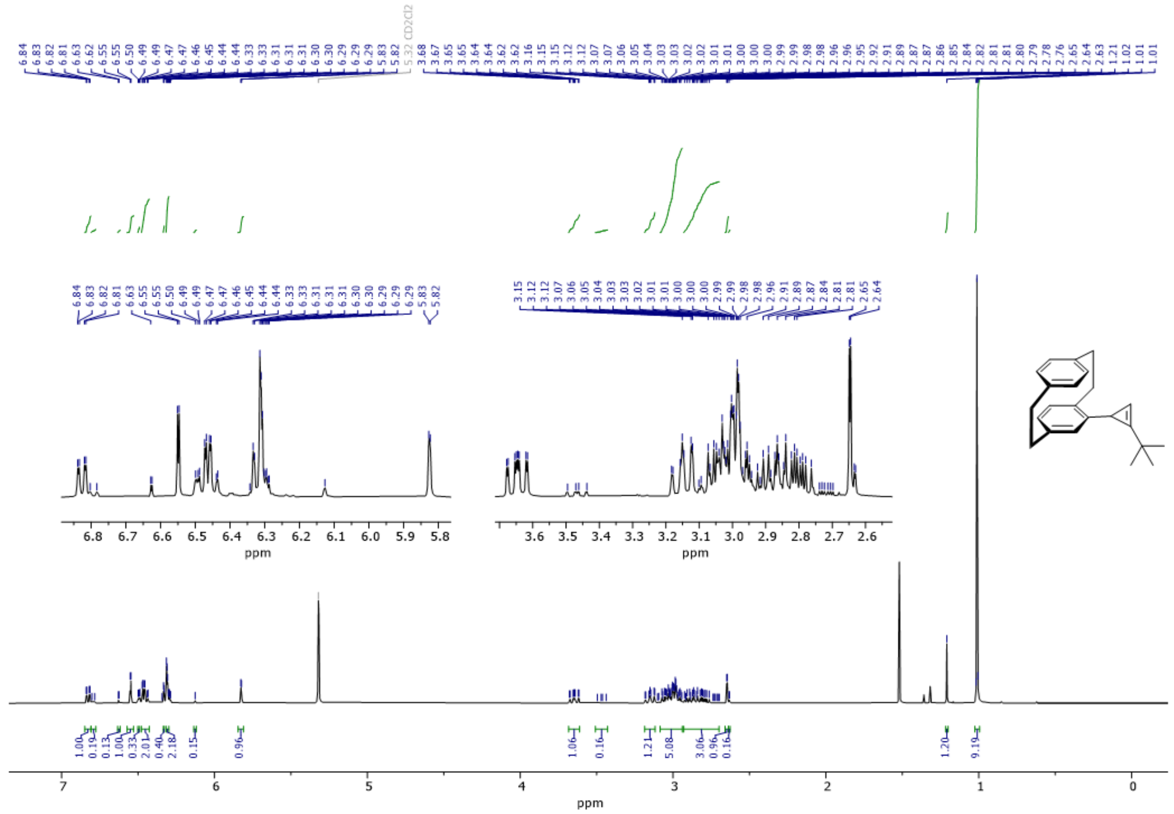

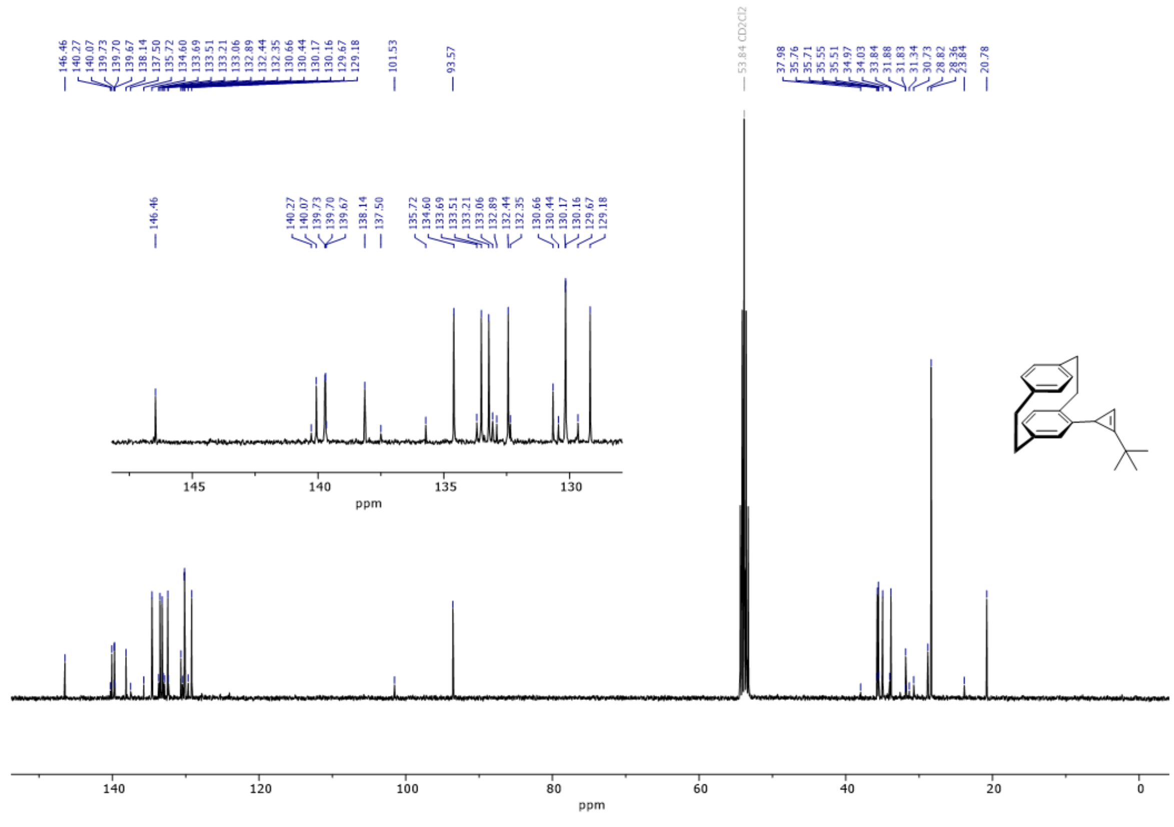

(*rac*)-1[2]-(2-Ethyl-3-methylcycloprop-2-en-1-yl)-[2.2]paracyclophane (3f)

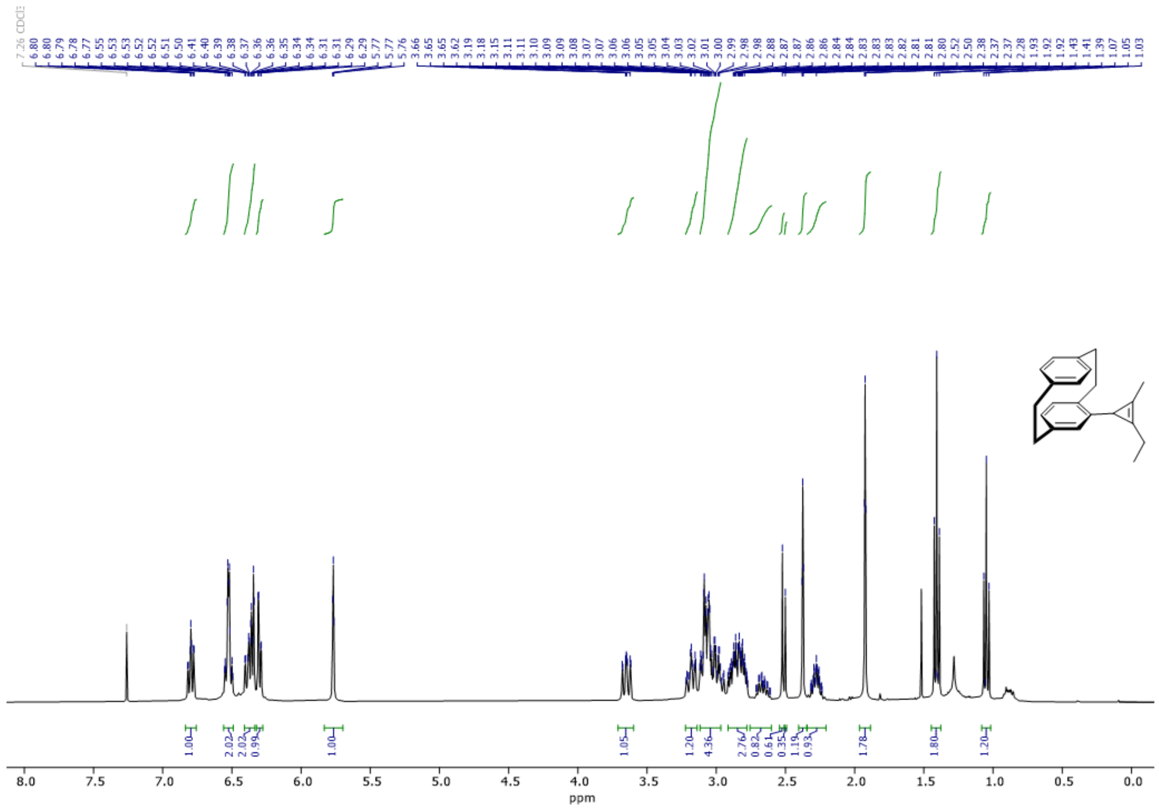

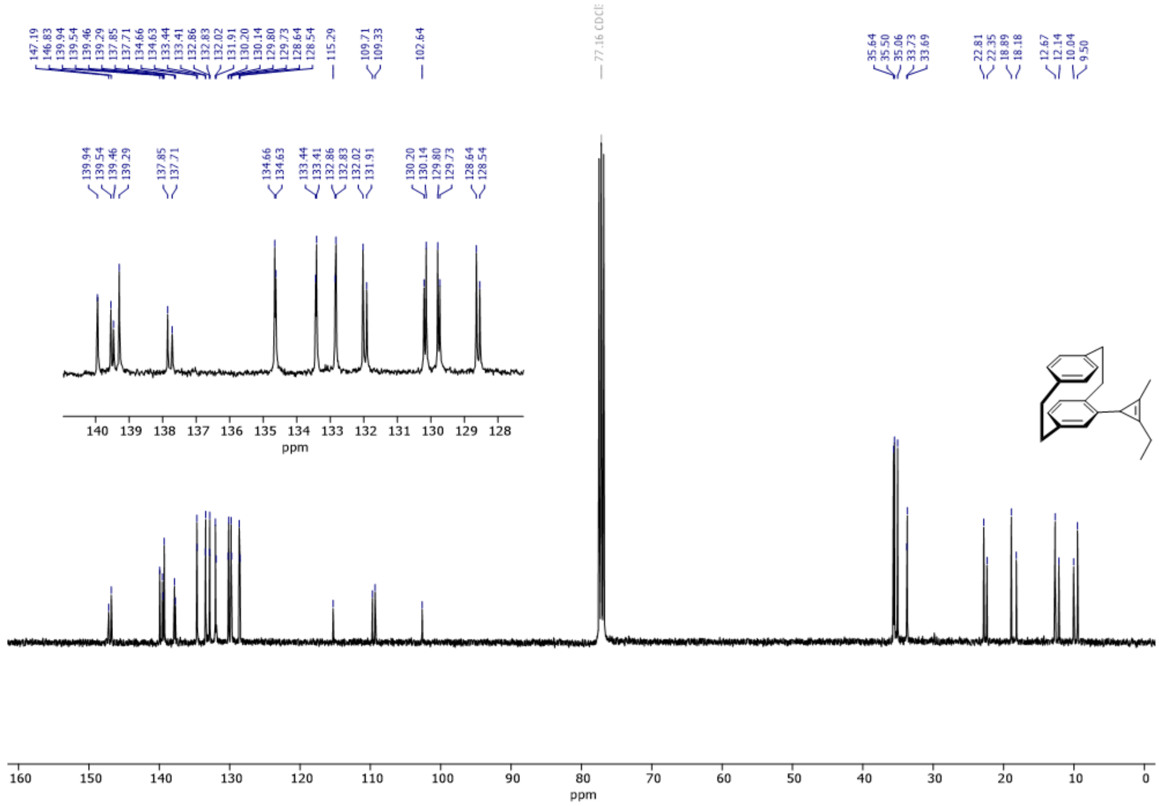

(*rac*)-1²-(2,3-Dimethylcycloprop-2-en-1-yl)-[2.2]paracyclophane hexachloroantimonate (11a)

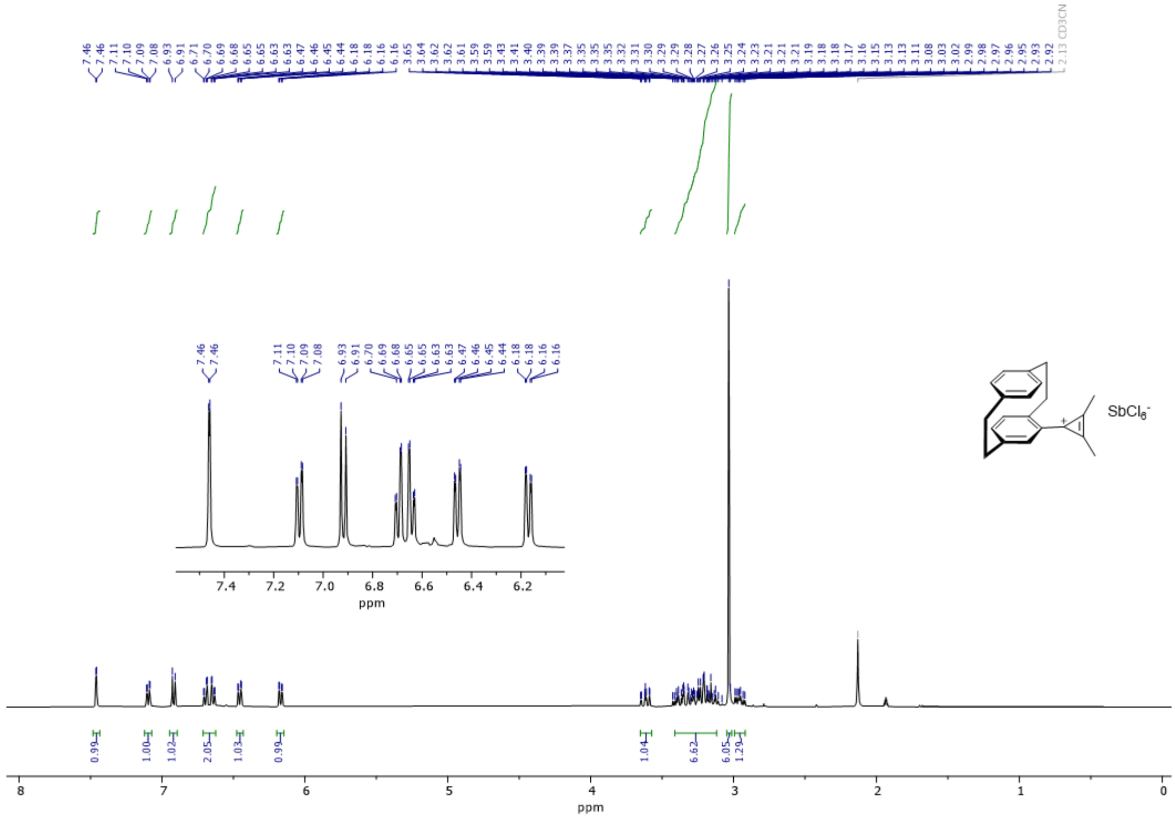

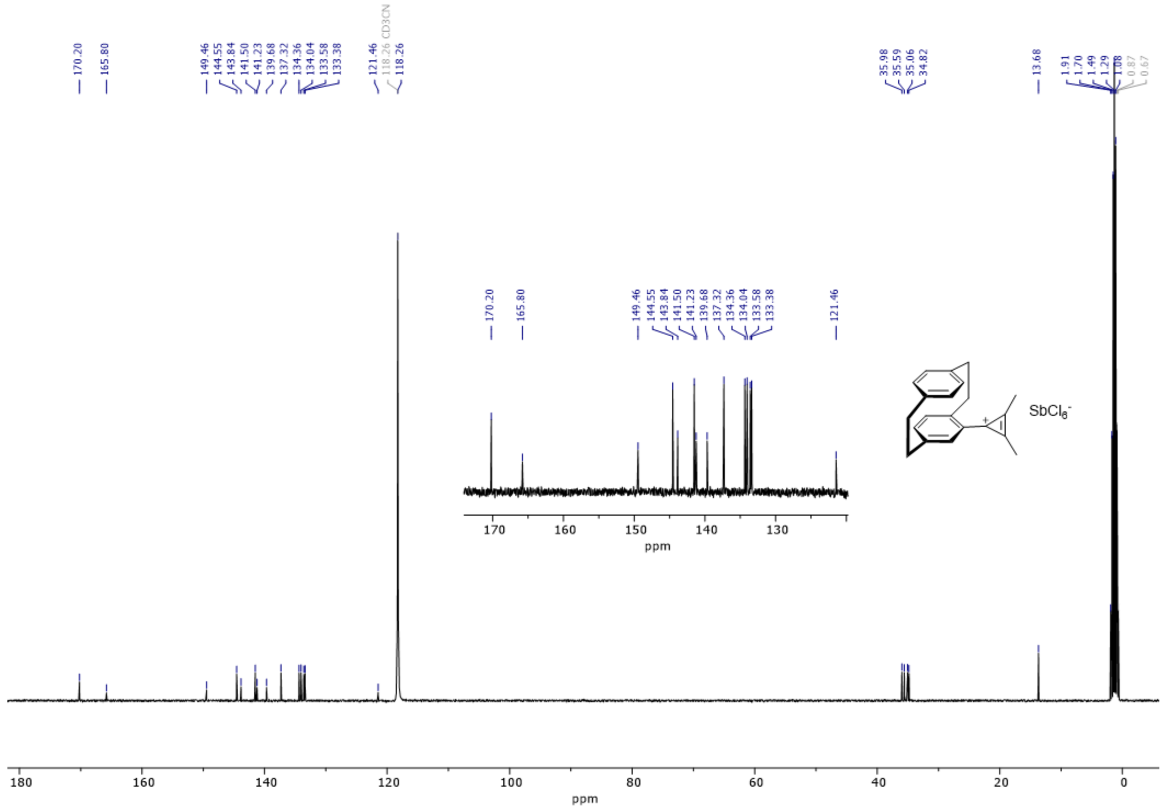

(*rac*)-1^2-(2,3-Dipropylcycloprop-2-en-1-yl)ium-[2.2]paracyclophane hexachloroantimonate (11b)

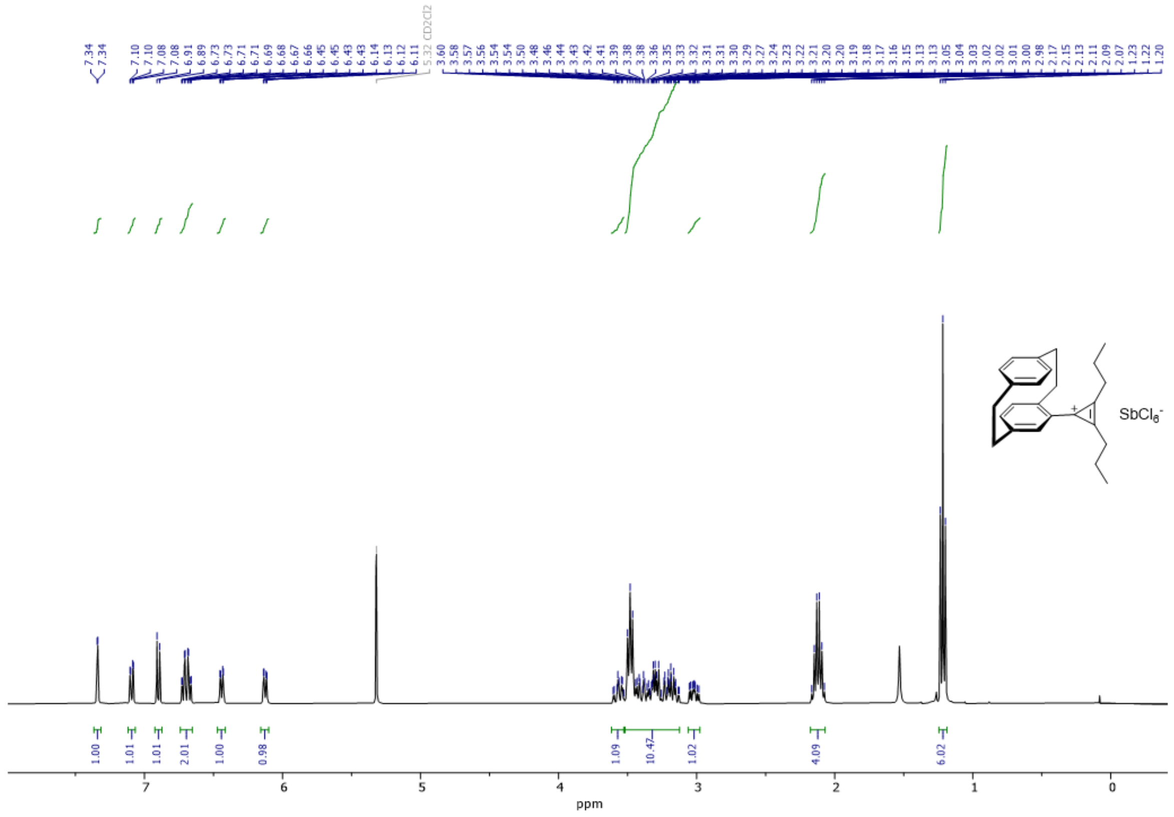

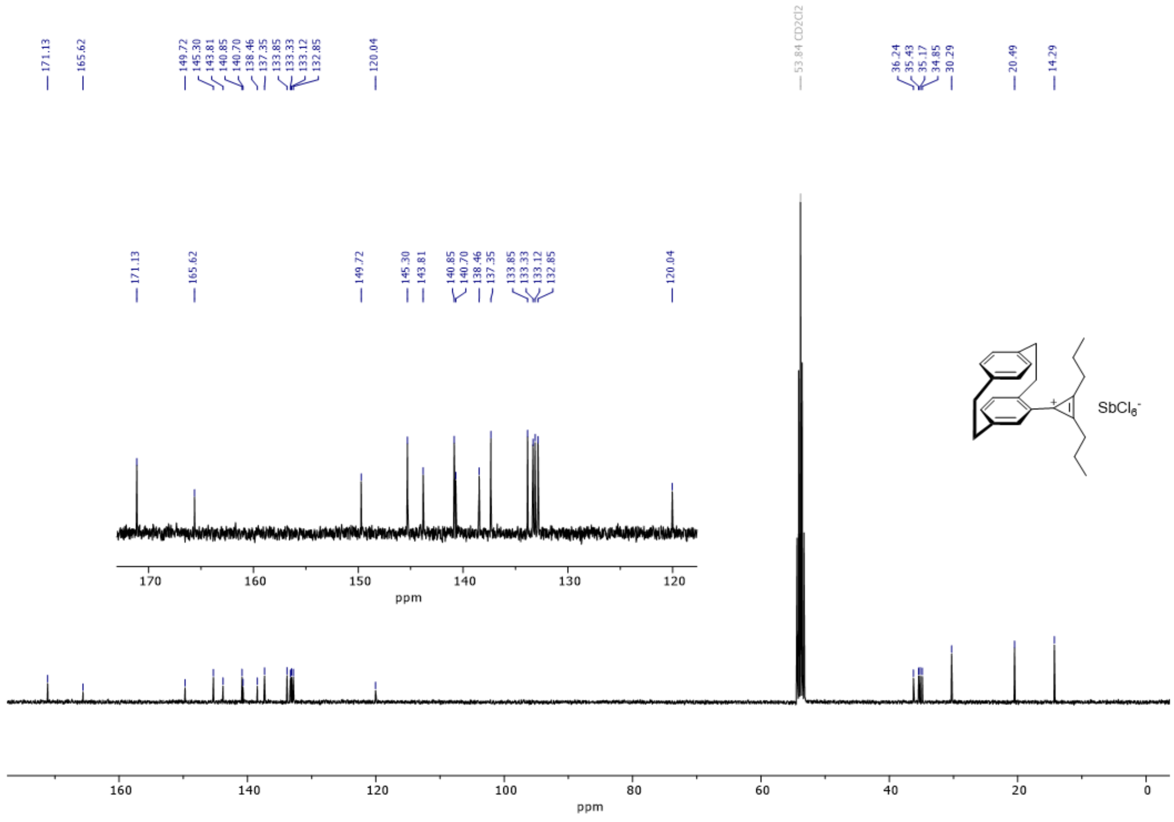

(*rac*)-1²-(2,3-Dibutylcycloprop-2-en-1-yl)-[2.2]paracyclophane hexachloroantimonate (11c)

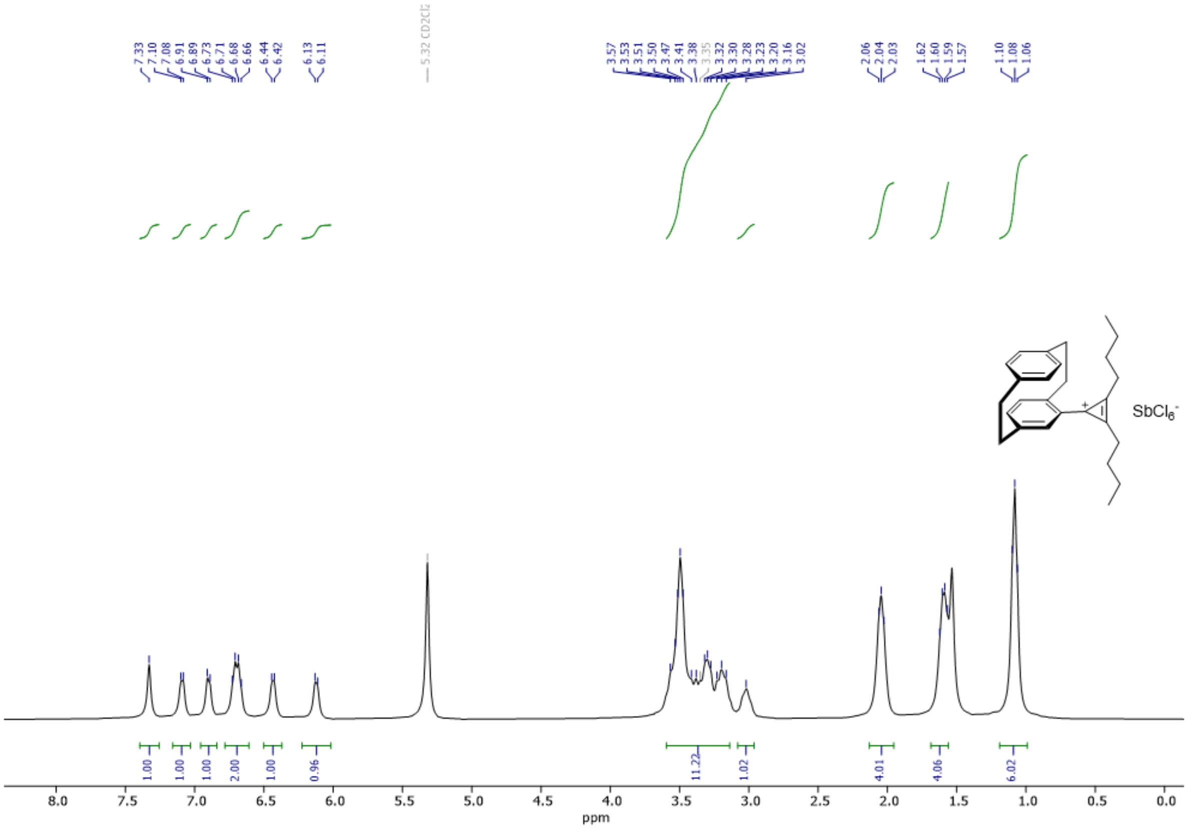

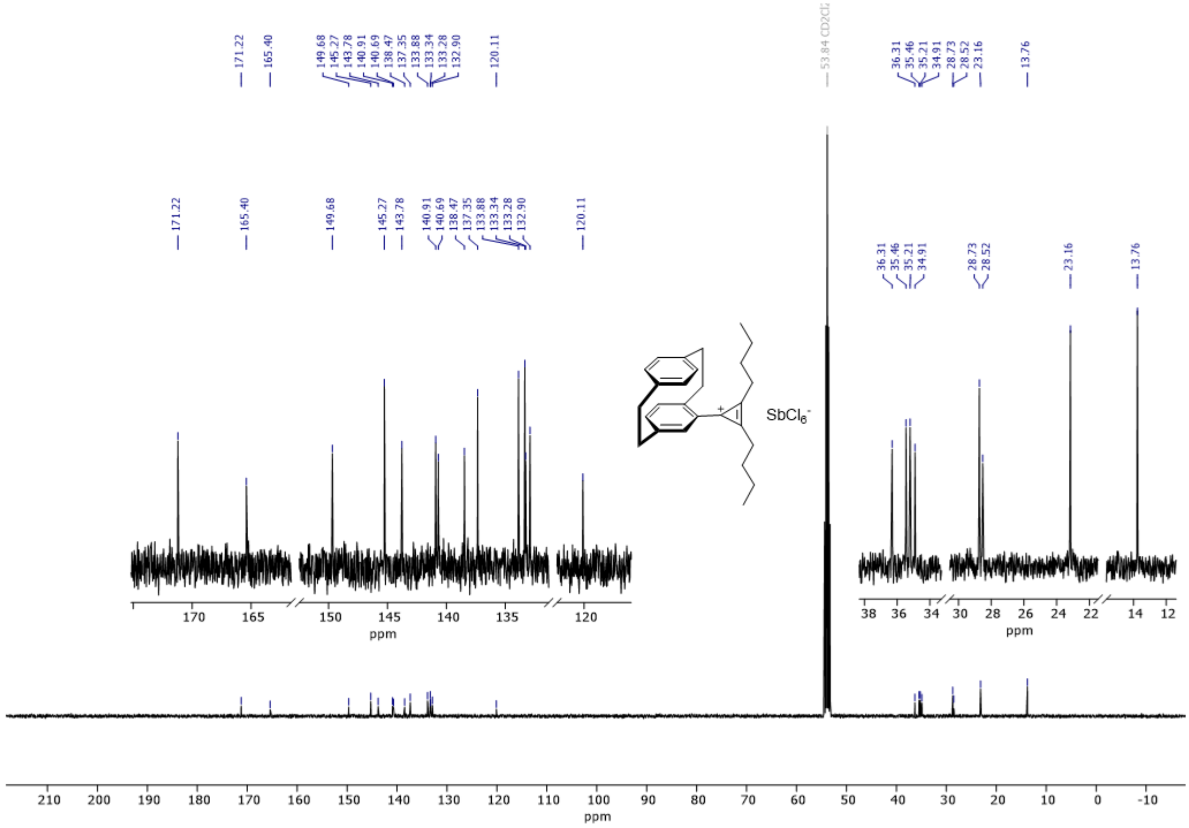

(*rac*)-1²-(2-Ethyl-3-methylcycloprop-2-en-1-yl)-[2.2]paracyclophane hexachloroantimonate (11d)

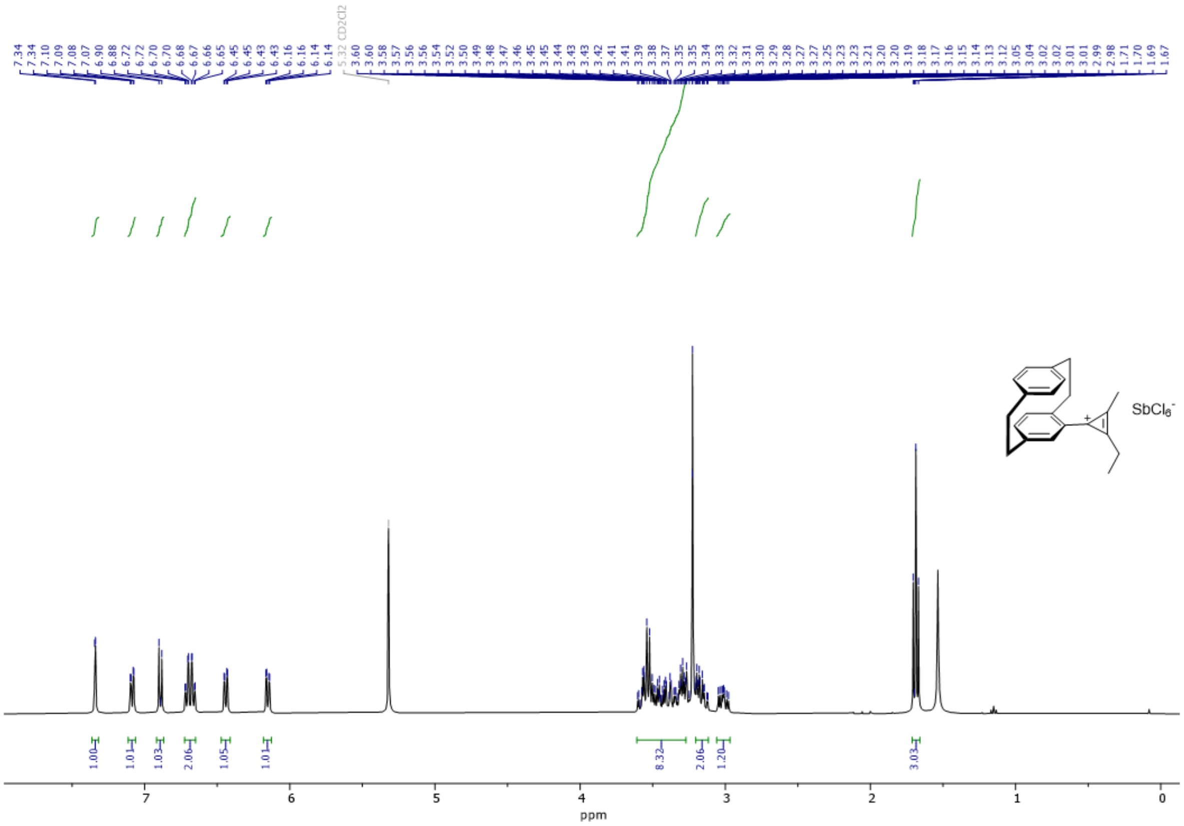

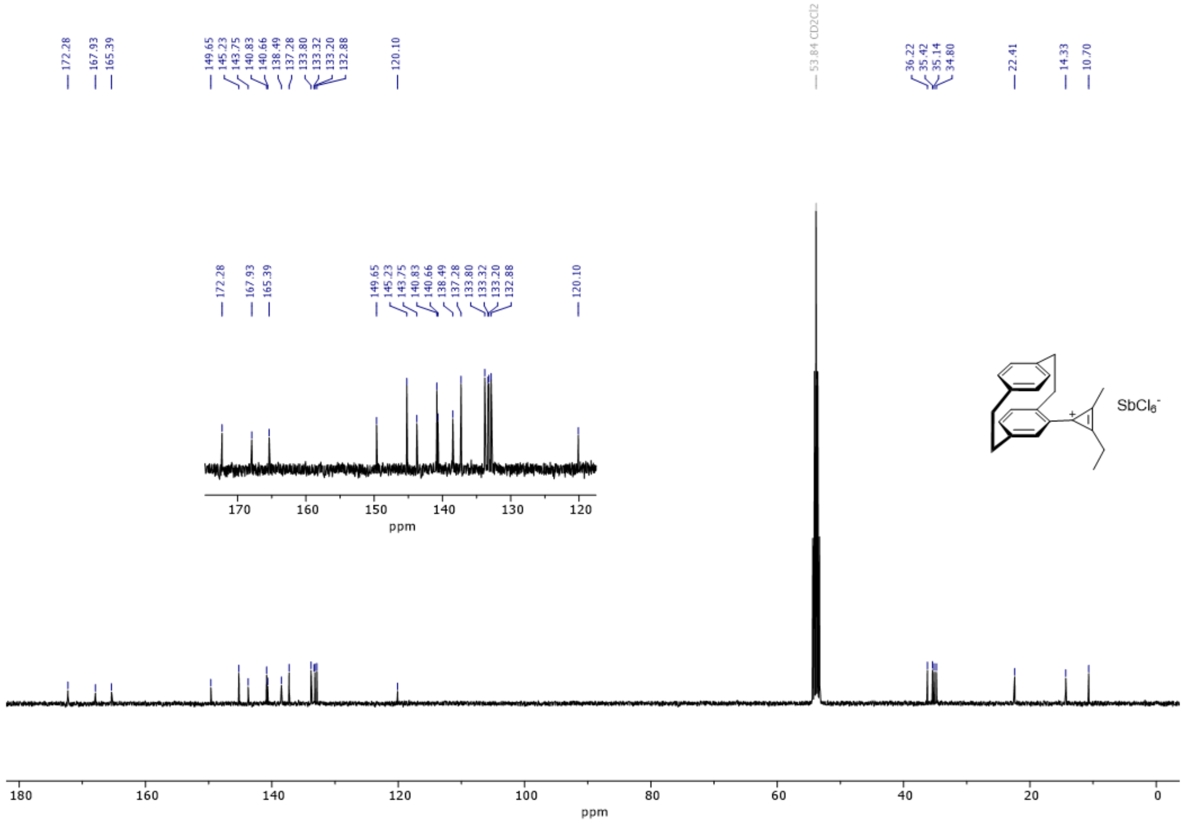

6.2. UV/VIS spectra

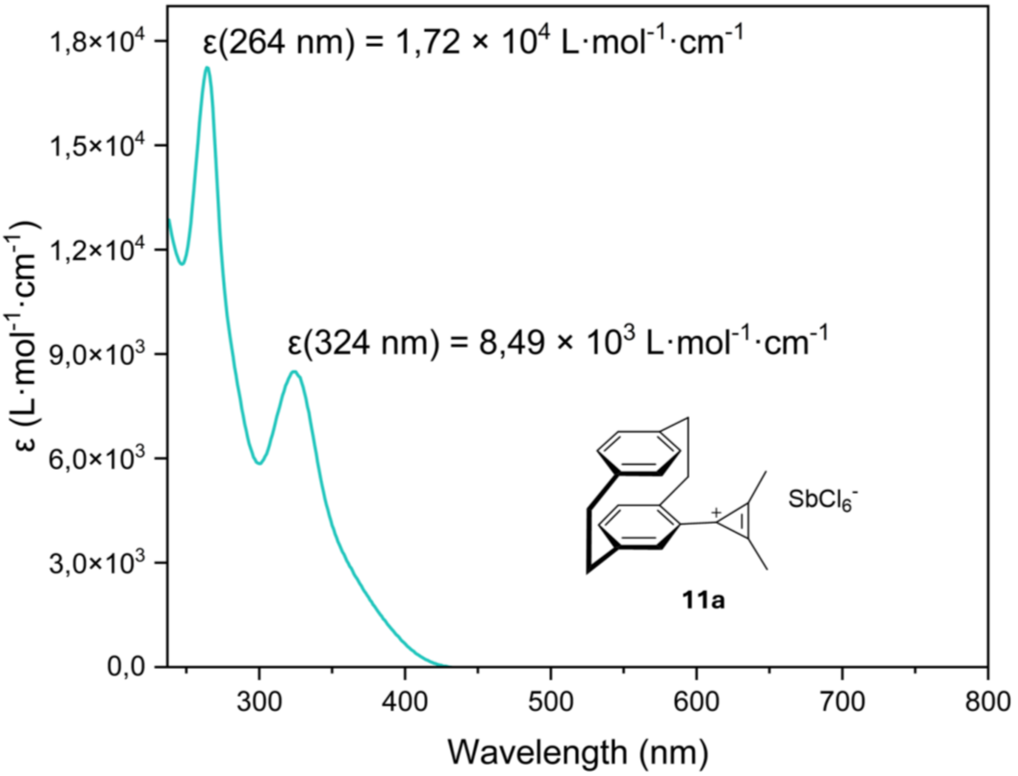

Figure S5: UV/Vis spectrum of **11a** (DCM, c = 2.40 × 10^{-4} M, l = 0.1 cm, 298 K; baseline corrected against solvent blank).

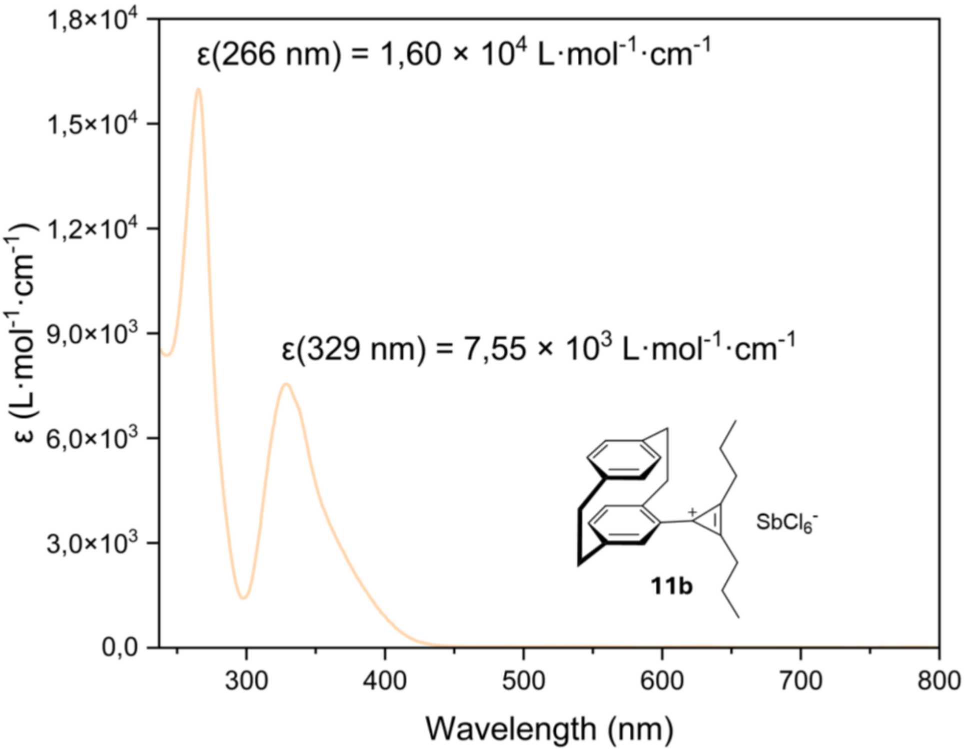

Figure S6: UV/Vis spectrum of **11b** (DCM, c = 6.7 × 10^{-5} M, l = 0.5 cm, 298 K; baseline corrected against solvent blank).

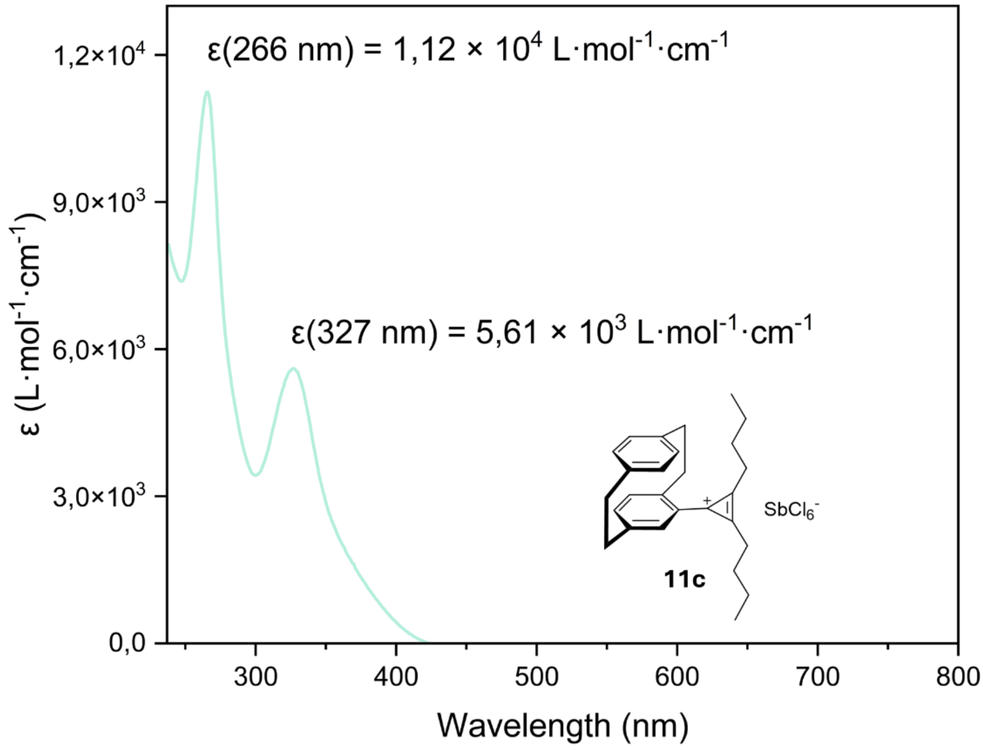

Figure S7: UV/Vis spectrum of **11c** (DCM, c = 9.29 × 10^{-5} M, l = 0.5 cm, 298 K; baseline corrected against solvent blank).

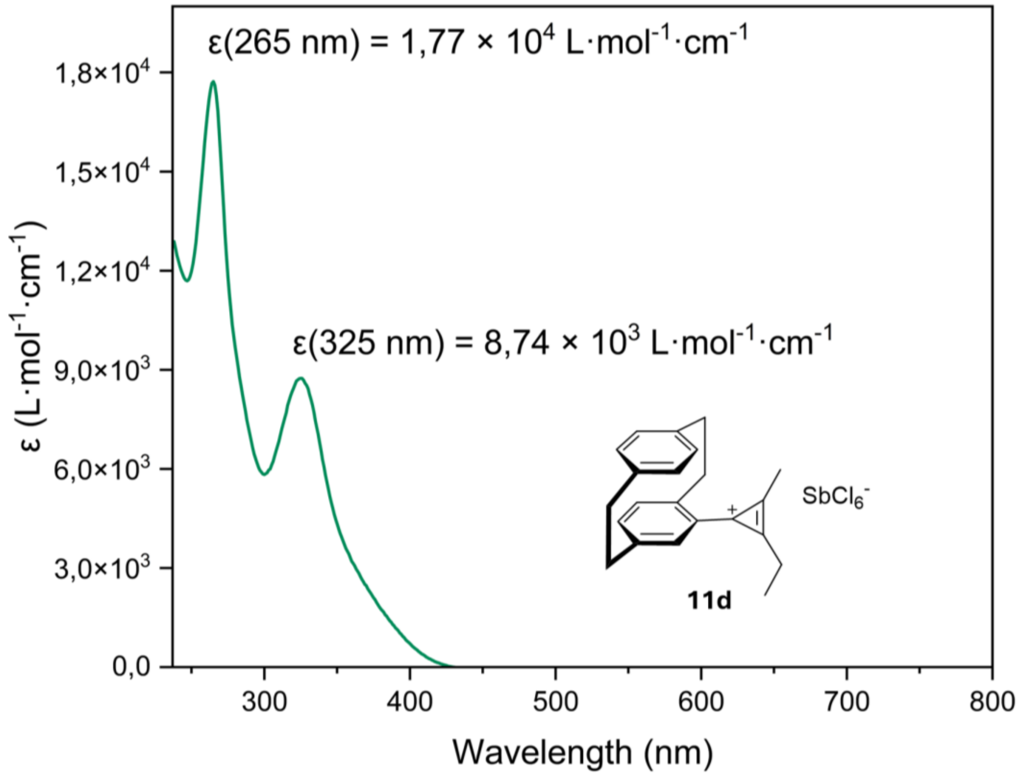

Figure S8: UV/Vis spectrum of **11c** (DCM, c = 2.49 × 10^{-4} M, l = 0.1 cm, 298 K; baseline corrected against solvent blank).

6.3. NOESY Experiment

A NOESY experiment was conducted to differentiate the diastereomers of **3f**. However, no diagnostic correlations were observed. This is attributed to the low rotational barrier of the cyclopropenyl moiety, which leads to time-averaged NOE contacts and prevents a definitive structural assignment of the major and minor isomers.

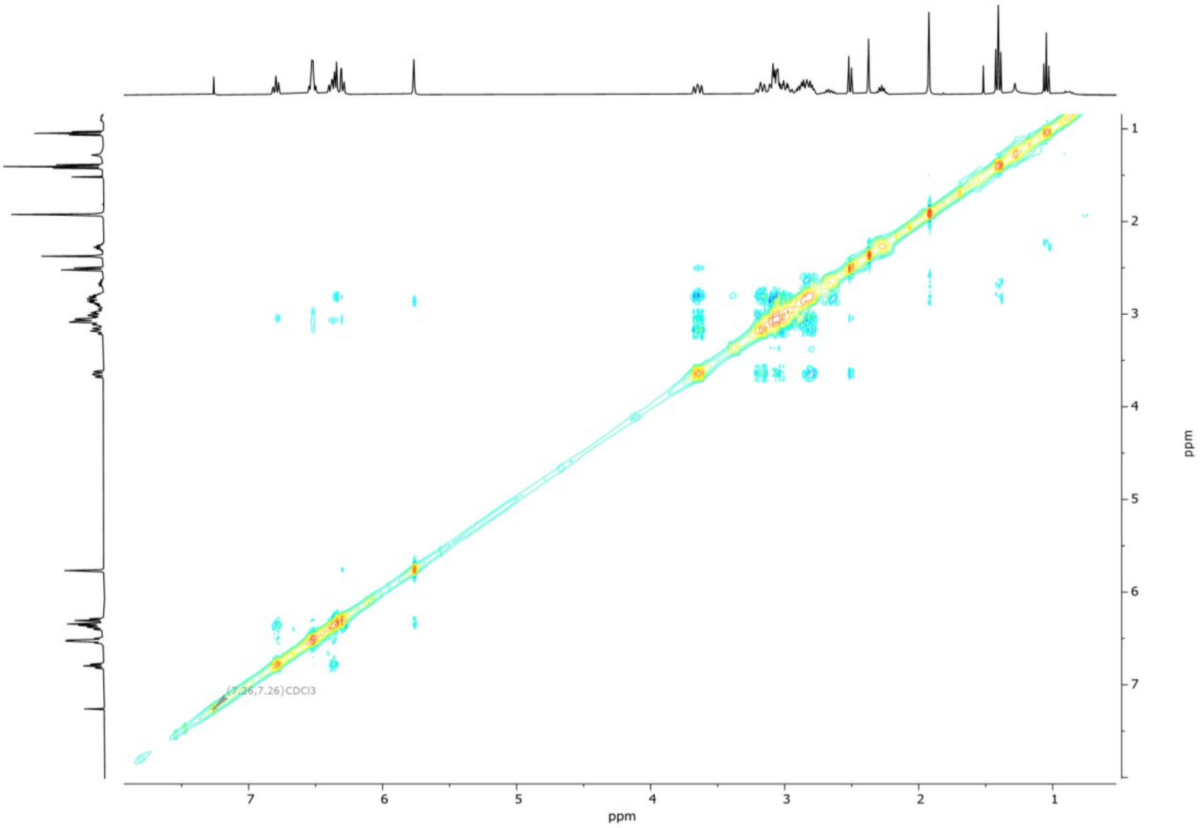

Figure S9: Full 2D NOESY NMR spectrum (400 MHz, chloroform-*d*) of the diastereomeric mixture **3f**.

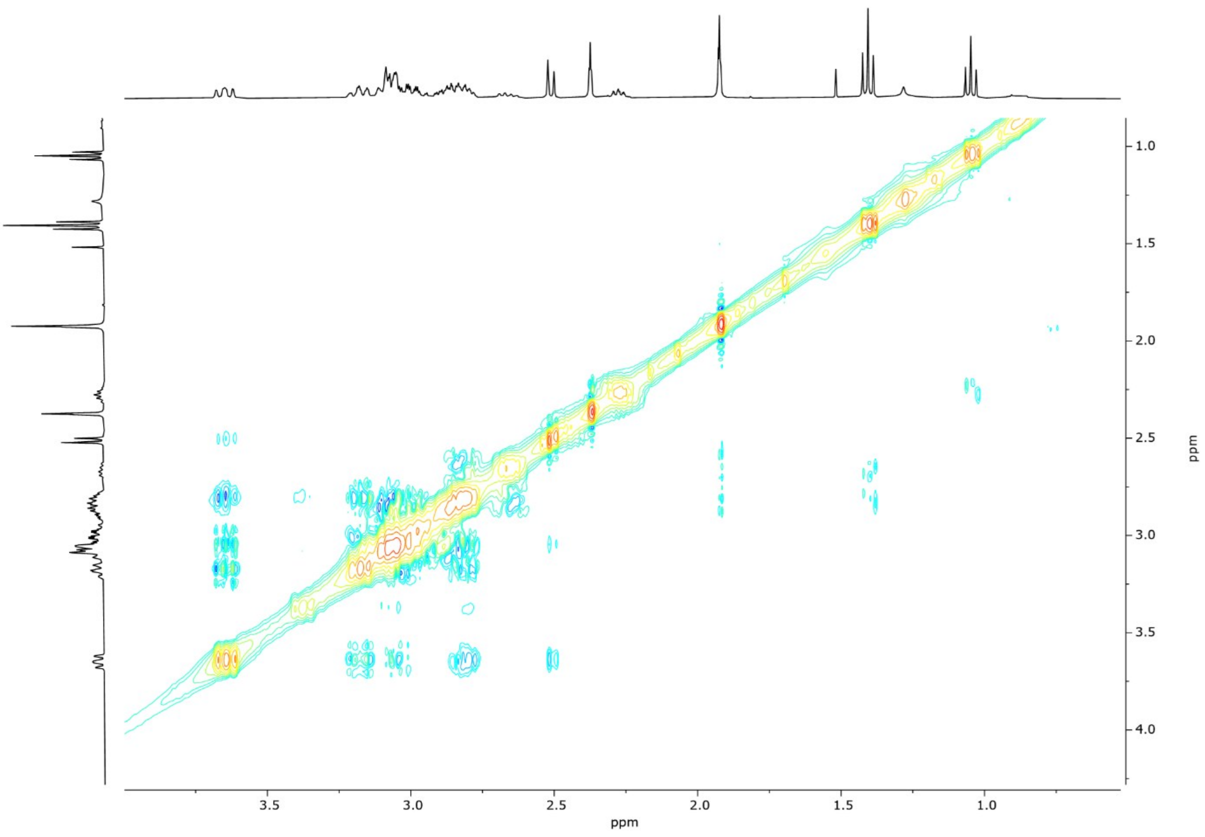

Figure S10: Aliphatic region of the NOESY spectrum of **3f**.

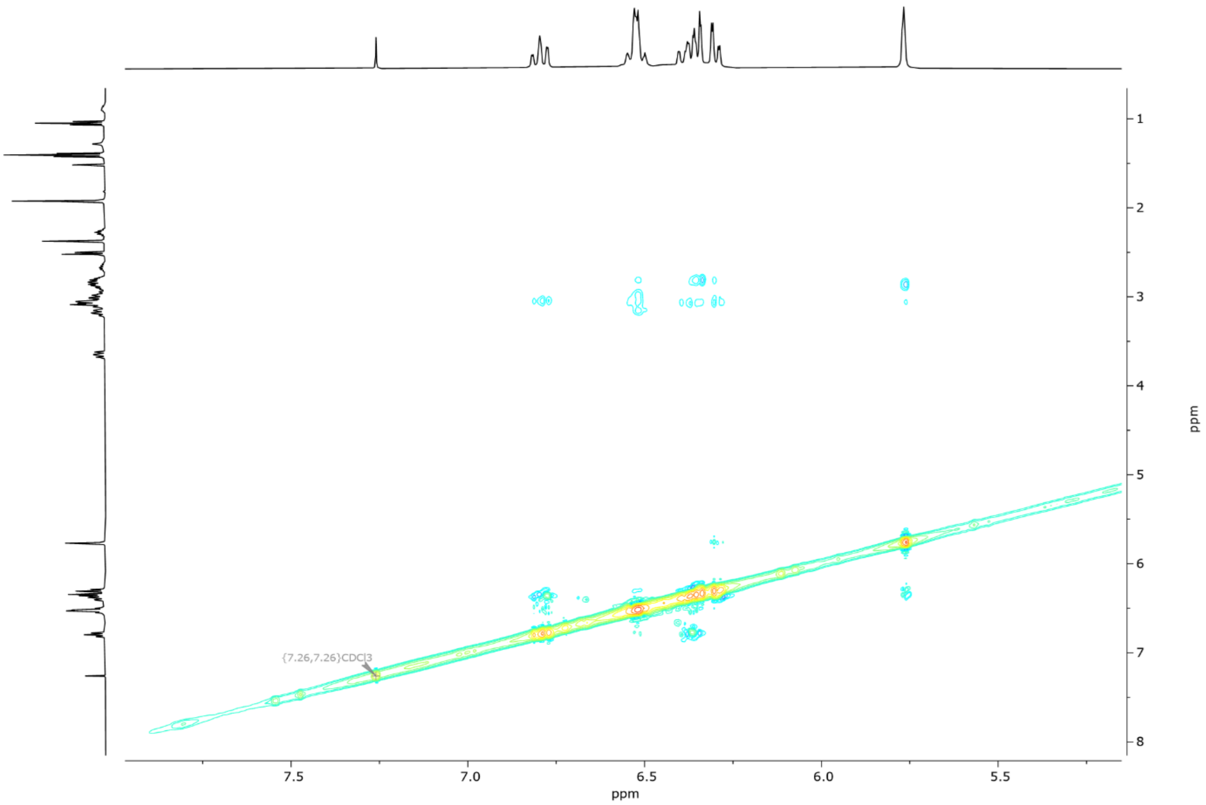

Figure S11: Aromatic region of the NOESY spectrum of **3f**.

7. References

(1) Köhler, T. *Chemotion Repository* **2026**, https://dx.doi.org/10.14272/collection/TIK_2024-02-02

(2) Chemotion Repository https://www.chemotion-repository.net/home/welcome

(3) Huang, P.-C.; Lin, C.-L.; Tremouilhac, P.; Jung, N.; Bräse, S. *Nat. Protoc.* **2024**, 1–2

(4) Köhler, T.; Fuhr, O.; Bräse, S. *Org. Chem. Front.* **2025**, *12*, 3546–3550

(5) Bi, X.; Liu, S.; Yang, Y.; Song, Q.; Liu, Z.; Sivaguru, P.; Zhang, Y.; Ruiter, G.; Anderson, *Nat. Commun.* **2024**, *15*, 9998

(6) Dolomanov, O. V.; Bourhis, L. J.; Gildea, R. J.; Howard, J. a. K.; Puschmann, H. *J. Appl. Crystallogr.* **2009**, *42*, 339–341

(7) Sheldrick, G. M. *Acta Crystallogr. Sect. Found. Adv.* **2015**, *71*, 3–8

(8) Sheldrick, G. M. *Acta Crystallogr. Sect. C Struct. Chem.* **2015**, *71*, 3–8

(9) Neese, F. *WIREs Comput. Mol. Sci.* **2012**, *2*, 73–78

(10) de Souza, B. *Angew. Chem. Int. Ed.* **2025**, *64*, e202500393

(11) Bannwarth, C.; Ehlert, S.; Grimme, S. *J. Chem. Theory Comput.* **2019**, *15*, 1652–1671

(12) Ásgeirsson, V.; Birgisson, B. O.; Bjornsson, R.; Becker, U.; Neese, F.; Riplinger, C.; Jónsson, H. *J. Chem. Theory Comput.* **2021**, *17*, 4929–4945

(13) Adamo, C.; Barone, V. *J. Chem. Phys.* **1999**, *110*, 6158–6170

(14) Barone, V.; Cossi, M. *J. Phys. Chem. A* **1998**, *102*, 1995–2001
